# Supplementary material for: Genetic activation of ERK2 recapitulates core neurodevelopmental features of Rasopathy syndromes in mice
Source: HGG Adv. 2026 Apr 28;7(3):100621. doi: 10.1016/j.xhgg.2026.100621 (PMC13234240; doi:10.1016/j.xhgg.2026.100621)
Supplement: Document S1. Figures S1–S8 [file mmc1.pdf]

HGGA, Volume 7

Supplemental information

Genetic activation of ERK2 recapitulates core neurodevelopmental features of Rasopathy syndromes in mice

Kassidy E. Grover, Zoe R. Cappel, Avery M. Volz, Evelin M. Cotella, Kelly Smallwood, Christine A. Berryhill, Kimaya R. Raje, Austen A. Fisher, Mary Claire T. Casper, Diana Nardini, Tilat A. Rizvi, Rosa M. Salazar, Ashley Wooten, Michael T. Williams, Charles V. Vorhees, Lindsey E. Romick, Kenneth D. Greis, Yueh-Chiang Hu, Linde A. Miles, Steven P. Angus, Nancy Ratner, Carlos E. Prada, K. Nicole Weaver, Ronald R. Waclaw, and J. Elliott Robinson

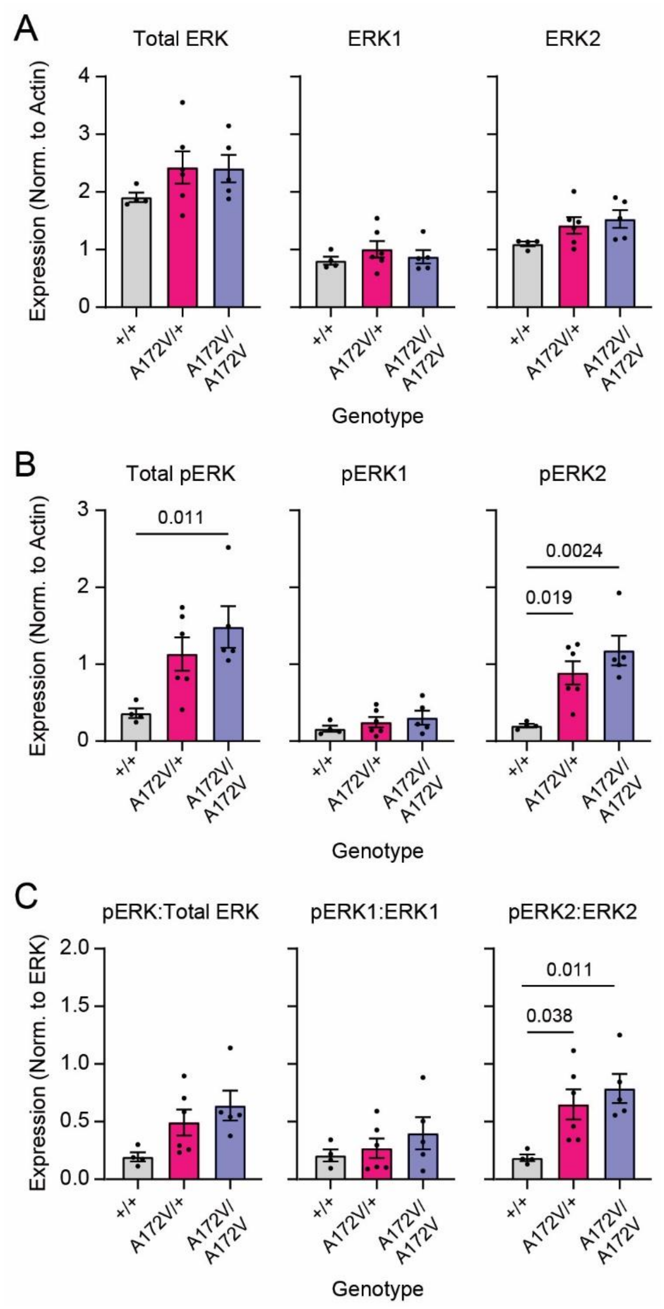

Figure S1. Analysis of ERK phosphorylation in PD7 livers in *Mapk1* mutant and control mice. (A) There were no significant genotypic differences in total ERK (*left*; $n_{+/+}$ = 4; $n_{A172V/+}$ = 6; $n_{A172V/A172V}$ = 5; one-way ANOVA; $F_{2,12}$ = 1.28, p = 0.31), ERK1 (*middle*; $n_{+/+}$ = 4; $n_{A172V/+}$ = 6; $n_{A172V/A172V}$ = 5; one-way ANOVA; $F_{2,12}$ = 0.65, p = 0.54), or ERK2 (right; $n_{+/+}$ = 4; $n_{A172V/+}$ = 6; $n_{A172V/A172V}$ = 5; one-way ANOVA; $F_{2,12}$ = 2.37, p = 0.14) in PD7 liver samples, as measured by

Western blot. **(B)** There was a significant increase in total pERK expression in PD7 *Mapk1* mutant livers (*left*; $n_{+/+}$ = 4; $n_{A172V/+}$ = 6; $n_{A172V/A172V}$ = 5; one-way ANOVA; $F_{2,12}$ = 5.95, p = 0.016). Bonferroni *post hoc* analysis showed that pERK was significantly lower in *Mapk1*$^{+/+}$ samples relative to *Mapk1*$^{A172V/A172}$ samples (p = 0.011); this difference did not reach statistical significance in *Mapk1*$^{A172V/+}$ livers relative to control samples (p = 0.064). There were no genotypic differences in pERK1 (*middle*; $n_{+/+}$ = 4; $n_{A172V/+}$ = 6; $n_{A172V/A172V}$ = 5; one-way ANOVA; $F_{2,12}$ = 0.85, p = 0.45). pERK2 levels were higher in *Mapk1* mutant livers relative to control samples (*right*; $n_{+/+}$ = 4; $n_{A172V/+}$ = 6; $n_{A172V/A172V}$ = 5; one-way ANOVA; $F_{2,12}$ = 9.18, p = 0.0038). Bonferroni *post hoc* tests showed a significant increase in heterozygous mutants (p = 0.019) and homozygous mutants (p = 0.0024). **(C)** There were no genotypic differences in the pERK:total ERK ratio (*left*; $n_{+/+}$ = 4; $n_{A172V/+}$ = 6; $n_{A172V/A172V}$ = 5; one-way ANOVA; $F_{2,12}$ = 3.65, p = 0.058) or pERK1:ERK1 ratio (*middle*; $n_{+/+}$ = 4; $n_{A172V/+}$ = 6; $n_{A172V/A172V}$ = 5; one-way ANOVA; $F_{2,12}$ = 0.84, p = 0.46). The pERK2:ERK2 ratio was significantly increased in *Mapk1* mutants relative to wildtype controls (*right*; $n_{+/+}$ = 4; $n_{A172V/+}$ = 6; $n_{A172V/A172V}$ = 5; one-way ANOVA; $F_{2,12}$ = 6.17, p = 0.014). pERK2:ERK2 was higher in *Mapk1*$^{A172V/+}$ (Bonferroni *post hoc* test; p = 0.038) and *Mapk1*$^{A172V/A172V}$ livers (Bonferroni *post hoc* test; p = 0.011) relative to *Mapk1*$^{+/+}$ samples. Data is presented as mean ± SEM.

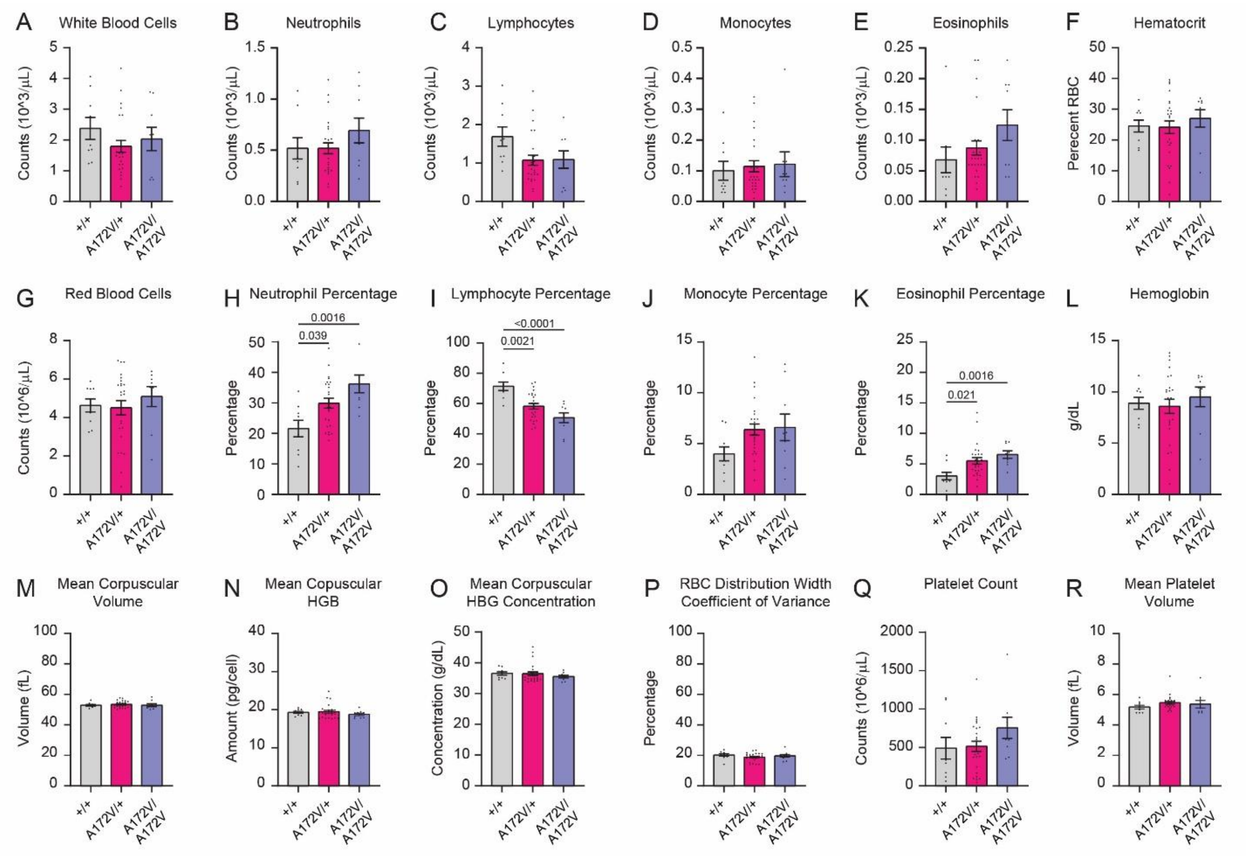

Figure S2. Hematological testing in *Mapk1* mutant and control mice. **(A)** At two weeks of age, there were no significant differences in white blood cell (WBC) counts between in *Mapk1* mutant and control mice ($n_{+/+}$ = 9; $n_{A172V/+}$ = 25; $n_{A172V/A172V}$ = 9; one-way ANOVA; $F_{2,40}$ = 0.45, p = 0.35). **(B-E)** There were no genotypic differences in absolute neutrophil ($n_{+/+}$ = 9; $n_{A172V/+}$ = 25; $n_{A172V/A172V}$ = 9; one-way ANOVA; $F_{2,40}$ = 0.61, p = 0.31), lymphocyte ($n_{+/+}$ = 9; $n_{A172V/+}$ = 25; $n_{A172V/A172V}$ = 9; one-way ANOVA; $F_{2,40}$ = 0.27, p = 0.069), monocyte ($n_{+/+}$ = 9; $n_{A172V/+}$ = 25; $n_{A172V/A172V}$ = 9; Kruskal-Wallis test; H_3 = 1.26, p = 0.53), or eosinophil counts ($n_{+/+}$ = 9; $n_{A172V/+}$ = 25; $n_{A172V/A172V}$ = 9; Kruskal-Wallis test; H_3 = 2.82, p = 0.24). **(F-G)** There were no genotypic differences in hematocrit ($n_{+/+}$ = 9; $n_{A172V/+}$ = 25; $n_{A172V/A172V}$ = 9; one-way ANOVA; $F_{2,40}$ = 0.33, p = 0.72) or red blood cell (RBC) count ($n_{+/+}$ = 9; $n_{A172V/+}$ = 25; $n_{A172V/A172V}$ = 9; one-way ANOVA; $F_{2,40}$ = 0.41, p = 0.67). **(H)** There was a significant increase in the percentage of neutrophils in *Mapk1* mutants ($n_{+/+}$ = 9; $n_{A172V/+}$ = 25; $n_{A172V/A172V}$ = 9; one-way ANOVA; $F_{2,40}$ = 7.21, p = 0.0021).

Bonferroni *post hoc* analysis revealed a significant increase in both *Mapk1*$^{A172V/+}$ (p = 0.039) and *Mapk1*$^{A172V/A172}$ (p = 0.0016) neutrophil percentage compared to wild-type littermates. **(I)** There was a significant decrease in the percentage of lymphocytes ($n_{+/+}$ = 9; $n_{A172V/+}$ = 25; $n_{A172V/A172V}$ = 9; one-way ANOVA; $F_{2,40}$ = 11.87, p < 0.0001) in *Mapk1*$^{A172V/+}$ (Bonferroni *post hoc* test; p = 0.0021) and *Mapk1*$^{A172V/A172V}$ (Bonferroni *post hoc* test; p < 0.0001) mice. **(J)** There was no genotypic difference in monocyte percentage ($n_{+/+}$ = 9; $n_{A172V/+}$ = 25; $n_{A172V/A172V}$ = 9; one-way ANOVA; $F_{2,40}$ = 2.52, p = 0.093). **(K)** There was a significant increase in the percentage of eosinophils ($n_{+/+}$ = 9; $n_{A172V/+}$ = 25; $n_{A172V/A172V}$ = 9; Kruskal-Wallis test; H_3 = 12.57, p = 0.0019) in both *Mapk1*$^{A172V/+}$ mice (Dunn's *post hoc* test; p = 0.021) and *Mapk1*$^{A172V/A172V}$ mice (Dunn's *post hoc* test; p = 0.0016). **(L-O)** There were no genotypic differences in hemoglobin (HGB) concentration ($n_{+/+}$ = 9; $n_{A172V/+}$ = 25; $n_{A172V/A172V}$ = 9; one-way ANOVA; $F_{2,40}$ = 0.30, p = 0.74), mean corpuscular volume ($n_{+/+}$ = 9; $n_{A172V/+}$ = 25; $n_{A172V/A172V}$ = 9; one-way ANOVA; $F_{2,40}$ = 0.38, p = 0.68), mean corpuscular HGB ($n_{+/+}$ = 9; $n_{A172V/+}$ = 25; $n_{A172V/A172V}$ = 9; Kruskal-Wallis test; H_3 = 1.24, p = 0.54) or corpuscular HBG concentration ($n_{+/+}$ = 9; $n_{A172V/+}$ = 25; $n_{A172V/A172V}$ = 9; Kruskal-Wallis test; H_3 = 1.58, p = 0.45). **(P)** There were no genotypic differences in the coefficient of variation of RBC distribution width ($n_{+/+}$ = 9; $n_{A172V/+}$ = 25; $n_{A172V/A172V}$ = 9; one-way ANOVA; $F_{2,40}$ = 0.89, p = 0.42). **(Q-R)** There were no genotypic differences in platelet count ($n_{+/+}$ = 9; $n_{A172V/+}$ = 25; $n_{A172V/A172V}$ = 9; one-way ANOVA; $F_{2,40}$ = 1.60, p = 0.22) or average volume ($n_{+/+}$ = 9; $n_{A172V/+}$ = 25; $n_{A172V/A172V}$ = 9; Kruskal-Wallis test; H_3 = 3.82, p = 0.15). Data is presented as mean ± SEM.

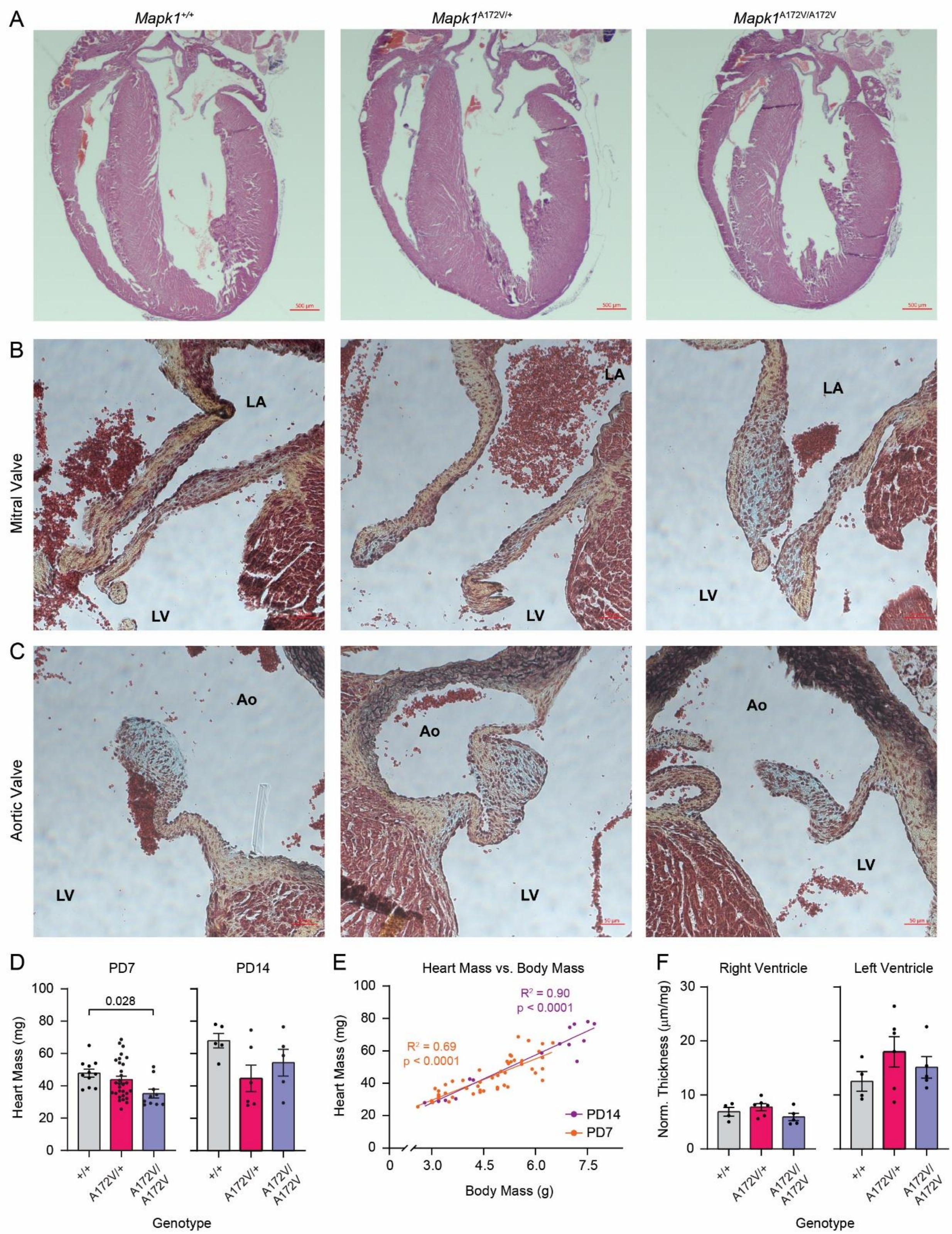
A
Mapk1+/+
Mapk1A172V/+
Mapk1A172V/A172V
B
Mitral Valve
LA
LV
C
Aortic Valve
Ao
LV
D
PD7
PD14
0.028
Heart Mass (mg)
+/+
A172V/+
A172V/ A172V
Genotype
E
Heart Mass vs. Body Mass
R2 = 0.90
p < 0.0001
R2 = 0.69
p < 0.0001
PD14
PD7
Heart Mass (mg)
Body Mass (g)
F
Right Ventricle
Left Ventricle
Norm. Thickness (μm/mg)
Genotype

55

Figure S3. Myocardial and valvular analysis of *Mapk1* mutant and control hearts. (A-C) Representative cross-sectional images of the myocardium (**A**), mitral valve (**B**), and aortic valve **(C)** from 2-week-old *Mapk1*$^{+/+}$ (*left*), *Mapk1*$^{A172V/+}$ (*middle*), and *Mapk1*$^{A172V/A172V}$ mice (*right*) hearts. **(D)** There was a significant difference in the heart weight of *Mapk1* mice at PD7 (*left*; $n_{+/+} = 11$, $n_{A172V/+} = 30$, $n_{A172V/A172V} = 10$; one-way ANOVA; $F_{2,48} = 3.89$, $p = 0.027$) with a significant reduction in *Mapk1*$^{A172V/A172V}$ heart mass compared to wild-type littermates (Bonferroni *post hoc* test; $p = 0.028$). Genotypic differences in heart mass at PD14 did not reach statistical significance (*right*; $n_{+/+} = 5$, $n_{A172V/+} = 6$, $n_{A172V/A172V} = 5$; one-way ANOVA; $F_{2,13} = 2.57$, $p = 0.11$). **(E)** There was a significant correlation between heart mass and body mass at PD7 ($R^2(49) = 0.69$, $p < 0.0001$) and PD14 ($R^2(13) = 0.90$, $p < 0.0001$). **(F)** There was no significant change in normalized right ventricular (one-way ANOVA; $F_{2,12} = 1.82$, $p = 0.20$) or left ventricular thickness (one-way ANOVA; $F_{2,12} = 1.25$, $p = 0.32$) between genotypes. Scale bar for **A** is 500 μm, and the scale bars for **B-C** are 50 μm. Data is presented as mean ± SEM.

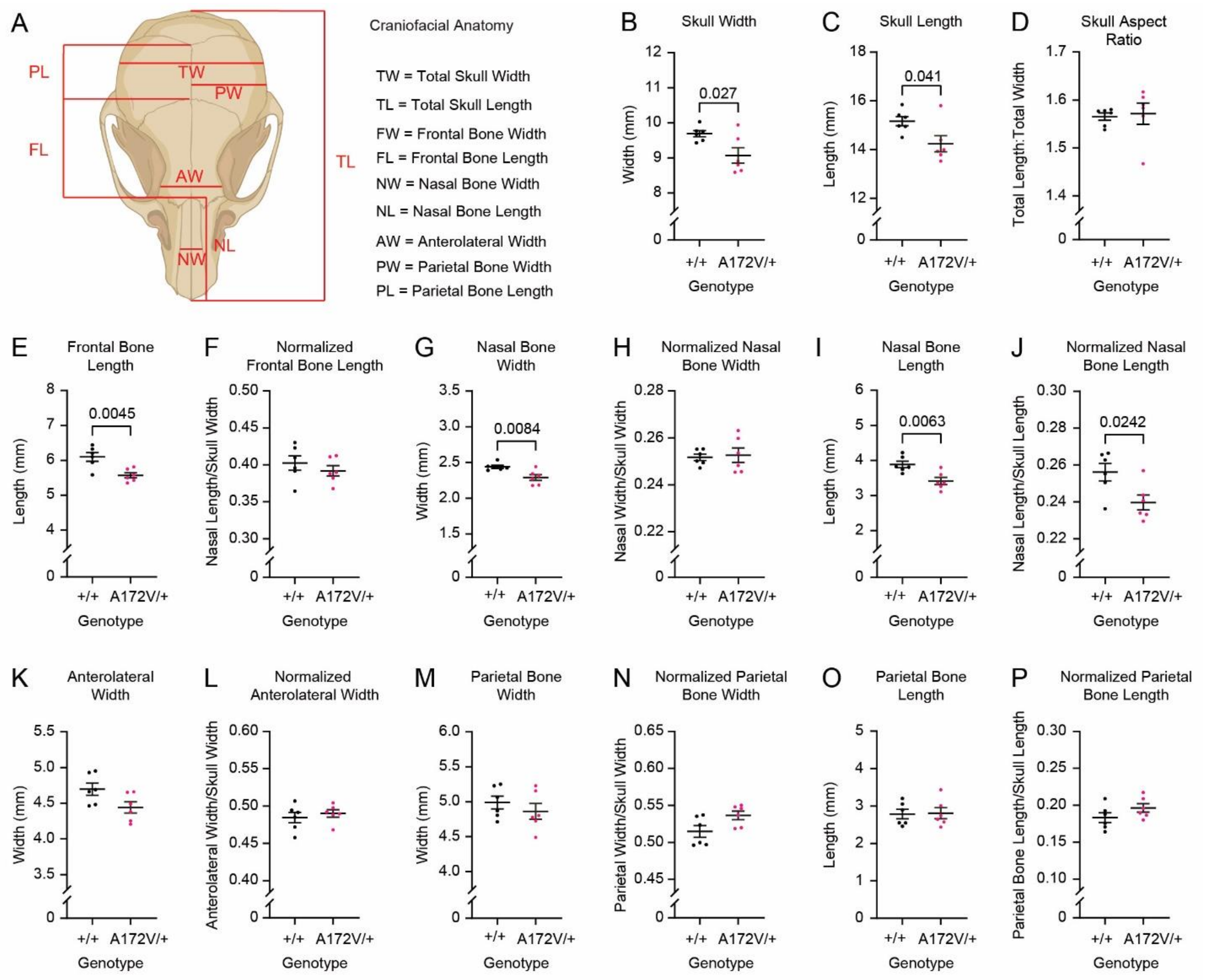

Figure S4. Craniofacial analysis in *Mapk1* mutant and control mice. (A) Schematic showing skull features measures in PD7 *Mapk1*$^{+/+}$ and *Mapk1*$^{A172V/+}$ mice. **(B-C)** There was a significant reduction in the overall width ($n_{+/+}$ = 6; $n_{A172V/+}$ = 6; unpaired t-test; t_{10}= 2.59, p = 0.027) and length of skulls in heterozygous mutant mice (Mann-Whitney U-test; $U(n_{+/+}$ = 6, $n_{A172V/+}$ = 6) = 5, p = 0.041). **(D)** There was no difference in the skull aspect ratio (length:width) between genotypes (Mann-Whitney U-test; $U(n_{+/+}$ = 6, $n_{A172V/+}$ = 6) = 10, p = 0.24). **(E-F)** There was a significant reduction in frontal bone length in *Mapk1*$^{A172V/+}$ mice ($n_{+/+}$ = 6; $n_{A172V/+}$ = 6; unpaired t-test; t_{10}= 3.65, p = 0.0045) that was not significant after normalizing to the total skull length ($n_{+/+}$ = 6; $n_{A172V/+}$ = 6; unpaired t-test; t_{10}= 0.88, p = 0.40). **(G-H)** There was a significant reduction in nasal bone

width ($n_{+/+}$ = 6; $n_{A172V/+}$ = 6; unpaired t-test; t_{10}= 3.27, p = 0.0084) in *Mapk1*$^{A172V/+}$ mice that was not significant after normalizing to the overall skull width ($n_{+/+}$ = 6; $n_{A172V/+}$ = 6; unpaired t-test; t_{10}= 0.27, p = 0.80). **(I-J)** There was a significant decrease in the nasal bone length in *Mapk1*$^{A172V/+}$ mice compared to wildtype littermates ($n_{+/+}$ = 6; $n_{A172V/+}$ = 6; unpaired t-test; t_{10}= 3.44, p = 0.0063) that persisted after it was normalized to the total skull length ($n_{+/+}$ = 6; $n_{A172V/+}$ = 6; unpaired t-test; t_{10}= 2.65, p = 0.024). **(K-P)** There were no significant differences in the skull anterolateral width ($n_{+/+}$ = 6; $n_{A172V/+}$ = 6; unpaired t-test; t_{10}= 2.19, p = 0.054), normalized anterolateral width ($n_{+/+}$ = 6; $n_{A172V/+}$ = 6; unpaired t-test; t_{10}= 0.63, p = 0.54), parietal bone width ($n_{+/+}$ = 6; $n_{A172V/+}$ = 6; unpaired t-test; t_{10}= 0.86, p = 0.41), normalized parietal bone width (Mann-Whitney U-test; $U(n_{+/+}$ = 6, $n_{A172V/+}$ = 6) = 7, p = 0.093), parietal bone length (unpaired t-test; t_{10}= 0.11, p = 0.92), and normalized parietal bone length ($n_{+/+}$ = 6; $n_{A172V/+}$ = 6; unpaired t-test; t_{10}= 1.48, p = 0.17) between *Mapk1*$^{A172V/+}$ and *Mapk1*$^{+/+}$ mice. Data is presented as mean ± SEM.

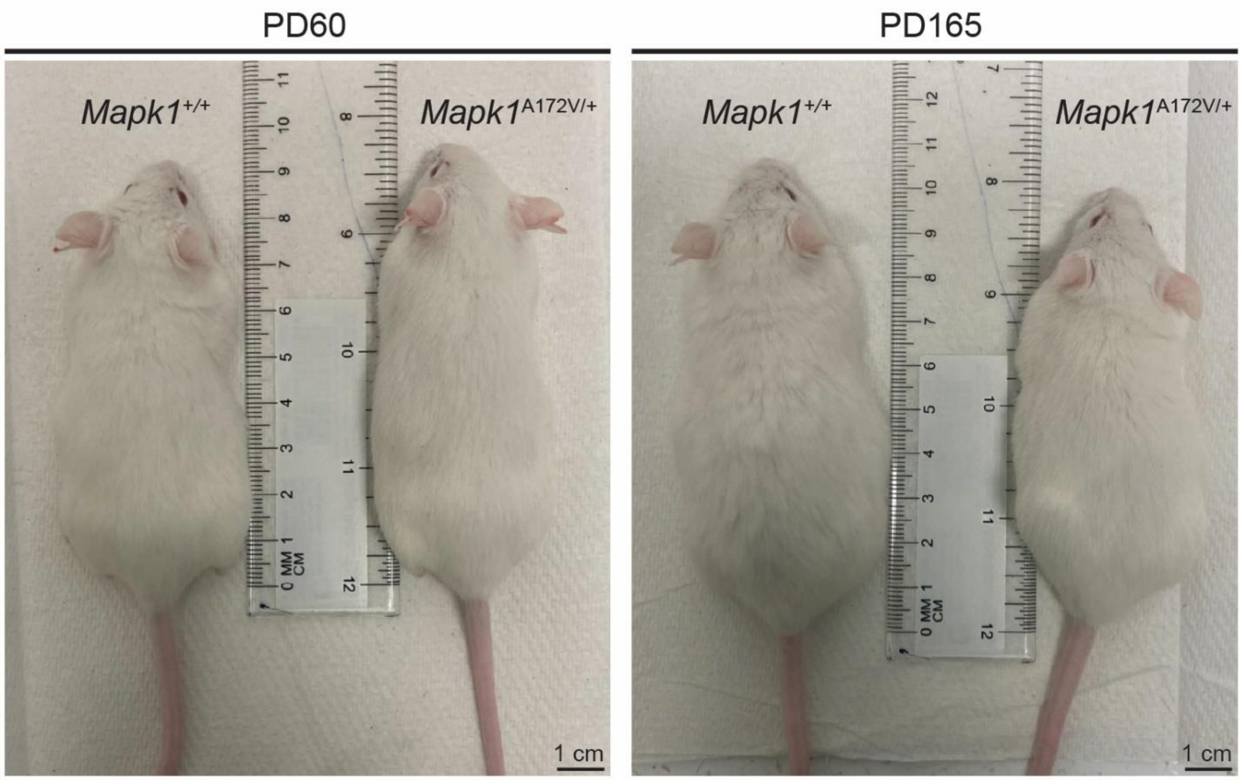

Figure S5. Representative images showing male *Mapk1*$^{+/+}$ and *Mapk1*$^{A172V/+}$ mice at PD60 (*left*) and PD165 (*right*). Scale bar is 1cm.

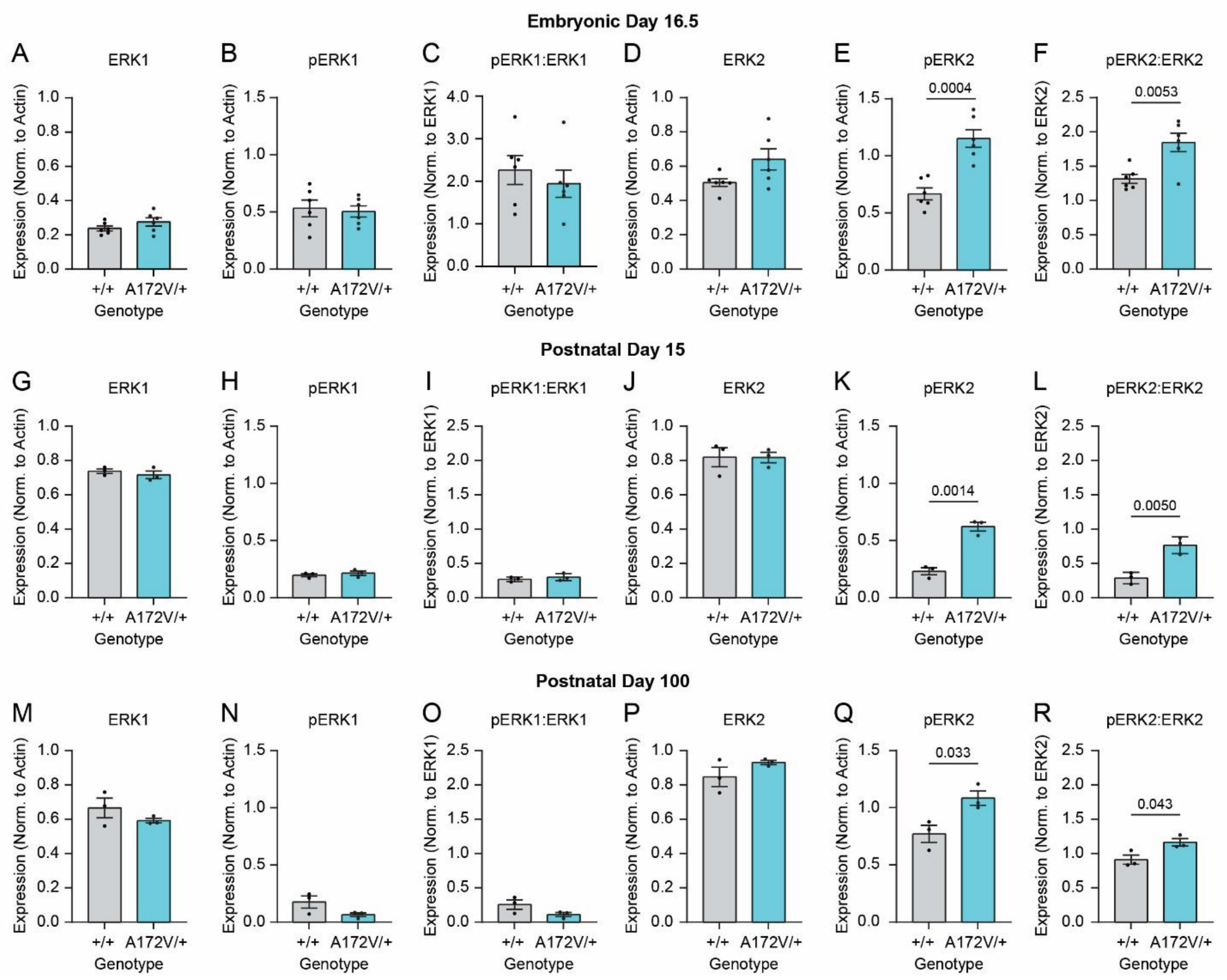

Figure S6. Developmental time course of ERK phosphorylation in the brains of *Mapk1* mutant and control mice. (A-D) At ED16.5, there were no genotypic differences in the level of ERK1 ($n_{+/+}$ = 6; $n_{A172V/+}$ = 6; unpaired t-test; t_{10} = 1.33, p = 0.21), pERK1 ($n_{+/+}$ = 6; $n_{A172V/+}$ = 6; unpaired t-test; t_{10} = 0.305, p = 0.77), the pERK1:ERK1 ratio ($n_{+/+}$ = 6; $n_{A172V/+}$ = 6; unpaired t-test; t_{10} = 0.69, p = 0.51), or total ERK2 ($n_{+/+}$ = 6; $n_{A172V/+}$ = 6; unpaired t-test with Welch's correction; t_{10} = 2.073, p = 0.082) in forebrain samples, as measured by Western blot. **(E-F)** There were significant increases in pERK2 ($n_{+/+}$ = 6; $n_{A172V/+}$ = 6; unpaired t-test; t_{10} = 5.18, p = 0.0004) and the pERK2:ERK2 ratio ($n_{+/+}$ = 6; $n_{A172V/+}$ = 6; unpaired t-test; t_{10} = 3.55, p = 0.0053) in *Mapk1*$^{A172V/+}$ forebrain samples relative to controls at ED16.5. **(G-J)** At PD15, there were no significant genotypic differences in the abundance of ERK1 ($n_{+/+}$ = 3; $n_{A172V/+}$ = 3; unpaired t-test; t_4 = 0.83, p

= 0.45), pERK1 ($n_{+/+}$ = 3; $n_{A172V/+}$ = 3; unpaired t-test; t_4 = 0.72, p = 0.51), pERK1:ERK1 ($n_{+/+}$ = 3; $n_{A172V/+}$ = 3; unpaired t-test; t_4 = 0.89, p = 0.42), and ERK2 ($n_{+/+}$ = 3; $n_{A172V/+}$ = 3; unpaired t-test; t_4 = 0.025, p = 0.98) in forebrain samples. **(K-L)** Significant increases in pERK2 ($n_{+/+}$ = 3; $n_{A172V/+}$ = 3; unpaired t-test; t_4 = 7.88, p = 0.0014) and pERK2:ERK2 ($n_{+/+}$ = 3; $n_{A172V/+}$ = 3; unpaired t-test; t_4 = 5.58, p = 0.005) were observed in *Mapk1*$^{A172V/+}$ forebrain samples at PD15. **(M-P)** At PD100, there were no genotypic differences in ERK1 ($n_{+/+}$ = 3; $n_{A172V/+}$ = 3; unpaired t-test; t_4 = 1.26, p = 0.28), pERK1 ($n_{+/+}$ = 3; $n_{A172V/+}$ = 3; unpaired t-test; t_4 = 1.99, p = 0.12), pERK1:ERK1 ($n_{+/+}$ = 3; $n_{A172V/+}$ = 3; unpaired t-test; t_4 = 1.93, p = 0.13), or ERK2 ($n_{+/+}$ = 3; $n_{A172V/+}$ = 3; unpaired t-test; t_4 = 1.47, p = 0.22) in forebrain samples. Significant increases in pERK2 ($n_{+/+}$ = 3; $n_{A172V/+}$ = 3; unpaired t-test; t_4 = 3.204, p = 0.033) and pERK2:ERK2 ($n_{+/+}$ = 3; $n_{A172V/+}$ = 3; unpaired t-test; t_4 = 2.93, p = 0.043) were observed in *Mapk1*$^{A172V/+}$ forebrain samples at PD100. Data is presented as mean ± SEM.

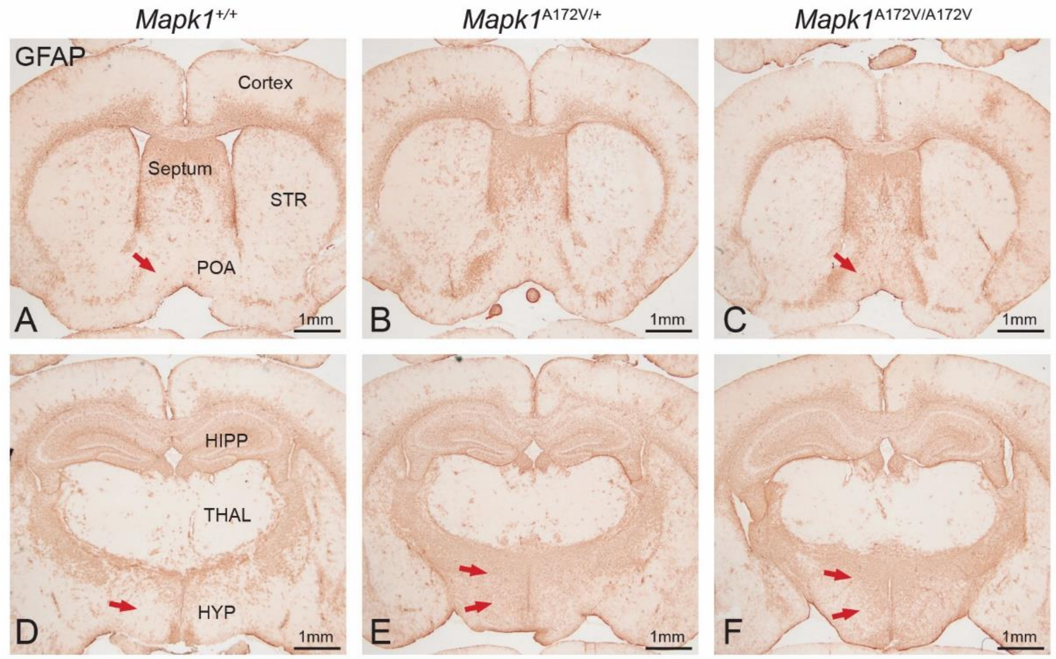

Figure S7. GFAP expression in the brains of *Mapk1* mutant and control mice. (A-C) Images showing GFAP expression (a marker for reactive astrocytes) in different brain structures in 10-week-old **(A)** *Mapk1*$^{+/+}$, **(B)** *Mapk1*$^{A172V/+}$, and **(C)** *Mapk1*$^{A172V/A172V}$ mice, including the cerebral cortex, septum, striatum (STR), and pre-optic area (POA). Red arrows indicate differences in GFAP immunoreactivity in the pre-optic area. **(D-F)** Images showing GFAP expression in **(D)** *Mapk1*$^{+/+}$, **(E)** *Mapk1*$^{A172V/+}$, and **(F)** *Mapk1*$^{A172V/A172V}$ mice in the hippocampus (HIPP), thalamus (THAL), and hypothalamus (HYP). Red arrows indicate differences in GFAP immunoreactivity in the hypothalamus. Scale bars are 1mm.

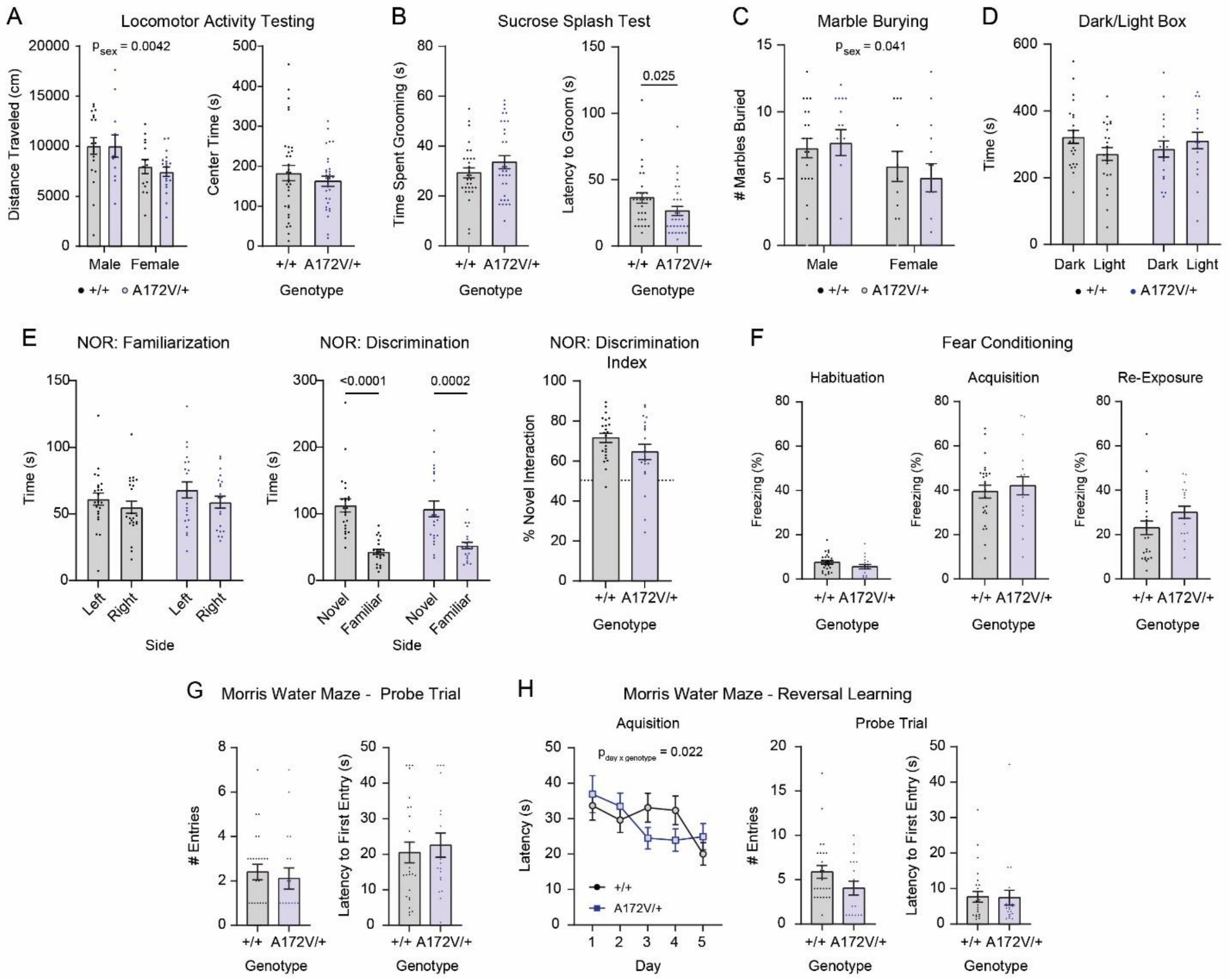

Figure S8. Behavioral testing of *Mapk1* mutant and control mice. (A) In a test of locomotor activity, there was a main effect of sex but not genotype (*left*; $n_{+/+}$ = 33, $n_{A172V/+}$ = 31; two-way ANOVA; $F_{1,60}$ = 0.098, $p_{sex\ x\ genotype}$ = 0.76; $F_{1,60}$ = 8.87, p_{sex} = 0.0042; $F_{1,60}$ = 0.12, $p_{genotype}$ = 0.73), and there were no changes in the time spent in the center of the arena (*right*; $n_{+/+}$ = 33, $n_{A172V/+}$ = 31; unpaired t-test with Welch's correction; $t_{55.23}$= 0.89, p = 0.38). **(B)** *Mapk1*$^{A172V/+}$ mice showed no significant changes in the amount of time spent grooming in the sucrose splash test (*left*; Mann-Whitney U-test; U($n_{+/+}$ = 30, $n_{A172V/+}$ = 31) = 408, p = 0.42) but had a shorter latency to initiate grooming behavior (*right*; Mann-Whitney U-test; U($n_{+/+}$ = 30, $n_{A172V/+}$ = 31) = 311.5, p = 0.025). **(C)** There was a significant main effect of sex but not genotype in the marble burying assay ($n_{+/+}$ = 33; $n_{A172V/+}$ = 31; two-way ANOVA; $F_{1,60}$ = 0.43, $p_{sex\ x\ genotype}$ = 0.52; $F_{1,60}$ = 4.35, p_{sex} = 0.041; $F_{1,60}$

= 0.055, $p_{genotype}$ = 0.82). **(D)** There were no effects of genotype or side in the dark/light box assay ($n_{+/+}$ = 24; $n_{A172V/+}$ = 18; two-way ANOVA; $F_{1,80}$ = 3.075, $p_{side\ x\ genotype}$ = 0.083; $F_{1,80}$ = 0.35, p_{side} = 0.56; $F_{1,80}$ = 0.0072, $p_{genotype}$ = 0.93). **(E)** No genotypic differences in object preference were observed during the familiarization phase of the novel object recognition (NOR) test (*left*; $n_{+/+}$ = 23; $n_{A172V/+}$ = 21; two-way repeated measures ANOVA; $F_{1,42}$ = 0.12, $p_{side\ x\ genotype}$ = 0.74; $F_{1,42}$ = 2.94, p_{side} = 0.094; $F_{1,42}$ = 1.021, $p_{genotype}$ = 0.32). During the discrimination phase, both genotypes interacted more with the novel object (*middle*; $n_{+/+}$ = 23; $n_{A172V/+}$ = 21; two-way ANOVA; $F_{1,42}$ = 0.69, $p_{object\ x\ genotype}$ = 0.41; $F_{1,42}$ = 49.37, p_{object} < 0.0001; $F_{1,42}$ = 0.065, $p_{genotype}$ = 0.80). There were no differences in the object discrimination index ((time spent with novel object – time spent with familiar objects) / total time interacting with both objects) between genotypes (*right*; $n_{+/+}$ = 23; $n_{A172V/+}$ = 21; unpaired t-test with Welch's correction; $t_{33.03}$= 1.58, p = 0.12). **(F)** During contextual fear conditioning, there were no significant differences between genotypes during the habituation phase before shocks were delivered (*left*; $n_{+/+}$ = 25, $n_{A172V/+}$ = 18; unpaired t-test; t_{41}= 1.55, p = 0.13), acquisition phase when shocks were presented (*middle*; $n_{+/+}$ = 25, $n_{A172V/+}$ = 18; unpaired t-test; t_{41}= 0.54, p = 0.59), or during context re-exposure 24-hours later (r*ight*; $n_{+/+}$ = 25, $n_{A172V/+}$ = 18; unpaired t-test; t_{41}= 1.62, p = 0.11). **(G)** During the probe trial of the Morris water maze (MWM), there were no significant differences in the number of entries into the target quadrant (*left*; Mann-Whitney U-test; $U(n_{+/+}$ = 25, $n_{A172V/+}$ = 18) = 194.5, p = 0.42) or latency to the first entry into the target quadrant (*right*; Mann-Whitney U-test; $U(n_{+/+}$ = 25, $n_{A172V/+}$ = 18) = 199, p = 0.53). **(H)** During the reversal learning acquisition phase of the MWM, there was a significant interaction between day and genotype (left; $n_{+/+}$ = 25, $n_{A172V/+}$ = 17; two-way ANOVA; $F_{4,160}$ = 2.95, $p_{day\ x\ genotype}$ = 0.022; $F_{3.73,149.1}$ = 5.26, p_{day} = 0.0007; $F_{1,40}$ = 0.024, $p_{genotype}$ = 0.88). *Post hoc* analysis determined that in wild-type mice, there were significant differences in latency on day 5 compared to their latency on days 1, 3, and 4 (Bonferroni *post hoc* test; p < 0.05), while no differences were observed in

Mapk1$^{A172V/+}$ mice (Bonferroni *post hoc* test, $p > 0.05$). During the reversal learning probe trial, there were no differences in entries into the target quadrant (*middle*; Mann-Whitney U-test; $U(n_{+/+} = 25, n_{A172V/+} = 15) = 120.5$, $p = 0.060$) and no difference in the latency to enter the target quadrant (right; Mann-Whitney U-test; $U(n_{+/+} = 25, n_{A172V/+} = 15) = 184$, $p = 0.93$). Data is presented as mean ± SEM.
