# Supplementary material for: Genetic activation of ERK2 recapitulates core neurodevelopmental features of Rasopathy syndromes in mice
Source: HGG Adv. 2026 Apr 28;7(3):100621. doi: 10.1016/j.xhgg.2026.100621 (PMC13234240; doi:10.1016/j.xhgg.2026.100621)
Supplement: Document S2. Article plus supplemental information [file mmc3.pdf]

HGG
Advances

ARTICLE

Genetic activation of ERK2 recapitulates core neurodevelopmental features of Rasopathy syndromes in mice

Kassidy E. Grover,[1] Zoe R. Cappel,[1] Avery M. Volz,[1] Evelin M. Cotella,[2] Kelly Smallwood,[3] Christine A. Berryhill,[4] Kimaya R. Raje,[5] Austen A. Fisher,[1] Mary Claire T. Casper,[1] Diana Nardini,[1] Tilat A. Rizvi,[1] Rosa M. Salazar,[1] Ashley Wooten,[1] Michael T. Williams,[5,6] Charles V. Vorhees,[5,6] Lindsey E. Romick,[7,8] Kenneth D. Greis,[8,9] Yueh-Chiang Hu,[6,10] Linde A. Miles,[1,6,8] Steven P. Angus,[4] Nancy Ratner,[1,6,8] Carlos E. Prada,[11,12] K. Nicole Weaver,[3,6,13,14] Ronald R. Waclaw,[1,6,8,10] and J. Elliott Robinson[1,6,8,15,*]

Summary

Germline pathogenic variants that activate the Ras/mitogen-activated protein kinase (MAPK) pathway cause neurodevelopmental disorders called Rasopathies. Because many affected proteins directly regulate Ras, causative mutations may alter other Ras-dependent pathways in addition to MAPK signaling. To better understand which Rasopathy sequelae result from hyperactivation of downstream MAPKs, we engineered mice with a gain-of-function mutation in the terminal MAPK gene *Mapk1*, which encodes ERK2 and is associated with the recently described genetic syndrome *MAPK1*-related Rasopathy (MRR). *Mapk1* mutant mice successfully modeled key aspects of the human MRR phenotype, including small stature, facial dysmorphism, and impaired cognitive function. Importantly, they recapitulated phenotypes identified in Rasopathy models with upstream Ras activation, such as neurofibromatosis type 1 (NF1): oligodendrocyte lineage defects, reactive astrogliosis, memory deficits, and hypersensitivity to sensory stimuli. These findings emphasize the importance of downstream MAPK signaling in the pathophysiology of neurocognitive symptoms observed in Rasopathy syndromes.

Introduction

The Ras/mitogen-activated protein kinase (MAPK) pathway is a biochemical signaling cascade that regulates several basic cellular processes, including growth, differentiation, and responses to stress.[1–5] In this pathway, extracellular ligands (e.g., hormones, cytokines, and growth factors) alter cell states by binding cell surface receptors, which activate the Ras family of small GTPases by promoting the exchange of guanosine diphosphate (GDP) for guanosine triphosphate (GTP). Once active, GTP-bound Ras initiates the sequential phosphorylation of a series of serine/threonine protein kinases (Raf, MEK, and extracellular signal-regulated kinase [ERK]). ERK is the terminal MAPK and influences cellular function by directly phosphorylating cytosolic targets or traveling to the nucleus where it can regulate gene expression.[3,4] While somatic mutations activating the MAPK pathway are prevalent in cancers, germline variants are associated with a family of developmental disorders called Rasopathies.[6] Rasopathies include neurofibromatosis type 1 (NF1 [MIM: 162200]), Noonan syndrome (NS [MIM: 163950]), NS with multiple lentigines (NSML [MIM: 151100]), Costello syndrome (CS [MIM: 218040]), and cardiofaciocutaneous syndrome (CFC [MIM: 115150]). These Rasopathy syndromes have unique and shared clinical features, such as developmental delay, craniofacial abnormalities, congenital heart defects, cognitive impairment, and an increased risk of malignancy.[6–8]

NS is the most common Rasopathy and has an incidence of approximately 1 in 1,000–2,500 live births.[9] In addition to manifestations listed above, individuals with NS may experience short stature, autism spectrum disorder (ASD [MIM: 209850]), attention-deficit/hyperactivity

[1]Division of Experimental Hematology and Cancer Biology, Department of Pediatrics, Cincinnati Children's Hospital Medical Center, Cincinnati, OH 45229, USA; [2]Department of Functional and Systems Neuroscience, Instituto Mercedes y Martin Ferreyra INIMEC-CONICET, Universidad Nacional de Córdoba, Córdoba, Argentina; [3]The Heart Institute, Cincinnati Children's Hospital Medical Center, Cincinnati, OH 45229, USA; [4]Department of Pediatrics, Indiana University School of Medicine, Indianapolis, IN 46202, USA; [5]Department of Neurology, Cincinnati Children's Hospital Medical Center, Cincinnati, OH 45229, USA; [6]Department of Pediatrics, University of Cincinnati College of Medicine, Cincinnati, OH 45229, USA; [7]Division of Pathology and Laboratory Medicine, University of Cincinnati College of Medicine, Cincinnati, OH 45229, USA; [8]University of Cincinnati Cancer Center, University of Cincinnati College of Medicine, Cincinnati, OH 45229, USA; [9]Department of Cancer Biology, University of Cincinnati College of Medicine, Cincinnati, OH 45229, USA; [10]Division of Developmental Biology, Cincinnati Children's Hospital Medical Center, Cincinnati, OH 45229, USA; [11]Edwards Family Division of Genetics and Rare Diseases, Ann & Robert H. Lurie Children's Hospital of Chicago, Chicago, IL 60611, USA; [12]Department of Pediatrics, Feinberg School of Medicine, Northwestern University, Chicago, IL 60611, USA; [13]Division of Human Genetics, Cincinnati Children's Hospital Medical Center, Cincinnati, OH 45229, USA
[14]All editorial responsibility for this paper was handled by an associate editor of the journal.
[15]Lead contact
*Correspondence: elliott.robinson@cchmc.org
https://doi.org/10.1016/j.xhgg.2026.100621.
© 2026 The Author(s). Published by Elsevier Inc. on behalf of American Society of Human Genetics.
This is an open access article under the CC BY license (http://creativecommons.org/licenses/by/4.0/).

disorder (ADHD [MIM: 143465]),[10–13] gastrointestinal problems,[14] etc. The genetic etiology of NS is heterogeneous and most commonly results from mutations in *PTPN11* (MIM: 176876), *SOS1* (MIM: 182530), *KRAS* (MIM: 190070), and *RIT1* (MIM: 609591) genes.[9] Of these, *PTPN11* mutations are most prevalent, occurring in approximately 50% of cases.[15,16] Like individuals with NS, mice with gain-of-function mutations in *Ptpn11*, *Kras*, and *Rit1* genes experience cardiac defects, small size, craniofacial and hematological abnormalities, and cognitive dysfunction.[17–20] Because these mutations affect signaling upstream or at the level of the Ras GTPase, phenotypes may be caused by aberrant activation of downstream MAPKs or other Ras-regulated proteins, such as phosphoinositide 3-kinase, Rac1, and Ral.[11,21,22] Therefore, the development of transgenic mice with gain-of-function mutations in terminal MAPKs would clarify the role of this signaling pathway in the pathophysiology of Rasopathy syndromes.

Recently, an autosomal dominant neurodevelopmental syndrome on the Rasopathy clinical spectrum with NS-like features was described in seven individuals with *de novo* missense variants in the *MAPK1* (MIM: 176948) gene,[23] which encodes the terminal MAPK ERK2. These individuals with *MAPK1*-related Rasopathy (MRR) exhibited craniofacial dysmorphism, intellectual disability, developmental delay, behavioral problems, small stature, hypotonia, and electroencephalogram (EEG) abnormalities. Identified *MAPK1* mutations were classified as gain-of-function in functional studies since they enhanced ERK2 phosphorylation and nuclear translocation following growth-factor stimulation *in vitro*.[23] However, pathogenic *MAPK1* variants have never been modeled in laboratory mammals, which would facilitate mechanistic studies to elucidate the pathophysiology of MRR and determine whether ERK gain-of-function genocopies features observed in Rasopathy model mice with upstream Ras activation. To address this need, we generated a mouse model of MRR to identify the physiological, anatomical, and neurodevelopmental sequelae of enhanced ERK2 signaling *in vivo* and explore the role of downstream MAPK proteins in Rasopathy phenotypes.

Material and methods

Mice

All experiments were conducted following the Institutional Animal Care and Use Committee (IACUC) of Cincinnati Children's Hospital Medical Center (CCHMC) under protocols 2023-0044 and 2025-0007 and the National Institute of Health Guidelines for the Care and Use of Laboratory Animals. *Mapk1*^{A172V} mice were generated by the CCHMC Transgenic Animal and Genome Editing Core using CRISPR technology to introduce the Ala172Val mutation plus a Sal1 site for genotyping. The correctly edited mutant allele founder mice (confirmed via Sanger sequencing) were bred to CD1 mice and maintained on this background. Heterozygous mutant mice (*Mapk1*$^{A172V/+}$) were bred to produce wild-type, heterozygous, and homozygous mutant offspring. Mice were PCR genotyped with the following primers: Mapk1-5′-GTTTTCCTTGTTACTGATACTGCC-3′ and Mapk1-3′-ACTGAAGATGGTGACTCCTAAGC-5′ to amplify a 324-bp product, which was subsequently digested with Sal1 restriction enzyme to produce 200- and 124-bp products only in mutant mice. All mice were group housed by sex (up to four per cage) in standard shoebox cages on a 14/10 light/dark cycle with *ad libitum* access to food (LabDiet, catalog no. [Cat#] 5010) and water. Mice were weighed at postnatal day (PD) 9, 21, 30, 44, 58, 72, 86, 100, and 150. Behavioral experiments were conducted after mice reached adulthood (after 12 weeks of age). Marble burying, sucrose splash test, dark-light testing, novel-object recognition, open-field testing, and the looming-stimulus assay were performed in the Robinson Lab at CCHMC. All other behavioral tests were performed at the Animal Behavior Core at CCHMC. Male and female mice were used in all experiments.

Behavioral assays

Marble burying

The protocol used was adapted from Angoa-Pérez et al.[24] Mice were introduced to a cage filled halfway with clean, leveled corncob bedding. In each cage, 15 opaque black marbles were arranged in an approximately equidistant 3 × 5 grid. After 30 min, the number of marbles buried was recorded. A marble was considered buried if at least 2/3 of the marble was submerged. The mean number of marbles buried was calculated for each genotype.

Sucrose splash test

The protocol used was adapted from Frisbee et al.[25] Mice were sprayed with 10% sucrose solution on the upper neck between the ears and allowed to roam in a clean, empty cage for 5 min. Sessions were recorded using a Basler acA2040-120um camera with an Edmunds Optics TECHSPEC 6mm C Series fixed-focal-length lens, and experimenters examined behavior every 5 s. Total grooming and latency to groom were calculated for each mouse.

Open field test

Recordings of each session were taken through a camera (Basler acA2040-120um and Edmunds Optics TECHSPEC 6mm C Series fixed-focal-length lens). Mice were placed in the corner of a large plastic 55 × 45-cm storage container facing the wall and allowed to explore for 30 min. Ethovision XT (Noldus Information Technology, Wageningen, the Netherlands) was used to determine the distance traveled and the time spent in the center of the arena.

Novel object recognition

The protocol used was adapted from Bevins and Besheer.[26] Specifically, in 55 × 45-cm arenas, two objects were placed equally spaced apart and fixed to the bottom of the container with Gorilla mounting putty. The objects were toy building blocks that were identical in size and color. Mice were allowed 10 min to explore the arena during the familiarization phase before being returned to their home cage. One hour later, the mice were returned to the same arena, but one of the objects was changed to a novel object, which was different in size and color from the original. The left-right location of the novel object was counterbalanced between mice. Again, mice were allowed to explore the objects for 10 min before being removed and placed back into their home cage. Videos for familiarization and novel object exploration were obtained with a Basler acA2040-120um camera with an

Edmunds Optics TECHSPEC 6mm C Series fixed focal length lens, and mouse position was tracked using Ethovision XT software. Interaction with an object was determined when the nose of the mouse entered a region of interest that measured a circle with a 12-cm diameter around the object. The time of interaction was measured, and the discrimination ratio was calculated (time spent with novel object – time spent with familiar objects/total time interacting with both objects).

Dark-light box

The behavioral apparatus was a 37 × 20-cm arena that was one-third covered by an opaque, infrared (IR) light-lucent shelter with an opening for mouse entry. The entire arena was exposed to bright light. Mice could move freely between dark and light sides. Behavior was recorded and tracked using Ethovision XT. An IR light backlit panel was used under the arena to allow the software to detect the mouse inside the shelter. Mice were placed in the lit side of the arena facing toward the wall and away from the shelter. The latency to cross into the shelter, time spent in the shelter, time spent in the open arena, and crossings between the shelter and exposed arena were measured over a 10-min period.

Looming

The looming stimulus assay was performed as described.[27,28] The clear acrylic arena measured 20.3 cm (width) × 61 cm (length) × 40.6 cm (height) and contained an IR light-lucent shelter at one side (the opening was the width of the arena) to serve as the escape location. A 9-cm Petri dish was placed at the opposite end of the apparatus to encourage exploration of the "threat zone" in the open arena. A 15.6-inch LCD monitor was placed above the threat zone to present a train of five looming stimuli (expanding black disks with a maximum diameter of 19.5 cm encompassing 27° of visual angle) when the mouse entered the threat zone. The entire arena and monitor were placed inside a light- and sound-attenuating enclosure that could be closed during testing to minimize environmental stimuli during performance of the assay. An IR backlight (880-nm backlit collimated backlight, Advanced Illumination) was used to allow mouse tracking in the light-attenuated chamber. To avoid overheating from the backlight, the arena was elevated on four small clear pedestals that were inaccessible to the mice, allowing airflow and space between the light and the floor of the arena. Mouse behavior was recorded using a Pylon camera with an Edmunds Optics TECHSPEC 6mm C Series fixed-focal-length lens positioned above the arena to allow for a full field of view. Ethovision XT was used for real-time tracking and was synchronized with Bonsai[29] to trigger the looming stimulus on the LCD monitor when the mouse entered the threat zone after a 10-min habituation period. After the train of looming disks was presented, mice were recorded for 3 min before being returned to their home cage. Mice were excluded from analysis if they never entered the threat zone and thus never triggered a looming stimulus after 20 min.

Morris water maze

The arena was a pool that measured 150 cm in diameter and 51 cm high and that contained a platform that was submerged 1–1.5 cm below the water level. The water was kept at ~21°C. Mice were tracked using Anymaze software (Stoelting Company, Wood Dale, IL). There were three phases of the experiment: training, acquisition, and reversal learning. The starting quadrant was rotated in acquisition and reversal phases so no mouse started in the same quadrant on consecutive trials. Distance, speed, latency, and path efficiency were recorded for each phase; however, because of the coat color of the mice, only latency was obtained. Training occurred on day 1 of testing. During this phase, curtains were drawn to cover external cues in the room. A 10-cm-diameter platform with an orange ball was mounted to the platform. There was a 90-s time limit per trial with an intertrial interval (ITI) of at least 10 min. If a mouse failed to find the platform at least twice within six trials, it was retested 4–6 h later. Mice that found the platform moved on to the next phase of the experiment, while mice that failed to find the platform when retested were excluded (4 *Mapk1*$^{A172V/+}$ mice). The acquisition phase occurred on days 2–7 with 5 days of learning and one memory (probe) trial on day 7. There were four trials each day with a 90-s limit per trial and an ITI of at least 10 min. During this phase, curtains were open to expose distal cues on the walls that mice could use to navigate the pool. The 10-cm-diameter camouflaged platform was submerged 1–1.5 cm below the water surface. On the final day, during the probe trial, the platform was removed from the water bath to measure spatial memory for the platform on previous days. The final phase was reversal testing, which measures cognitive flexibility and was performed over 6 days of testing. The phase was identical to the acquisition phase, but a 7-cm-diameter platform was placed into the opposite quadrant during the 5 days of learning. The platform was then removed during the probe trial on day 6.

Contextual fear conditioning

The experiment was conducted using the San Diego Instruments Freeze Monitor system (San Diego Instruments, San Diego, CA). The system contained a conditioning chamber with a metal-bar floor for foot-shock delivery (1.0 mA) and a photobeam array to record the start and end of freezing episodes. The test took place over 2 days. The first day was the habituation phase, which consisted of 5 min of acclimation followed by nine intervals of shock delivered during the last 2 s of the 30-s ITI. This lasted 10 min. The second day was to test the mouse's contextual memory. The mice were placed in the same chamber as day 1, but no foot shocks were delivered. This phase was 6 min long. Freezing behavior was measured in both phases.

24-h home-cage monitoring

The test was performed after P60 for two consecutive 24-h recording periods using the HomeCage Photobeam Activity System (San Diego Instruments). Mice were individually housed in a static cage within the apparatus that contained a Napa Nectar gel and food pellets. After testing, mice returned to their home cage with their cage mates. Locomotor activity was measured as beam breaks per hour over 48 h of testing.

Pre-pulse inhibition

The test was performed in an SR-LAB apparatus (San Diego Instruments). Mice were placed in an acrylic cylindrical holder attached to an acrylic base plate with a piezoelectric accelerometer transducer to detect movement. During the session, a fan was on, creating background ambient noise. Mice were given a 5-min acclimation period before testing began. Pulses were 20-ms mixed-frequency white-noise bursts at 120-dB intensity. Prepulses were 59, 70, or 80 dB and were presented in counterbalanced order in a 20-trial block Latin square. Peak response amplitude (Vmax) was recorded.

MEK inhibitor in milk treatment

MEK inhibitor (MEKi) PD0325901 (Selleck Chemicals, Cat# 1036) was administered to lactating dams as described.[30] In brief, MEKi was dissolved in 0.5% methylcellulose and 0.2% Tween-80, which was used as the vehicle control. Cages were randomly selected for MEKi or vehicle treatment before pups were born,

and MEKi (5mg/kg, which delivers ~1%–2% of the maternal dose to pups via the milk[30]) or vehicle was delivered to lactating dams via oral gavage once a day from PD0.5 until PD21. Mice were euthanized for genotyping at PD21.

Blood collection and analysis

Trunk blood was collected from PD14-old mice into MiniCollect tubes with K2EDTA (Greiner Bio-One, Monroe, NC) following rapid decapitation. Samples were analyzed using a Heksa Element HT5 hematology analyzer to obtain a complete blood count with five-part differential (Antech Diagnostics, Vermont, VIC, Australia). Samples were analyzed using a one-way ANOVA to determine differences between all three genotypes (*Mapk1*$^{+/+}$, *Mapk1*$^{A172V/+}$, and *Mapk1*$^{A172V/A172V}$ mice).

Tissue extraction and analysis

Brain protein extraction

Brains were extracted from ED16.5, PD15, and PD100 mice following rapid decapitation. The cerebellum and hindbrain were removed and the forebrain was collected. One-half of the forebrain was homogenized. RIPA Lysis Buffer (Thermo Fisher Scientific) and Halt Protease and Phosphatase Inhibitor Cocktail (Thermo Fisher Scientific) were added prior to homogenization. Then the tissue was agitated for 1 h at 4°C and centrifuged at 14,000 × g for 14 min at 4°C. The supernatant was aliquoted and stored at −80°C until use. Protein was quantified using protocol 5.2 Microplate Assay Protocol of Detergent Compatible (DC) Protein Assay Instruction Manual (Bio-Rad) and used for western blots.

Western blot

Cell lysates were heated for 5 min at 95°C with 6X Laemmli SDS sample buffer, reducing (Thermo Fisher Scientific). Samples were loaded onto a 10% Mini-PROTEAN TGX Gel (Bio-Rad) and run at 150 V until separated. Protein was transferred to a polyvinylidene difluoride (PVDF) membrane (Bio-Rad) for 45 min at 100V. The PVDF membrane was blocked in Intercept Blocking Buffer (LI-COR) for 1 h at room temperature followed by incubation in primary antibody (pERK, Cell Signaling Technology, #4370, 1:1,000; ERK, Cell Signaling Technology, #4696, 1:1,000, or Cell Signaling Technology, #4695, 1:1,000; and β-actin, Cell Signaling Technology, #3700, 1:1,000) diluted in Intercept Antibody Diluent (LI-COR) at 4°C overnight. The following day, the membrane was washed three times for 5 min each with Tris-buffered saline (TBS)-T before being incubated with the secondary antibody (IRDye 680RD Goat anti-Mouse IgG, LI-COR, #926-68070, 1:10,000 or 1:20,000; IRDye 800CW Donkey anti-Rabbit, LI-COR, #926-32213, 1:10,000 or 1:20,000) that was diluted in Intercept Antibody Diluent (LI-COR) for 1 h at room temperature. The blot was then washed in TBS-T three times for 10 min followed by one wash for 10 min in TBS. Signals were visualized on the Odyssey CLx (LI-COR) using ImageStudio Ver 5.2 (LI-COR). Data were analyzed through Fiji and GraphPad Prism 10.1.2. If applicable, the PVDF membrane was stripped in Restore Western Blot Stripping Buffer (Thermo Fisher Scientific), followed by 30 min at room temperature in Intercept Blocking Buffer (LI-COR) before being incubated in the new primary antibody overnight at 4°C and repeating the second day of the protocol.

Phosphoproteomics

Phosphoproteomic quantification via mass spectrometry was performed at the University of Cincinnati (UC) Proteomics Laboratory at the UC College of Medicine using forebrain samples from PD15 mice (4 *Mapk1*$^{+/+}$ mice and four *Mapk1*$^{A172V/+}$ mice, two male and two female samples per genotype). The brain tissue was frozen using liquid nitrogen directly in Kimble Kontes homogenizer tubes (item# 749520-0000), broken up within the tube with a small spatula, and homogenized in urea lysis buffer, which contained 9 M urea, 20 mM HEPES, 1 mM b-glycero-phosphate, 2.5 mM sodium pyrophosphate, and 1 mM activated sodium orthovanadate. The samples were adjusted to 1-mL total volume and probe sonicated at 15 W for 15 s for three cycles. Unsolubilized material was pelleted via centrifugation, and samples were placed in new tubes. 3-mg aliquots were removed from each sample, and the volume was adjusted up to 1 mL with undiluted urea lysis buffer. The samples were digested with modified trypsin (Worthington Biochemical, Cat# LS003740) overnight at 37°C. The digested peptides were purified and concentrated by passing them over a Sep-Pak C18 cartridge (Waters, Cat# WAT051910), dried in a SpeedVac, and resuspended in binding/equilibration buffer from the Thermo Fisher High-Select TiO_2 Phosphopeptide Enrichment Kit (Cat# A39223), which was used for peptide enrichment according to the manufacturer's instructions. Eluted samples were dried in a SpeedVac and resuspended in 0.1% formic acid. 10% of each enriched sample was analyzed by nanoscale liquid chromatography coupled with tandem mass spectrometry (Orbitrap Eclipse).

All downstream analyses of the mass spectrometry data were performed using R v.4.5.0. Phosphosites were filtered for a high false discovery rate (FDR) and a site localization score (ptmRS module) greater than 0.75 and unambiguous. The *PhosR* package was used for filtering, imputation, and scaling. Phosphopeptides with greater than 25% missing values across all samples were filtered out. For remaining phosphopeptides, site- and condition-specific imputation was performed if at least 50% of a given phosphopeptide was present in the defined condition (*Mapk1*$^{+/+}$ and *Mapk1*$^{A172V/+}$). Differentially abundant phosphopeptides were identified using a t test ($p < 0.05$) and a fold-change cutoff of |1.5|. The following packages were used for subsequent downstream analysis: *ReactomePA* (reactome pathway analysis), *ggplot2* (bar graphs and volcano plot), *ggpubr* (statistics on bar graphs), and *ssGSEA2* (post-translational modification-signature enrichment analysis [PTM-SEA]). For the PTM-SEA analysis, the ptm.sig.db.all.uniprot.mouse.v2.0.0 database was used, with all other parameters set to their default states.

NMR metabolomics

The nuclear magnetic resonance (NMR)-based metabolomics experiments were performed at CCHMC Translational Metabolomics Facility (RRID: SCR_022636). Liver tissue samples were extracted using modified Bligh and Dyer methods[31–33] to obtain polar metabolites. The extraction-solvent volumes were determined based on the average water content of mouse liver tissue (72.8%). Plasma samples were processed according to the published protocol.[34] Briefly, the plasma samples were filtered through pre-washed 3-kDa spin filters and the filtrate was mixed with NMR buffer up to the final volume of 220 μL. NMR data collection and processing were performed with a Bruker Avance III HD 600-MHz spectrometer using Topspin 3.6 software (Bruker Analytik, Rheinstetten, Germany) as previously described.[35] A total of 49 liver and 40 plasma metabolites were assigned based on the chemical shifts on one-dimensional nuclear overhauser effect spectroscopy (1D 1H-NOESY), two-dimensional total correlation spectroscopy (2D-TOCSY), and -heteronuclear single quantum coherence (HSQC) NMR experiments with reference spectra found in databases Human Metabolome Database (HMDB),[36] and Chenomx NMR Suite profiling software

(Chenomx version 8.1). The metabolites were quantified by Chenomx software based on the internal standard, trimethylsilylpropanoic acid (TMSP), and were normalized to tissue weights or plasma volume prior to statistical analysis by R studio and MetaboAnalyst.[37] Log transformation and mean centering data scaling were applied prior to multivariate analysis in MetaboAnalyst.

Immunohistochemistry

Tissue collection and cryostat sectioning

Mice were transcardially perfused with PBS followed by 4% paraformaldehyde (PFA). The brain was extracted into PFA at 4°C overnight. For ED 18.5 and PD 14 histology, mice were euthanized and brains were dissected and drop fixed in 4% PFA. The following day, the brains were transferred to 20% sucrose for cryoprotection. Brains were mounted in Tissue-Tek O.C.T. Compound (Thermo Fisher Scientific), sectioned in the cryostat at −20°C into 12-μm serial sections, and mounted on slides. Slides were stored at −80°C until use.

Immunohistochemistry

In Coplin jars, warmed citrate buffer was poured over slides for antigen retrieval for a maximum of 5 min. Slides were then bleached with 0.33% H_2O_2 for 10 min and washed three times for 5 min each with 1× Potassium-based Phosphate Buffered Saline (KPBS). Slides were then incubated in primary antibodies overnight in a humid chamber (Nkx2.2, Abcam, #ab191077, 1:1,000; GFAP, Agilent, #Z0334, 1:2,000; PDGFRα, R&D Systems, #AF1062, 1:1,000; and Myrf, ABclonal, #A16355, 1:1,000). The following day, slides were washed in 1× KPBS for 5 min three times. A biotinylated secondary antibody was added (Biotin-SP [long spacer] AffiniPure Donkey Anti-Goat IgG [H + L], Jackson ImmunoResearch, #705-065-147, 1:500; Biotin-SP [long spacer] AffiniPure Donkey Anti-Rabbit IgG [H + L], Jackson Immuno, #711-065-152, 1:500) for 2 h at room temperature in a humid chamber. Slides were washed three times for 5 min each in 1× KPBS and incubated in Avidin-Biotin Complex (ABC) (Vector Laboratories) for 1 h in a humid chamber at room temperature. Slides were washed again three times for 5 min in 1× KPBS. Diaminobenzidine (DAB) was then added to the slides in a humid chamber for 5 min, then washed in 1× KPBS three times for 5 min. Slides were left to dry overnight before being coverslipped. To coverslip, slides are rehydrated for 5 min before dehydrated in a series of ETOH washes (70%, 95%, and 100%) for 5 min each. Slides are then put into two washes of xylene for 5 min each and cover slipped with DPX mountant medium (Sigma-Aldrich). CellProfiler (version 4.2.8) was used to quantify the images. *In situ* hybridization for *Etv5* was performed as previously described.[38]

Heart immunohistochemistry

Histological analysis was conducted on formalin-fixed tissues, paraffin embedded, sectioned at 5–8 μm, and stained either with hematoxylin and eosin (H&E) or with Russell-Movat pentachrome stain.[39] Whole-heart H&E-stained sections were viewed on a Zeiss Discovery V8 stereomicroscope and imaged with an Axiocam 820 camera. Valve pentachrome-stained sections were viewed on a Zeiss Axio Imager Z1 with an Axiocam 305 camera. Images were viewed and measured using Zeiss ZEN Blue software. The myocardial thickness for each sample was measured at three positions for each ventricle: the dorsal, lateral, and ventral surface. Equal numbers of sections were selected at random for each set of comparisons.

Skull skeletal preparations

Heads from PD7 *Mapk1*$^{+/+}$ and *Mapk1*$^{A172V/+}$ pups were deskinned and fixed in ethanol for 2 days. Skulls were then washed in Alcian blue dye (Sigma-Aldrich) overnight at room temperature to stain for cartilage. Skulls were rinsed with ethanol for 2 days and cleared with 1% KOH for 6 h until they were clear. After clearing, skulls were counterstained with Alizarian red stain (Sigma-Aldrich) for 24 h to visualize bone. Finally, skulls were cleared with fresh 20% glycerol/1% KOH solution daily until the clearing was complete and stored in a 50% glycerol/50% ethanol solution. Skulls were imaged using a Zeiss Discovery V8 stereomicroscope with an Axiocam 820 color camera and ZEN Blue software.

Statistical analysis

All statistical tests were conducted using R or GraphPad Prism version 10.5 (GraphPad). Sex was considered as a variable in a two-way or three-way ANOVA when appropriate. If no sex differences were found, the statistical testing was performed with the sexes combined. Parametric tests were conducted unless the data were found to be nonparametric using the D'Agostino-Pearson test for normality. For data that had small sample sizes, we used the Shapiro-Wilk test of normality instead. A Welch's correction was used for t tests if sample variances were significantly different. For body-mass data, a mixed-effects model with the Geisser-Greenhouse correction was used instead of a repeated-measures ANOVA to account for missing values. The Bonferroni correction was used for *post hoc* testing after an ANOVA if any variable had three or more levels. A Dunn's *post hoc* test was used for Kruskal-Wallis tests, and the Fisher's least significant difference (LSD) test was used for all other *post hoc* tests. Inheritance data were calculated using a Chi-squared test of independence. Survival data were calculated using the log rank test and plotted using a Kaplan-Meier plot. For correlations, we used Pearson's correlation coefficient to determine the strength of a relationship between two variables, and linear regressions were run to generate regression lines on the graphs. All source data and results of the statistical testing procedures are provided in the source data and statistical analysis file.

Results

Development of a mouse model of MAPK1-related Rasopathy

MRR is a recently described Rasopathy syndrome without an established mouse model to facilitate mechanistic studies and aid treatment development. To address this gap and determine the neurodevelopmental effects of increased ERK2 activity, we used CRISPR to generate mouse avatars with *MAPK1* mutations reported in a clinical cohort.[23] The first genome-edited mouse (*Mapk1*^{P321R} mice) contained a missense mutation that is equivalent to a human variant (c.968C>G [p.P323R]) that causes a proline-to-arginine amino acid substitution at position 323, which is near the D-site regulation site (DRS) and reduces the ability of regulatory factors (e.g., DUSP6) to dephosphorylate ERK2. The second mouse contained an alanine-to-valine substitution at position 172 (Figure 1A). This missense mutation is equivalent to the human

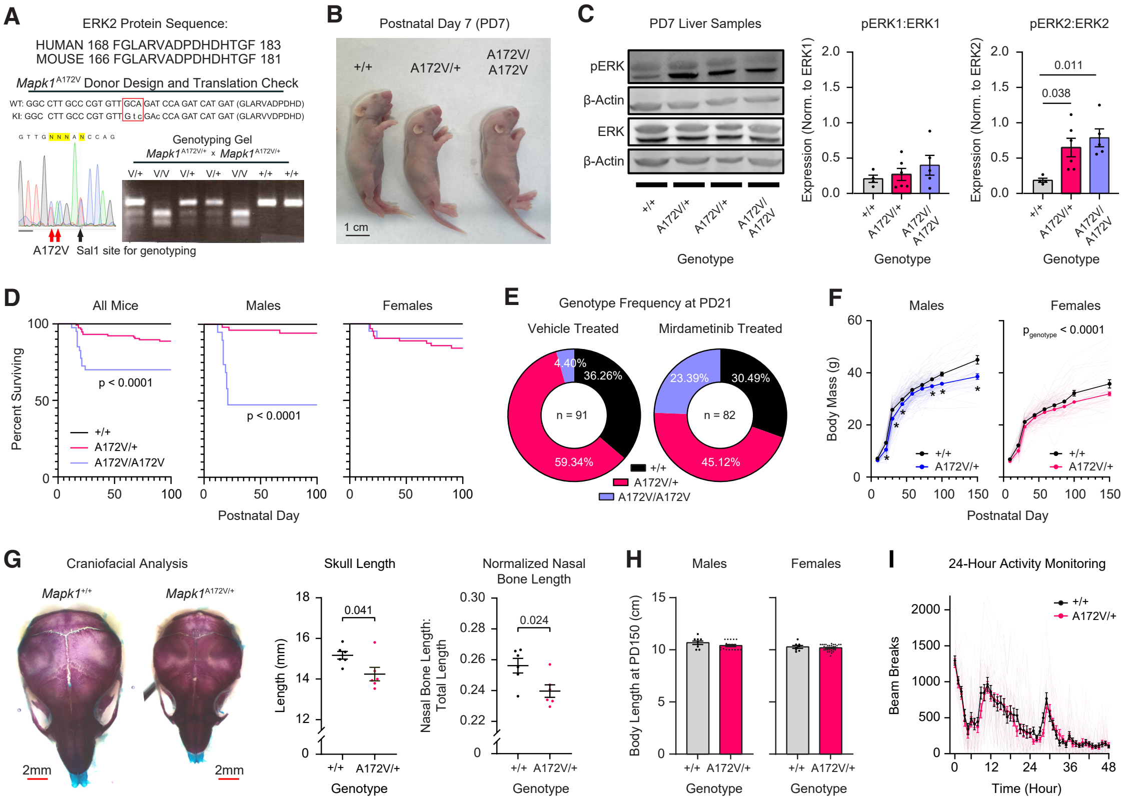

Figure 1. Development and characterization of a mouse model of *MAPK1*-related Rasopathy

(A) *Mapk1*^{A172V} transgenic mouse design showing homology between mouse and human sequences (top), sequencing confirmation of gene edit (bottom left), and a representative genotyping gel (bottom right).
(B) *Mapk1*$^{+/+}$, *Mapk1*$^{A172V/+}$, and *Mapk1*$^{A172V/A172V}$ pups at PD7 (scale bar: 1 cm).
(C) Representative western blot images of liver samples from *Mapk1* mutant and control mice (left). There were no differences in the pERK1:ERK1 ratio (middle) between genotypes ($n_{+/+} = 4$, $n_{A172V/+} = 6$, $n_{A172V/A172V} = 5$. One-way ANOVA: $F_{2,12} = 0.84$, $p = 0.45$). pERK2:ERK2 (right) was significantly increased (one-way ANOVA: $F_{2,12} = 6.17$, $p = 0.014$) in *Mapk1*$^{A172V/+}$ (Bonferroni *post hoc* test; $p = 0.038$) and *Mapk1*$^{A172V/A172V}$ mice ($p = 0.011$) relative to *Mapk1*$^{+/+}$ controls.
(D) *Mapk1*^{A172V} carriers exhibited significant early postnatal mortality (left; $n_{+/+} = 61$, $n_{A172V/+} = 115$, $n_{A172V/A172V} = 40$; log rank test; $X^2(2, N = 216) = 23.66$, $p < 0.0001$) that was largest in male *Mapk1*$^{A172V/A172V}$ mice (middle; $n_{+/+} = 33$, $n_{A172V/+} = 52$, $n_{A172V/A172V} = 19$; log rank test; $X^2(2, N = 104) = 42.40$, $p < 0.0001$). No differences in mortality were observed in female *Mapk1* mutants (right; $n_{+/+} = 28$, $n_{A172V/+} = 63$, $n_{A172V/A172V} = 21$; log rank test; $X^2(2, N = 112) = 4.914$, $p = 0.086$).
(E) Low incidence of *Mapk1*$^{A172V/A172V}$ mice at PD21 was observed in vehicle-treated cages (left), deviating from Mendelian ratios (36.26% *Mapk1*$^{+/+}$, 59.34% *Mapk1*$^{A172V/+}$, and 4.40% *Mapk1*$^{A172V/A172V}$. Chi-squared test: $X^2(2, N = 91) = 15.79$, $p = 0.0004$). Mirdametinib treatment (right) restored Mendelian ratios in *Mapk1* mutants (30.49% *Mapk1*$^{+/+}$, 45.12% *Mapk1*$^{A172V/+}$, and 24.39% *Mapk1*$^{A172V/A172V}$. Chi-squared test: $X^2(2, N = 82) = 0.57$, $p = 0.75$).
(F) Male (left) *Mapk1*$^{A172V/+}$ mice have reduced body mass (mixed-effects analysis; $F_{8,875} = 5.98$, $p_{day \times genotype}$ <0.0001; $F_{3.57,390.0} = 1918$, p_{day} <0.0001; $F_{1,147} = 31.45$, $p_{genotype} < 0.0001$) specifically at PDs 9, 21, 30, 44, 86, 100, and 150 (Bonferroni *post hoc* test; asterisk denotes $p < 0.05$). Female (right) *Mapk1*$^{A172V/+}$ mice had lower mass than *Mapk1*$^{+/+}$ littermates (mixed-effects analysis; $F_{8,810} = 1.80$, $p_{genotype \times day} = 0.074$; $F_{1,138} = 16.20$, $p_{genotype} < 0.0001$; $F_{3.42,345.9} = 841.6$, $p_{day} < 0.0001$).
(G) Stained skulls from *Mapk1*$^{A172V/+}$ and *Mapk1*$^{+/+}$ mice at PD7 (left; scale bar: 2 mm). *Mapk1*$^{A172V/+}$ had reduced skull length (middle; Mann-Whitney U test; $U(n_{+/+} = 6, n_{A172V/+} = 6) = 5$, $p = 0.041$) and normalized nasal bone length (right; $n_{+/+} = 6$; $n_{A172V/+} = 6$; unpaired *t* test; $t_{10} = 2.65$, $p = 0.024$).
(H) No genotypic differences in body length were observed in male (left; $n_{+/+} = 9$; $n_{A172V/+} = 19$; *t* test, $t_{26} = 1.50$, $p = 0.15$) and female (right; $n_{+/+} = 8$; $n_{A172V/+} = 30$; *t* test, $t_{36} = 0.63$, $p = 0.53$) at PD150.
(I) No differences in home-cage locomotor activity was observed between genotypes (two-way repeated-measures ANOVA; $F_{47,2115} = 0.74$, $p_{time \times genotype} = 0.90$; $F_{1,45} = 0.15$, $p_{genotype} = 0.52$), but the test did reveal a significant main effect of time ($n_{+/+} = 24$; $n_{A172V/+} = 23$; two-way repeated-measures ANOVA; $F_{12.87,579.2} = 45.52$, $p_{time} < 0.0001$) over the 48-h period. Data are presented as mean ± standard error of the mean (SEM).

MAPK1 A174V variant (c.521C>T [p.A174V]) near the ERK2 activation segment that changes conformation when the kinase is phosphorylated at threonine 185 and tyrosine 187.[23,40] We were able to obtain founders containing either a *Mapk1*^{P321R} or *Mapk1*^{A172V} allele; however, only *Mapk1*^{A172V} carriers successfully transmitted the mutation via the germline and produced viable offspring (Figure 1B). Selective ERK2 activation (pERK2 and the pERK2:total ERK2 ratio) was confirmed in PD7 liver samples (Figure 1C; Figure S1) without a change in phosphorylated ERK1 or total ERK1/2 levels (Figure S1). Of 476 mice genotyped before weaning, there was a trend toward

reduced prevalence of mice carrying two copies of the mutant allele, but it did not deviate from Mendelian ratios (32.05% *Mapk1*$^{+/+}$, 49.42% *Mapk1*$^{A172V/+}$, and 18.53% *Mapk1*$^{A172V/A172}$; Chi-squared test; $X^2(2, N = 476) = 0.78$, $p = 0.68$). In some mice, the *Mapk1*^{A172V} variant was associated with failure to thrive (body mass <4.0 g), resulting in gene-dose-dependent postnatal mortality by PD21 (Figure 1D). This was most severe in *Mapk1*$^{A172V/A172V}$ male mice, 52.63% of which died before reaching adulthood (Figure 1D, middle).

To investigate the cause of death, we collected blood and hearts at PD14. We did not observe cytopenia or leukocytosis (Figure S2), which would be associated with a hematological or infectious cause of death, respectively. However, the relative distribution of leukocytes skewed toward the myeloid lineage in *Mapk1* mutants (Figure S2), consistent with the known role of ERK in promoting myeloid cell differentiation.[41] Postnatal hearts in *Mapk1*$^{A172V/+}$ and *Mapk1*$^{A172V/A172V}$ mice displayed no lethal abnormalities (e.g., valvular, septal, large vessel, or myocardial defects; Figure S3), which is consistent with individuals with MRR that experienced mild or no congenital heart defects.[23] Because MEK (the MAPK immediately upstream of ERK) inhibition reduced growth-factor stimulated ERK phosphorylation in MRR patient cells,[23] we hypothesized that the US Food and Drug Administration (FDA)-approved MEK inhibitor mirdametinib (PD0325901) would improve the survival of *Mapk1*$^{A172V/A172V}$ mutants. Using the "MEK-in-milk" protocol,[30,42,43] we administered mirdametinib to pups via the mother's milk beginning 12 h after birth (PD0.5) until PD21. In vehicle-treated cages, a high rate of mortality was observed in *Mapk1*$^{A172V/A172V}$ pups, and the genotype frequency deviated from Mendelian ratios at PD21 (Figure 1E, left). However, no deaths occurred in mirdametinib-treated cages, restoring Mendelian ratios (Figure 1E, right) and confirming that downstream MAPK activation is pathological in mice modeling MRR.

Given that individuals with MRR carry a single pathogenic allele, we focused phenotyping efforts on *Mapk1*$^{A172V/+}$ mice. *Mapk1*$^{A172V/+}$ mice that survived past PD21 developed normally but had reduced body mass across their lifespan (Figure 1F), mirroring postnatal growth reduction in individuals with MRR.[23] In young mice, this was attributed to a reduction in body size (Figure 1B). For example, juvenile mutants had lower heart weight than controls, which significantly correlated with body size (Figure S3E). *Mapk1*$^{A172V/+}$ mice also had smaller skulls (Figure 1G) without a change in aspect ratio (Figure S4). Subsequent analysis of facial bone dimensions revealed a reduction in the nasal bone length in *Mapk1*$^{A172V/+}$ mice that persisted after normalization to account for differences in skull size (Figure 1G, right; Figure S4), recapitulating facial dysmorphism in NS mouse models.[19,44–47] In adult *Mapk1*$^{A172V/+}$ mice, lower body mass was due to leaner body type rather than decreased body length (Figure 1H). This phenotype was most obvious in older male *Mapk1*$^{A172V/+}$ mice (Figure S5) and was not associated with differences in home-cage activity level (Figure 1I) or steady-state metabolism (Table 1), which has been observed in other Rasopathy models.[47–49] Thus, ERK2 activation is sufficient to recapitulate growth and craniofacial abnormalities observed in individuals with MRR and Rasopathy mouse models.

Neurodevelopmental effects of enhanced ERK2 signaling in Mapk1$^{A172V/+}$ mice

Individuals with Rasopathies may experience neurocognitive dysfunction as well as abnormalities in brain morphology, white matter, and functional connectivity.[50–54] Therefore, we used a multidisciplinary approach to examine brain structure and function in *Mapk1*$^{A172V/+}$ mice. First, we evaluated the time course of ERK phosphorylation changes in the forebrain samples from mutant and control mice (Figure 2A). On embryonic day 16.5 (ED16.5), ERK2 activation was higher in *Mapk1*$^{A172V/+}$ forebrains relative to controls (Figure 2A, left) without a change in phosphorylated ERK1 or total ERK levels (Figure S6). Likewise, at ED18.5, cortical and hippocampal expression of the transcription factor *Etv5* (Figure 2B), a downstream marker for MAPK pathway activation in the developing brain,[38] was expanded in *Mapk1*$^{A172V/+}$ mice relative to *Mapk1*$^{+/+}$ littermates. Increased forebrain ERK2 phosphorylation in *Mapk1*$^{A172V/+}$ mice persisted postnatally and was largest at PD15 (Figure 2A, middle) but was least robust in adulthood (Figure 2A, right). Phosphoproteomic analysis of PD15 forebrains identified 185 phosphopeptides with an abundance that was more than 1.5-fold different in *Mapk1*$^{A172V/+}$ samples (71 lower, 114 higher) and had a *p* value of less than 0.05 (Figure 2C). These included peptides that mapped to proteins critical for brain development and function, including doublecortin (at sites S332 and S306), Map1b/Map2 (at multiple phosphorylation sites), histone deacetylase 6 (at S21), presenilin-1 (at S367), Syngap1 (at S780), and GABA-A receptor subunit A3 (at S433) (a complete list is provided in the source data and statistical analysis file). When genes associated with significant phosphopeptides were used to identify differentially regulated biological pathways,[55] the top Reactome terms related to Ras/MAPK signaling or known functions of this pathway (e.g., cellular responses to stress, cellular senescence, synaptic transmission) (Figure 2D). To confirm that these changes related to ERK activation, we performed PTM-SEA[56] and observed that the ERK1/2 PTM-SEA score was significantly increased in *Mapk1*$^{A172V/+}$ forebrains relative to wild-type controls (Figure 2E). Therefore, ERK activity is enhanced in the brains of mice modeling MRR.

MAPK signaling is required for the generation of glial cells,[57,58] and dysregulated MAPK signaling alters their number and function in the brain.[59–61] For example, upstream MAPK activation by *Nf1* loss or *Ptpn11* activation causes oligodendrocyte precursor cell (OPC) expansion, myelin decompaction, and reactive astrogliosis.[62–66]

Table 1. Metabolomic data from the liver and plasma of *Mapk1^{A172V}* mice

Liver				Plasma			
Metabolite (μmol/g)	*Mapk1*$^{+/+}$	*Mapk1*$^{A172V/+}$	*p* value	metabolite (mM)	*Mapk1*$^{+/+}$	*Mapk1*$^{A172V/+}$	*p* value
3-Hydroxybutyrate	0.15 ± 0.03	0.16 ± 0.03	0.73	3-Hydroxybutyrate	4.92 ± 0.93	5.37 ± 1.31	0.78
ADP	0.64 ± 0.06	0.73 ± 0.09	0.43	3-Hydroxyisobutyrate	1.32 ± 0.12	1.41 ± 0.08	0.53
AMP	0.47 ± 0.07	0.59 ± 0.11	0.37	Acetate	8.23 ± 0.63	8.21 ± 0.49	0.98
ATP	1.30 ± 0.08	1.09 ± 0.06	0.07	Acetoacetate	0.97 ± 0.13	1.05 ± 0.21	0.74
Acetate	0.22 ± 0.03	0.19 ± 0.04	0.61	Acetone	0.15 ± 0.02	0.15 ± 0.01	0.82
Alanine	2.49 ± 0.33	2.35 ± 0.23	0.75	Alanine	26.66 ± 2.86	27.22 ± 2.53	0.89
Ascorbate	0.89 ± 0.12	0.70 ± 0.10	0.25	Arabinose	1.94 ± 0.56	1.15 ± 0.08	0.22
Asparagine	0.058 ± 0.01	0.063 ± 0.01	0.62	Ascorbate	2.48 ± 0.30	1.65 ± 0.18	**0.048**
Aspartate	0.40 ± 0.05	0.48 ± 0.08	0.46	Asparagine	1.56 ± 0.28	1.75 ± 0.20	0.61
Betaine	1.47 ± 0.26	1.72 ± 0.41	0.60	Betaine	2.73 ± 0.30	2.43 ± 0.33	0.51
Cholate	0.23 ± 0.08	0.14 ± 0.03	0.32	Choline	0.40 ± 0.06	0.32 ± 0.02	0.23
Choline	0.058 ± 0.01	0.073 ± 0.01	0.39	Citrate	8.88 ± 0.52	10.20 ± 0.53	0.10
Creatine	0.13 ± 0.02	0.12 ± 0.02	0.79	Creatine	7.28 ± 1.39	5.28 ± 0.67	0.25
Creatinine	0.033 ± 0.003	0.038 ± 0.004	0.36	Formate	0.84 ± 0.15	0.77 ± 0.09	0.72
Dimethylamine	0.041 ± 0.004	0.043 ± 0.004	0.71	Fumarate	0.33 ± 0.07	0.35 ± 0.10	0.86
Fumarate	0.043 ± 0.01	0.043 ± 0.01	0.96	Glucose	221.01 ± 13.31	226.66 ± 7.96	0.73
GTP	0.16 ± 0.02	0.15 ± 0.02	0.60	Glutamine	12.45 ± 0.75	15.51 ± 0.67	0.96
Glucose	6.73 ± 0.48	8.31 ± 0.94	0.15	Glutathione	0.67 ± 0.25	0.35 ± 0.03	0.27
Glutamate	0.47 ± 0.08	0.61 ± 0.10	0.29	Glycine	8.57 ± 0.51	6.96 ± 1.45	0.29
Glutamine	1.80 ± 0.18	2.07 ± 0.22	0.35	Histidine	2.93 ± 0.20	2.72 ± 0.18	0.46
Glutathione	1.83 ± 0.19	1.56 ± 0.05	0.22	Inosine	0.24 ± 0.05	0.13 ± 0.05	0.15
Glycine	1.19 ± 0.14	1.22 ± 0.10	0.83	Isoleucine	3.06 ± 0.27	3.68 ± 0.25	0.12
Glycocholate	0.30 ± 0.10	0.16 ± 0.03	0.23	Lactate	171.22 ± 21.77	164.61 ± 13.85	0.81
Histidine	0.44 ± 0.04	0.46 ± 0.04	0.71	Leucine	4.59 ± 0.34	5.12 ± 0.28	0.27
IMP	0.029 ± 0.01	0.035 ± 0.01	0.59	Lysine	7.67 ± 0.66	7.82 ± 0.66	0.88
Inosine	0.023 ± 0.003	0.027 ± 0.01	0.51	Malate	4.02 ± 0.36	3.47 ± 1.04	0.60
Isoleucine	0.10 ± 0.02	0.12 ± 0.02	0.49	Methionine	2.24 ± 0.05	2.24 ± 0.17	0.99
Lactate	5.12 ± 0.58	4.83 ± 0.36	0.69	O-Acetylcarnitine	0.57 ± 0.06	0.52 ± 0.05	0.48
Leucine	0.22 ± 0.04	0.20 ± 0.03	0.66	O-Phosphocholine	0.14 ± 0.02	0.094 ± 0.01	0.10
Lysine	0.23 ± 0.04	0.26 ± 0.04	0.63	Phenylalanine	1.53 ± 0.10	1.68 ± 0.07	0.23
Malate	0.72 ± 0.11	0.80 ± 0.14	0.67	Proline	5.16 ± 0.26	4.81 ± 0.38	0.45
NAD+	0.46 ± 0.04	0.48 ± 0.05	0.79	Pyruvate	4.59 ± 0.71	4.52 ± 0.61	0.94
NADP+	0.10 ± 0.01	0.12 ± 0.01	0.25	Serine	4.88 ± 0.40	5.74 ± 0.56	0.23
Niacinamide	0.069 ± 0.01	0.081 ± 0.02	0.63	Succinate	0.79 ± 0.15	0.56 ± 0.18	0.35
O-Acetylcarnitine	0.073 ± 0.01	0.08 ± 0.01	0.61	Taurine	14.63 ± 2.30	11.80 ± 1.25	0.32
O-Phosphocholine	0.74 ± 0.08	1.08 ± 0.23	0.17	Threonine	6.49 ± 0.24	7.01 ± 0.38	0.26
Phenylalanine	0.053 ± 0.01	0.058 ± 0.01	0.58	Tyrosine	2.19 ± 0.15	2.39 ± 0.26	0.52
Succinate	0.49 ± 0.04	0.55 ± 0.06	0.43	Valine	6.30 ± 0.48	8.34 ± 0.27	**0.005**
Taurine	7.85 ± 1.08	9.65 ± 1.34	0.31	Myo-inositol	2.51 ± 0.18	2.16 ± 0.16	0.18
Threonine	0.40 ± 0.08	0.41 ± 0.07	0.86	sn-glycero-3-Phosphocholine	0.88 ± 0.05	0.78 ± 0.04	0.11

(*Continued on next page*)

Table 1. *Continued*

Liver				Plasma			
Metabolite (μmol/g)	*Mapk1*$^{+/+}$	*Mapk1*$^{A172V/+}$	*p* value	metabolite (mM)	*Mapk1*$^{+/+}$	*Mapk1*$^{A172V/+}$	*p* value
Tyrosine	0.061 ± 0.01	0.07 ± 0.01	0.56	–	–	–	–
UDP-N-Acetylglucosamine	0.16 ± 0.02	0.16 ± 0.02	0.88	–	–	–	–
UDP-galactose	0.11 ± 0.01	0.12 ± 0.01	0.50	–	–	–	–
UDP-glucose	0.32 ± 0.02	0.32 ± 0.03	0.87	–	–	–	–
UDP-glucuronate	0.089 ± 0.01	0.11 ± 0.02	0.26	–	–	–	–
Uridine	0.023 ± 0.003	0.032 ± 0.01	0.20	–	–	–	–
Valine	0.17 ± 0.03	0.21 ± 0.03	0.43	–	–	–	–
myo-Inositol	0.27 ± 0.03	0.29 ± 0.04	0.68	–	–	–	–
sn-Glycero-3-phosphocholine	0.38 ± 0.10	0.33 ± 0.07	0.75	–	–	–	–

Liver and plasma metabolites in *Mapk1* mutant and control mice. Metabolomic analysis of adult *Mapk1*$^{A172V/+}$ and *Mapk1*$^{+/+}$ mouse liver and plasma samples ($n_{+/+} = 7$, $n_{A172V/+} = 6$) showing steady-state metabolite levels. Genotypic differences were examined using unpaired *t* tests ($p < 0.05$ shown in bold). Data are presented as mean ± SEM.

Therefore, we examined the effects of ERK2 gain of function on glial populations by staining PD14 brains for markers Pdgfrα, Nkx2.2, Myrf, and GFAP, which label OPCs, OPCs/early differentiating oligodendrocytes, mature oligodendrocytes, and reactive astrocytes, respectively. Like mouse models of NF1 and NS,[63,65–67] we observed an increase in the number of OPCs (Figures 3A–3F) in the brains of *Mapk1*$^{A172V/+}$ mice. While no difference in the number of Pdgfrα-positive cells (Figure 3A) was observed in the cerebral cortex (Figure 3B), OPC abundance was increased in the corpus callosum (Figure 3C) in mutant mice relative to controls. We also observed an increased number of Nkx2.2-positive OPCs/differentiating oligodendrocytes (Figure 3D) in both the cortex (Figure 3E) and corpus callosum (Figure 3F) in *Mapk1*$^{A172V/+}$ mice without a change in the number of mature oligodendrocytes (Figures 3G–3I). At 10 weeks of age, we examined mature white-matter structure using transmission electron microscopy (Figure 3J). Like mice with oligodendrocyte-specific deletion of *Nf1*,[64,68] a gain-of-function *HRas* variant,[69] or mutant *Ptpn11*,[65] colossal white matter in *Mapk1*$^{A172V/+}$ mice displayed evidence of myelin decompaction (Figure 3K). An expansion of GFAP expression, indicating reactive gliosis, was observed in subcortical structures, most prominently the diencephalon and hypothalamus, at this time point (Figure S7). Thus, downstream MAPK activation via ERK2 gain-of-function causes glial abnormalities that genocopy those observed in mice with activating mutations upstream or at the level of Ras, such as *Nf1* loss or *Ptpn11*^{Q79R} expression.

Mapk1$^{A172V/+}$ mice recapitulate spatial memory deficits and sensory hypersensitivity observed in other Rasopathy mouse models

Individuals with MRR may exhibit intellectual disability, ADHD, reduced stress tolerance, and aggressive behavior.[23] To determine the neurocognitive effects of ERK2 gain of function *in vivo*, we performed a battery of behavioral tests that assessed different aspects of neurological function in *Mapk1*$^{A172V/+}$ and *Mapk1*$^{+/+}$ littermates. While *Mapk1* mutant mice performed similarly to wild types in tests of motor function (e.g., open-field locomotor activity, home-cage activity monitoring, sucrose splash test; Figures S8A and S8B; Figure 1I), anxiety-like behavior (marble-burying assay, dark/light box; Figures S8C and S8D), short-term memory (novel-object recognition; Figure S8E), and associative learning (contextual fear conditioning; Figure S8F), unique phenotypes were identified. First, we observed that *Mapk1*$^{A172V/+}$ mice—like other Rasopathy models[70–75]—were slower to learn the location of a submerged platform using environmental cues in the Morris water maze, which assays spatial memory.[76] Specifically, they had longer latencies to the platform during the first 2 days of testing (the training day when the platform was visible and acquisition day 1 when the platform was submerged and distal cues were present) (Figures 4A and 4B). With additional training, *Mapk1* mutants were able to achieve proficiency in the task, exhibiting no differences in platform latency during days 2–5 of the acquisition phase (Figure 4B) or during the probe trial (Figure S8G), in which the platform is removed and the ability to remember its previous location is assessed. No differences in reversal learning were observed (Figure S8H). Thus, enhanced ERK2 activity in the brain alters some but not all aspects of memory function and is sufficient to recapitulate Morris water maze deficits observed in mouse models of NF1 and NS.

Hypersensitivity to sensory stimuli is a common feature of autism and is prevalent in individuals with Rasopathies and co-morbid ASD.[10,77] Previously, we showed that mice modeling NF1 are more sensitive than wild-type littermates to arousing visual stimuli,[78] such as threatening looming disks that simulate an aerial predator

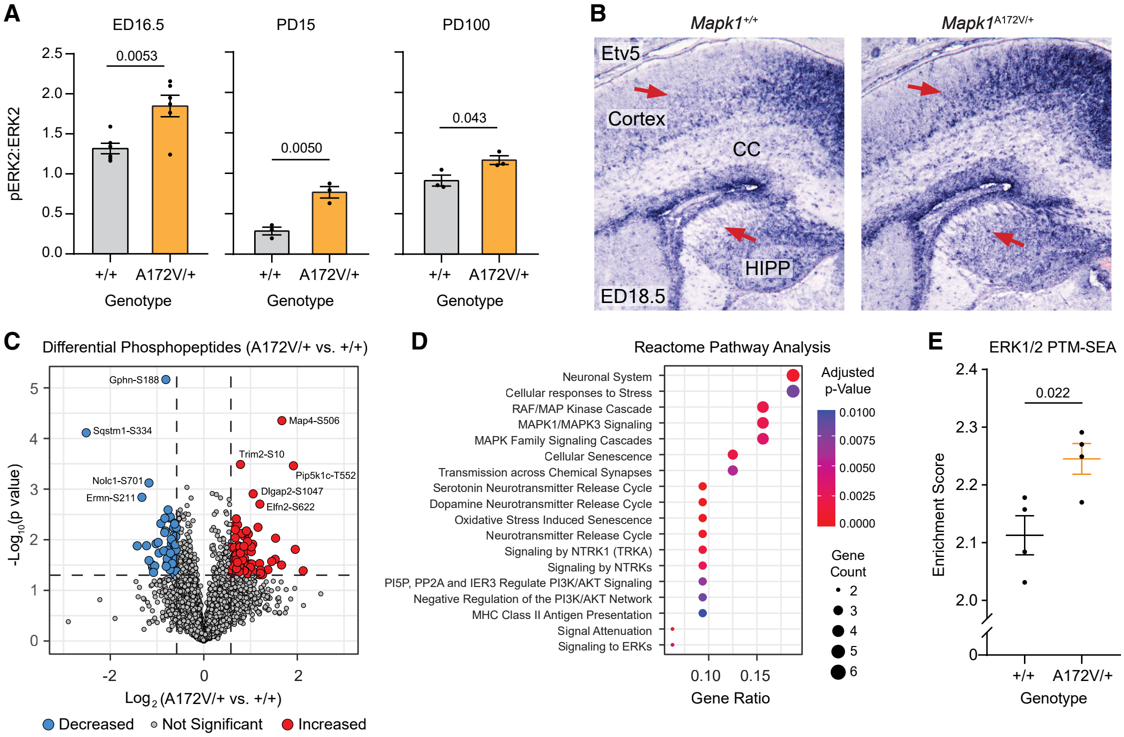

Figure 2. Proteomic characterization of ERK2 activity in the brains of *Mapk1*^{A172V} mice
(A) The pERK2:ERK2 ratio was increased in *Mapk1*$^{A172V/+}$ mouse forebrain samples relative to controls at embryonic day 16.5 (ED16.5) (left; $n_{+/+} = 6$; $n_{A172V/+} = 6$; unpaired t test; $t_{10} = 3.55$, $p = 0.0053$), PD15 (middle; $n_{+/+} = 3$; $n_{A172V/+} = 3$; unpaired t test; $t_4 = 5.58$, $p = 0.0050$), and PD100 (right; $n_{+/+} = 3$; $n_{A172V/+} = 3$; unpaired t test; $t_4 = 2.93$, $p = 0.043$).
(B) Representative images showing *Etv5* expression in *Mapk1*$^{+/+}$ and *Mapk1*$^{A172V/+}$ mouse brains at ED18.5. Red arrows demonstrate expansion of *Etv5* expression in the cortex and hippocampus (HIPP) of *Mapk1*$^{A172V/+}$ mice. CC, corpus callosum.
(C) Volcano plot showing 5025 unique phosphopeptides that were detected in *Mapk1*$^{A172V/+}$ and control forebrain samples ($n = 4$ per genotype) at PD15. Colored circles denote phosphopeptides > 1.5-fold increased (red) or decreased (blue) versus control with $p \leq 0.05$ (horizontal dashed line).
(D) Reactome dot plot showing biological pathways significantly altered in *Mapk1*$^{A172V/+}$ forebrain samples.
(E) PTM-SEA analysis showed greater enrichment of ERK1/2 phosphorylation targets in *Mapk1*$^{A172V/+}$ mice relative to *Mapk1*$^{+/+}$ controls ($n_{+/+} = 4$; $n_{A172V/+} = 4$; unpaired t test; $t_6 = 3.08$, $p = 0.022$). Data are presented as mean ± SEM.

approaching from above. In a looming-stimulus assay, presentation of a train of expanding, dark, overhead disks on a light background promotes rapid flight to shelter followed by periods of freezing in rodents.[79] Like *Nf1*$^{+/-}$ mice, *Mapk1*$^{A172V/+}$ mice exhibited more robust behavioral responses to looming visual threats than littermate controls, including higher rates of escape (Figure 4C), increased post-stimulus velocity (Figure 4E, left), a shorter latency to freeze (Figure 4E, middle), and longer freezing duration (Figure 4E, right) in response to the train of looming disks without a difference in the distance traveled during the pre-stimulus habituation period (Figure 4F). To assay other sensory systems, we measured pre-pulse inhibition of the acoustic startle reflex in *Mapk1*$^{A172V/+}$ and *Mapk1*$^{+/+}$ mice. While sensory gating (i.e., pre-pulse inhibition) was intact in *Mapk1*$^{A172V/+}$ mice, they exhibited more robust startle reflexes than littermate control mice when exposed to a loud (120 dB) acoustic stimulus (Figure 4G). This phenotype mirrors acoustic startle hypersensitivity previously observed in mice with biallelic *Nf1* loss in oligodendrocytes.[64] In addition to acoustic stimuli, *Mapk1* mutants had a shorter latency to groom in the sucrose splash test (Figure S8B) without a change in the total grooming time, suggesting that they may be more sensitive to tactile stimuli. Therefore, ERK2 gain of function in *Mapk1*$^{A172V/+}$ mice causes hypersensitivity to aversive sensory stimuli in mice, providing evidence that these phenotypes result from aberrant downstream MAPK signaling in mouse models of NF1 and other Rasopathy syndromes.

Discussion

In these studies, we generated a genetically engineered mouse model of *MAPK1*-related Rasopathy (*Mapk1*$^{A172V/+}$ mice), which recapitulates several clinical features associated with MRR: small stature, craniofacial dysmorphism, and cognitive impairment. Because the *Mapk1*^{A172V} pathogenic variant near the ERK2 activation domain increased kinase activity, we were able to address an important unanswered question in the Rasopathy field: to what extent do mice with gain-of-function mutations in downstream Ras effectors, such as the terminal MAPK ERK, genocopy

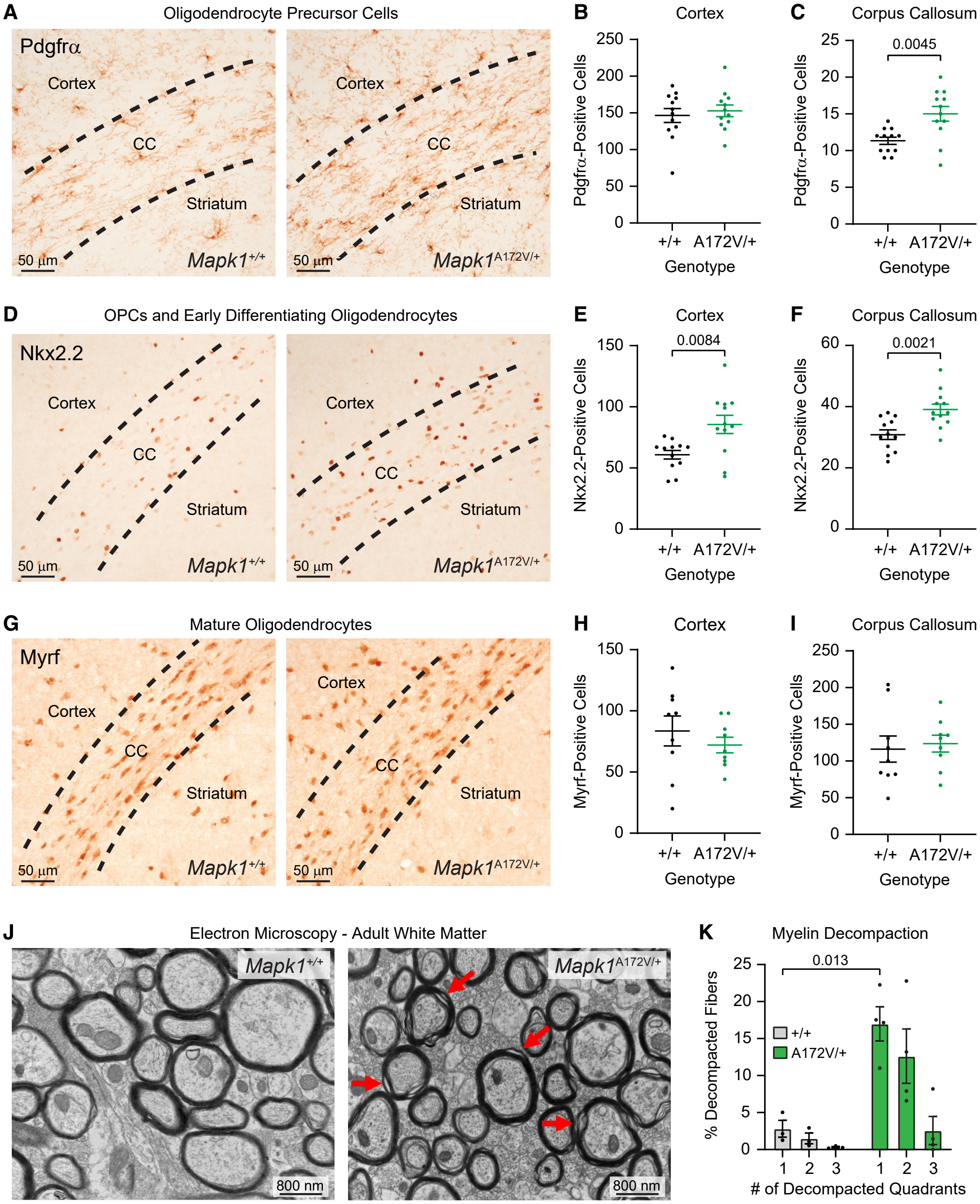

Figure 3. Examination of oligodendrocyte lineage defects in *Mapk1*$^{A172V/+}$ mice

(A) Representative images showing Pdgfrα-stained oligodendrocyte precursor cells (OPCs) in PD14 *Mapk1*$^{+/+}$ (left) and *Mapk1*$^{A172V/+}$ mice (right).

(B and C) There were no genotypic differences in the number of OPCs in the cerebral cortex (B; $n_{+/+} = 12$, $n_{A172V/+} = 12$; unpaired *t* test; $t_{22} = 0.51$, $p = 0.61$), but more OPCs were observed in the corpus callosum (CC) in *Mapk1*$^{A172V/+}$ brains relative to controls (C; $n_{+/+} = 12$, $n_{A172V/+} = 12$; unpaired *t* test with Welch's correction; $t_{15.85} = 3.30$, $p = 0.0045$).

(D) Representative images of Nkx2.2-stained OPCs and differentiating early oligodendrocytes in PD14 *Mapk1*$^{+/+}$ (left) and *Mapk1*$^{A172V/+}$ mice (right).

(E and F) Nkx2.2-positive cells were increased in the cortex (E; $n_{+/+} = 12$, $n_{A172V/+} = 12$; unpaired *t* test with Welch's correction; $t_{15.69} = 3.012$, $p = 0.0084$) and CC (F; $n_{+/+} = 12$, $n_{A172V/+} = 12$; unpaired *t* test; $t_{22} = 3.49$ $p = 0.0021$) in *Mapk1*$^{A172V/+}$ mice.

(G) Representative images of Myrf-stained mature oligodendrocytes in PD14 *Mapk1*$^{+/+}$ (left) and *Mapk1*$^{A172V/+}$ mice (right).

(H and I) There were no differences in Myrf-positive cells in the cortex (H; $n_{+/+} = 9$, $n_{A172V/+} = 9$; unpaired *t* test; $t_{16} = 0.83$, $p = 0.42$) or CC (I; $n_{+/+} = 9$, $n_{A172V/+} = 9$; unpaired *t* test; $t_{16} = 0.35$ $p = 0.73$).

(J) Representative electron micrographs of CC samples from *Mapk1*$^{+/+}$ (left) and *Mapk1*$^{A172V/+}$ (right) mice at 10 weeks of age. Red arrows demonstrate areas of myelin decompaction.

(legend continued on next page)

Rasopathy model mice with genetic perturbations acting upstream or at the level of Ras? We found that ERK2 gain of function is sufficient to produce a core set of central nervous system cellular, histological, and functional phenotypes observed in Rasopathy mouse models with upstream mutations: oligodendrocyte lineage defects, reactive astrocytosis, deficits in hippocampus-dependent spatial learning, and increased reactivity to arousing sensory stimuli, such as looming visual threats.

The Ras/MAPK signaling pathway plays a critical role in embryonic and postnatal brain development, and we believe that the *Mapk1*^{A172V} mouse model will provide the community with a useful tool for studying the role of downstream MAPKs in neurodevelopment and, subsequently, neurodevelopmental symptoms associated with Rasopathy syndromes. Previous studies have shown that MAPKs regulate neural progenitor cell (NPC) fate decisions via phosphorylation,[80] and ERK1/2 determines the balance between proliferative and neurogenic progenitor divisions via cyclin D1 and p27kip1.[81] Dysregulation of these effectors leads to cell-cycle elongation, which causes a shift toward neurogenic versus self-renewing cell division.[81] In radial glia, MEK1/2 deletion is associated with loss of glia-like biochemical properties by ED17.5 and reduced glial specification, while expression of a constitutively activated MEK1/2 variant in the same lineage increases the number of astrocyte precursors and mature astrocytes.[57] ERK2-knockout mice display enhanced proliferation of NPCs, reduced proliferation of glial progenitors, and disrupted cortical lamination that is measurable from embryonic day ED15.5 to PD10.[82–84] In these mice, ERK2 loss alters the excitability of cortical neurons and disrupts neural activity patterns, leading to deficits in working and hippocampus-dependent memory at 3 months of age.[81] Thus, there is evidence that early perturbations in ERK2 activity produces structural and functional changes in the brain that negatively affect cognitive function. Given these findings, future experiments will be needed to determine how aberrant neurodevelopment in *Mapk1*$^{A172V/+}$ mice mechanistically causes behavioral phenotypes, as well as to determine if there is a critical window for pharmacological intervention in this model.

Consistent findings in mice modeling Rasopathy syndromes have been abnormalities in the generation and function of glial cell populations in the brain. Like mice modeling NF1[63,64,66–68] and NS,[65] the brains of *Mapk1*$^{A172V/+}$ mice had distinct oligodendrocyte lineage phenotypes with an increased number of Nkx2.2- and Pdgfrα-positive OPCs and evidence of myelin decompaction in adulthood. We also found increased GFAP staining, an indicator for reactive gliosis that has also been observed in Rasopathy mouse models.[62,65,85] The Ras/MAPK pathway is required for gliogenesis, as deletion of MEK1/2 results in a cortex without astrocytes and oligodendrocytes, and hyperactivation of MEK1 increases astrocyte[57] and OPC abundance.[38] Although *Ptpn11* loss of function reduces OPC generation, NS-associated *Ptpn11* mutants increase the number of OPCs and cause abnormal myelination.[65] To date, the ERK transcriptional targets driving aberrant oligodendrogenesis have not yet been identified. In Ras/MAPK mutant cancers, a conserved gene signature that includes *ETV4/5*, *SPRY2/4*, *DUSP4/6*, and *EPHA2/4* is a reliable biomarker of pathway activation and is associated with good clinical response to MEK inhibitor therapy.[86] Of these, the ETS transcription factor Etv5 is a strong candidate to play a mechanistic role in MRR-related deficits given its upregulation in *Mapk1*$^{A172V/+}$ mice and involvement in the generation and maturation of neurons and glia in the hippocampus, cortex, and other brain regions.[87–91] Etv5 is also required for the maintenance of hippocampal-neuron dendritic morphology and plasticity, as well as hippocampus-dependent learning,[88] in mice. Thus, future efforts will be required to explore the role of Etv5 and other ERK targets in OPC and cognitive deficits in MRR.

In addition to changes in OPC number during early postnatal development, *Mapk1*$^{A172V/+}$ mice had evidence of myelin defects in mature white matter. Myelin decompaction has been observed in mice with conditional deletion of *Nf1*[64,68,92] or expression of activated HRas[68,69] or Shp2[65] in oligodendrocytes. *Hras*^{G12V} or *Nf1*$^{-/-}$ conditional mutants also have enhanced acoustic startle responses,[64,68,92] which raises the possibility that myelin decompaction in *Mapk1*$^{A172V/+}$ mice causes sensory hypersensitivity phenotypes. In oligodendrocyte-targeted Rasopathy mouse models, myelination defects are MAPK, nitric oxide, and Notch dependent.[64,68,69] Systemic treatment with the MEK inhibitor mirdametinib improved cell-autonomous white-matter phenotypes in *Nf1* conditional-knockout mice,[64,68] providing a roadmap for future mechanistic studies and/or preclinical therapeutic trials in MRR models. MEK inhibitors have an established safety profile, are FDA approved for use in the treatment of NF1-associated tumors,[93] and prevented postnatal mortality in *Mapk1*$^{A172V/A172V}$ mice. Dosing in *Mapk1* mutant mice will need to be carefully titrated, however, as mirdametinib treatment alone can perturb myelin structure in wild-type animals,[69] suggesting that the regulation of myelin integrity by MAPK signaling is tightly controlled.

Mapk1$^{A172V/+}$ mice also exhibited deficits in hippocampus-dependent spatial learning in the Morris water maze. *Mapk1* mutants had longer trial times during initial

(K) There was a significant interaction between the number of decompacted quadrants and mouse genotype ($n_{+/+} = 3$, $n_{A172V/+} = 4$; two-way repeated-measures ANOVA; $F_{2,10} = 6.92$, $p_{\text{Decompacted Quadrants} \times \text{Genotype}} = 0.013$; $F_{1.51,7.56} = 13.41$, $p_{\text{Decompacted Quadrants}} = 0.0045$; $F_{1,5} = 11.69$, $p_{\text{Genotype}} = 0.019$) specifically in myelinated axons with one decompacted quadrant (Bonferroni *post hoc* test; $p = 0.013$).
Scale bars in (A), (D), and (G): 50 μm. Scale bars in (J): 800 nm. Data are presented as mean ± SEM.

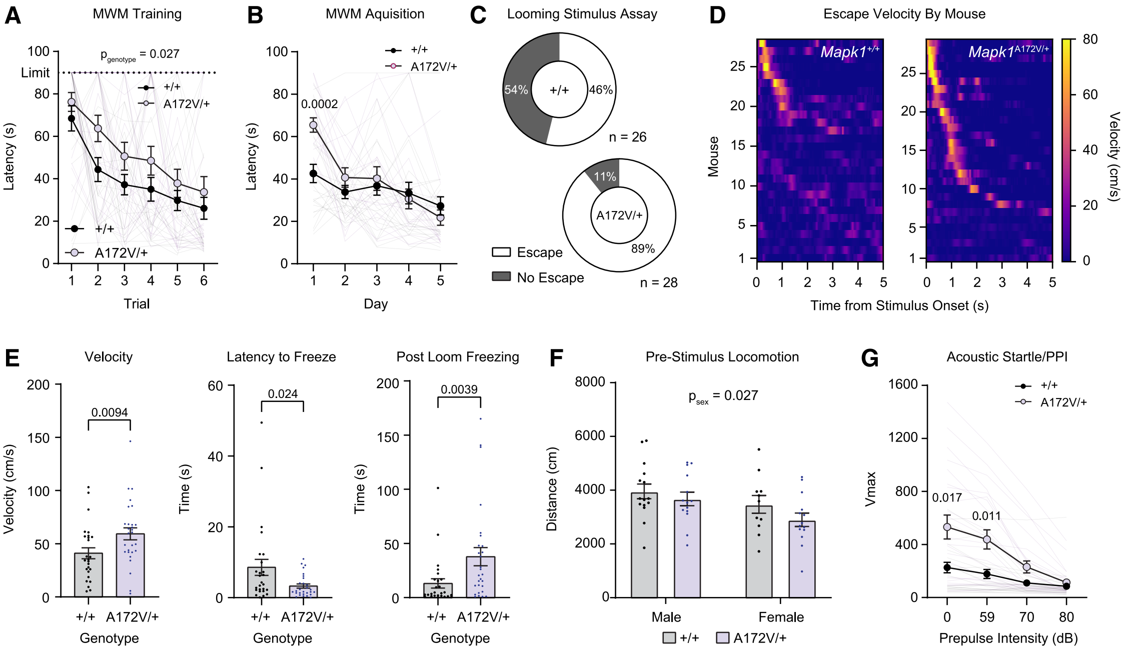

Figure 4. Behavioral characterization of *Mapk1*$^{A172V/+}$ mice

(A) *Mapk1*$^{A172V/+}$ mice had a longer latency to find the platform during Morris water-maze (MWM) training ($n_{+/+} = 25$, $n_{A172V/+} = 22$; two-way repeated-measures ANOVA; $F_{5,225} = 0.38$, $p_{trial \times genotype} = 0.86$; $F_{1,45} = 5.25$, $p_{genotype} = 0.027$; $F_{4.47,201.4} = 19.48$, $p_{trial} < 0.0001$). Four *Mapk1*$^{A172V/+}$ mice failed to locate the platform and were excluded from further testing.

(B) During the MWM acquisition phase, there was a significant day $\times$ genotype interaction ($n_{+/+} = 25$, $n_{A172V/+} = 18$; $F_{4,164} = 5.44$, $p = 0.0004$), and *Mapk1*$^{A172V/+}$ mice had a longer latency to find the platform on the first acquisition day (Bonferroni *post hoc* test; $p = 0.0002$) compared to controls.

(C) *Mapk1*$^{A172V/+}$ mice had a higher probability of escape than *Mapk1*$^{+/+}$ mice in the looming-stimulus assay ($n_{+/+} = 26$, $n_{A172V/+} = 28$; Chi-squared test; $X^2(2, N = 54) = 8.440$, $p = 0.0037$).

(D) Heatmaps showing looming disk-evoked changes in velocity in individual *Mapk1*$^{+/+}$ (left) and *Mapk1*$^{A172V/+}$ mice (right).

(E) *Mapk1*$^{A172V/+}$ mice had a higher peak velocity (left; Mann-Whitney U test, $U(n_{+/+} = 26, n_{A172V/+} = 28) = 215$, $p = 0.0094$), a shorter latency to freeze (middle; Mann-Whitney U test, $U(n_{+/+} = 26, n_{A172V/+} = 28) = 234.5$, $p = 0.024$), and increased post-loom freezing (right; Mann-Whitney U test, $U(n_{+/+} = 26, n_{A172V/+} = 28) = 199.5$, $p = 0.0039$).

(F) There were no differences in pre-stimulus locomotor activity between *Mapk1*$^{+/+}$ mice and *Mapk1*$^{A172V/+}$ mice during the looming-stimulus assay; however, a significant decrease in distance traveled was observed in females independently of genotype ($n_{+/+} = 26$, $n_{A172V/+} = 28$; two-way ANOVA; $F_{1,50} = 0.27$, $p_{sex \times genotype} = 0.60$; $F_{1,50} = 5.18$, $p_{sex} = 0.027$; $F_{1,50} = 2.38$, $p_{genotype} = 0.13$).

(G) There was a significant increase in the startle response of *Mapk1*$^{A172V/+}$ mice relative to controls ($n_{+/+} = 23$, $n_{A172V/+} = 23$; two-way repeated-measures ANOVA; $F_{3,132} = 10.17$, $p_{stimulus \times genotype} = < 0.0001$; $F_{1.67,51.29} = 40.61$, $p_{stimulus} < 0.0001$; $F_{1,44} = 8.45$, $p_{genotype} = 0.0057$). The startle response was significantly larger in response to the stimulus alone (Bonferroni *post hoc* test; $p = 0.017$) and in trials with the lowest intensity (59 dB) pre-pulse (Bonferroni *post hoc* test; $p = 0.011$).

Data are presented as mean $\pm$ SEM.

training and increased latency to locate the platform on early acquisition trials. This result is consistent with phenotypes reported in other Rasopathy mouse models, including NS, CS, and NF1,[70–75,94,95] as well as children with NF1 who participated in a virtual-reality Morris water-maze task.[96] Previous studies in *Nf1*$^{+/-}$ mice attributed learning deficits to impaired hippocampal long-term potentiation (LTP) caused by increased inhibitory GABA tone[70] that occurs via ERK-mediated phosphorylation of synapsin I.[71] This presynaptic terminal protein controls the ready releasable pool of synaptic vesicles by regulating interactions with actin.[97,98] Additionally, Ras/MAPK signaling in the postsynaptic compartment plays an established role in hippocampal LTP[99–102] by regulating AMPA receptor insertion[103,104] and dendritic spine structural plasticity.[105] Thus, spatial-learning deficits occurring in the context of *MAPK1* gain-of-function mutations are likely to have a complex neurophysiological etiology involving presynaptic regulation of GABAergic neurotransmission and/or changes in the postsynaptic compartment in glutamatergic pyramidal neurons. Future electrophysiological, molecular, and functional studies will be needed to test this hypothesis and clarify the role of different hippocampal cell types in spatial memory deficits in *Mapk1*$^{A172V/+}$ mice.

Finally, we observed that *Mapk1*$^{A172V/+}$ mice are hypersensitive to looming visual threats, a phenotype that we previously observed in *Nf1*$^{+/-}$ mice.[78] In rodents, the ability of looming visual threats to rapidly evoke an innate defensive behavior is dependent on an evolutionarily conserved circuit involving the midbrain superior colliculus,[106] which is directly innervated by retinal ganglion cells in its superficial layers.[107] The saliency, or perceived importance, of a looming disk is encoded by the activity

of glutamatergic neurons in the superior colliculus that, in turn, provide excitatory input to glutamatergic neurons in the dorsal periaqueductal gray (PAG) that compute escape decisions via a synaptic threshold mechanism.[108] The superior colliculus also innervates the midbrain ventral tegmental area, which can modulate looming-stimulus-evoked escape via GABAergic projections to the central nucleus of the amygdala[109] or dopaminergic projections to the nucleus accumbens medial shell.[28] Higher-order brain regions such as the retrosplenial,[110] visual,[111] and prefrontal[112] cortices provide descending input to the superior colliculus, which regulates different aspects of the escape behavior. At this time, it is unknown how supraphysiological MAPK signaling in subcortical sensory processing centers or the cortical sites that regulate them enhances threat reactivity in Rasopathy mice, which will need to be dissected in future studies.

Data and code availability

All source data used to produce the figures and tables, as well as the results of statistical testing procedures, are provided in the source data and statistical analysis file. The accession number for the raw NMR and mass spectrometry datasets is Synapse: https://www.synapse.org/Synapse:syn71742374/. The accession number for the code used for the looming-stimulus assay is GitHub: https://github.com/jelliottrobinson/BonsaiLoomStim.

Acknowledgments

This work was supported by NINDS project grant R01NS126108, SFARI Bridge to Independence Award 663007, CCHMC startup funds to J.E.R.; a CCHMC Center for Pediatric Genomics Pilot Award to R.R.W. and C.E.P.; NINDS training grant T32NS007453 to K.E.G.; and shared instrumentation grant S10OD026717 to K.D.G.

Declaration of interests

The authors declare no competing interests.

Supplemental information

Supplemental information can be found online at https://doi.org/10.1016/j.xhgg.2026.100621.

Web resources

Online Mendelian Inheritance in Man (OMIM), https://www.omim.org/

Received: January 6, 2026
Accepted: April 23, 2026

References

1. Boguski, M.S., and McCormick, F. (1993). Proteins regulating Ras and its relatives. Nature *366*, 643–654. https://doi.org/10.1038/366643a0.
2. Barbacid, M. (1987). Ras genes. Annu. Rev. Biochem. *56*, 779–827.
3. Lavoie, H., Gagnon, J., and Therrien, M. (2020). ERK signalling: a master regulator of cell behaviour, life and fate. Nat. Rev. Mol. Cell Biol. *21*, 607–632. https://doi.org/10.1038/s41580-020-0255-7.
4. Wortzel, I., and Seger, R. (2011). The ERK Cascade. Genes Cancer *2*, 195–209. https://doi.org/10.1177/1947601911407328.
5. Yoon, S., and Seger, R. (2006). The extracellular signal-regulated kinase: Multiple substrates regulate diverse cellular functions. Growth Factors *24*, 21–44. https://doi.org/10.1080/02699050500284218.
6. Rauen, K.A. (2013). The RASopathies. Annu. Rev. Genomics Hum. Genet. *14*, 355–369. https://doi.org/10.1146/annurev-genom-091212-153523.
7. Rauen, K.A. (2022). Defining RASopathy. Dis. Model. Mech. *15*, dmm049344. https://doi.org/10.1242/dmm.049344.
8. Tidyman, W.E., and Rauen, K.A. (2009). The RASopathies: developmental syndromes of Ras/MAPK pathway dysregulation. Curr. Opin. Genet. Dev. *19*, 230–236. https://doi.org/10.1016/j.gde.2009.04.001.
9. Roberts, A.E. (1993). Noonan Syndrome. In GeneReviews®, M.P. Adam, D.B. Everman, G.M. Mirzaa, R.A. Pagon, S.E. Wallace, L.J. Bean, K.W. Gripp, and A. Amemiya, eds. (University of Washington).
10. Adviento, B., Corbin, I.L., Widjaja, F., Desachy, G., Enrique, N., Rosser, T., Risi, S., Marco, E.J., Hendren, R.L., Bearden, C.E., et al. (2014). Autism traits in the RASopathies. J. Med. Genet. *51*, 10–20. https://doi.org/10.1136/jmedgenet-2013-101951.
11. Borrie, S.C., Brems, H., Legius, E., and Bagni, C. (2017). Cognitive Dysfunctions in Intellectual Disabilities: The Contributions of the Ras-MAPK and PI3K-AKT-mTOR Pathways. Annu. Rev. Genomics Hum. Genet. *18*, 115–142. https://doi.org/10.1146/annurev-genom-091416-035332.
12. Garg, S., Brooks, A., Burns, A., Burkitt-Wright, E., Kerr, B., Huson, S., Emsley, R., and Green, J. (2017). Autism spectrum disorder and other neurobehavioural comorbidities in rare disorders of the Ras/MAPK pathway. Dev. Med. Child Neurol. *59*, 544–549. https://doi.org/10.1111/dmcn.13394.
13. Geoffray, M.-M., Falissard, B., Green, J., Kerr, B., Evans, D.G., Huson, S., Burkitt-Wright, E., and Garg, S. (2021). Autism Spectrum Disorder Symptom Profile Across the RASopathies. Front. Psychiatry *11*, 585700. https://doi.org/10.3389/fpsyt.2020.585700.
14. Gupta, R., and Chogle, A. (2019). In Chapter 6 - Gastrointestinal Manifestations of Noonan Syndrome, N. Syndrome and A. Bhangoo, eds. (Academic Press), pp. 85–91. https://doi.org/10.1016/B978-0-12-815348-2.00005-0.
15. El Bouchikhi, I., Belhassan, K., Moufid, F.Z., Houssaini, M.I., Bouguenouch, L., Samri, I., Atmani, S., and Ouldim, K. (2016). Noonan syndrome-causing genes: Molecular update and an assessment of the mutation rate. Int. J. Pediatr. Adolesc. Med. *3*, 133–142. https://doi.org/10.1016/j.ijpam.2016.06.003.
16. Tartaglia, M., Mehler, E.L., Goldberg, R., Zampino, G., Brunner, H.G., Kremer, H., van der Burgt, I., Crosby, A.H., Ion, A., Jeffery, S., et al. (2001). Mutations in PTPN11, encoding the protein tyrosine phosphatase SHP-2, cause Noonan syndrome. Nat. Genet. *29*, 465–468. https://doi.org/10.1038/ng772.

17. Araki, T., Chan, G., Newbigging, S., Morikawa, L., Bronson, R.T., and Neel, B.G. (2009). Noonan syndrome cardiac defects are caused by PTPN11 acting in endocardium to enhance endocardial-mesenchymal transformation. Proc. Natl. Acad. Sci. USA *106*, 4736–4741. https://doi.org/10.1073/pnas.0810053106.
18. Hernández-Porras, I., Fabbiano, S., Schuhmacher, A.J., Aicher, A., Cañamero, M., Cámara, J.A., Cussó, L., Desco, M., Heeschen, C., Mulero, F., et al. (2014). K-RasV14I recapitulates Noonan syndrome in mice. Proc. Natl. Acad. Sci. USA *111*, 16395–16400. https://doi.org/10.1073/pnas.1418126111.
19. Takahara, S., Inoue, S.i., Miyagawa-Tomita, S., Matsuura, K., Nakashima, Y., Niihori, T., Matsubara, Y., Saiki, Y., and Aoki, Y. (2019). New Noonan syndrome model mice with RIT1 mutation exhibit cardiac hypertrophy and susceptibility to β-adrenergic stimulation-induced cardiac fibrosis. EBioMedicine *42*, 43–53. https://doi.org/10.1016/j.ebiom.2019.03.014.
20. Wong, J.C., Perez-Mancera, P.A., Huang, T.Q., Kim, J., Grego-Bessa, J., del pilar Alzamora, M., Kogan, S.C., Sharir, A., Keefe, S.H., Morales, C.E., et al. (2020). $Kras^{P34R}$ and $Kras^{T58I}$ mutations induce distinct RASopathy phenotypes in mice. JCI Insight *5*, e140495. https://doi.org/10.1172/jci.insight.140495.
21. Aksamitiene, E., Kiyatkin, A., and Kholodenko, B.N. (2012). Cross-talk between mitogenic Ras/MAPK and survival PI3K/Akt pathways: a fine balance. Biochem. Soc. Trans. *40*, 139–146. https://doi.org/10.1042/BST20110609.
22. Soriano, O., Alcón-Pérez, M., Vicente-Manzanares, M., and Castellano, E. (2021). The Crossroads between RAS and RHO Signaling Pathways in Cellular Transformation, Motility and Contraction. Genes *12*, 819. https://doi.org/10.3390/genes12060819.
23. Motta, M., Pannone, L., Pantaleoni, F., Bocchinfuso, G., Radio, F.C., Cecchetti, S., Ciolfi, A., Di Rocco, M., Elting, M.W., Brilstra, E.H., et al. (2020). Enhanced MAPK1 Function Causes a Neurodevelopmental Disorder within the RASopathy Clinical Spectrum. Am. J. Hum. Genet. *107*, 499–513. https://doi.org/10.1016/j.ajhg.2020.06.018.
24. Angoa-Pérez, M., Kane, M.J., Briggs, D.I., Francescutti, D.M., and Kuhn, D.M. (2013). Marble Burying and Nestlet Shredding as Tests of Repetitive, Compulsive-like Behaviors in Mice. JoVE J *82*, e50978. https://doi.org/10.3791/50978.
25. Frisbee, J.C., Brooks, S.D., Stanley, S.C., and d'Audiffret, A.C. (2015). An Unpredictable Chronic Mild Stress Protocol for Instigating Depressive Symptoms, Behavioral Changes and Negative Health Outcomes in Rodents. JoVE J *106*, e53109. https://doi.org/10.3791/53109.
26. Bevins, R.A., and Besheer, J. (2006). Object recognition in rats and mice: a one-trial non-matching-to-sample learning task to study "recognition memory.". Nat. Protoc. *1*, 1306–1311. https://doi.org/10.1038/nprot.2006.205.
27. Gonzalez, L.S., Fisher, A.A., D'Souza, S.P., Cotella, E.M., Lang, R.A., and Robinson, J.E. (2023). Ventral striatum dopamine release encodes unique properties of visual stimuli in mice. eLife *12*, e85064. https://doi.org/10.7554/eLife.85064.
28. Fisher, A.A., Gonzalez, L.S., Cappel, Z.R., Grover, K.E., Waclaw, R.R., and Robinson, J.E. (2025). Dopaminergic encoding of future defensive actions in the mouse nucleus accumbens. PNAS Nexus *4*, pgaf128. https://doi.org/10.1093/pnasnexus/pgaf128.
29. Lopes, G., Bonacchi, N., Frazão, J., Neto, J.P., Atallah, B.V., Soares, S., Moreira, L., Matias, S., Itskov, P.M., Correia, P.A., et al. (2015). Bonsai: an event-based framework for processing and controlling data streams. Front Neuroinform *9*, 7. https://doi.org/10.3389/fninf.2015.00007.
30. Jecrois, E.S., Zheng, W., Bornhorst, M., Li, Y., Treisman, D.M., Muguyo, D., Huynh, S., Andrew, S.F., Wang, Y., Jiang, J., et al. (2021). Treatment during a developmental window prevents NF1-associated optic pathway gliomas by targeting Erk-dependent migrating glial progenitors. Dev. Cell *56*, 2871–2885.e6. https://doi.org/10.1016/j.devcel.2021.08.004.
31. Wu, H., Southam, A.D., Hines, A., and Viant, M.R. (2008). High-throughput tissue extraction protocol for NMR- and MS-based metabolomics. Anal. Biochem. *372*, 204–212. https://doi.org/10.1016/j.ab.2007.10.002.
32. Schock, T.B., Duke, J., Goodson, A., Weldon, D., Brunson, J., Leffler, J.W., and Bearden, D.W. (2013). Evaluation of Pacific White Shrimp (Litopenaeus vannamei) Health during a Superintensive Aquaculture Growout Using NMR-Based Metabolomics. PLoS One *8*, e59521. https://doi.org/10.1371/journal.pone.0059521.
33. Watanabe, M., Meyer, K.A., Jackson, T.M., Schock, T.B., Johnson, W.E., and Bearden, D.W. (2015). Application of NMR-based metabolomics for environmental assessment in the Great Lakes using zebra mussel (Dreissena polymorpha). Metabolomics *11*, 1302–1315. https://doi.org/10.1007/s11306-015-0789-4.
34. Hasson, D.C., Watanabe-Chailland, M., Romick-Rosendale, L., Koterba, A., Miner, D.S., Lahni, P., Ma, Q., Goldstein, S.L., Devarajan, P., and Standage, S.W. (2022). Choline supplementation attenuates experimental sepsis-associated acute kidney injury. Am J Physiol Renal Physiol *323*, F255–F271. https://doi.org/10.1152/ajprenal.00033.2022.
35. McCauley, H.A., Riedman, A.M., Enriquez, J.R., Zhang, X., Watanabe-Chailland, M., Sanchez, J.G., Kechele, D.O., Paul, E.F., Riley, K., Burger, C., et al. (2023). Enteroendocrine Cells Protect the Stem Cell Niche by Regulating Crypt Metabolism in Response to Nutrients. Cell. Mol. Gastroenterol. Hepatol. *15*, 1293–1310. https://doi.org/10.1016/j.jcmgh.2022.12.016.
36. Wishart, D.S., Tzur, D., Knox, C., Eisner, R., Guo, A.C., Young, N., Cheng, D., Jewell, K., Arndt, D., Sawhney, S., et al. (2007). HMDB: the Human Metabolome Database. Nucleic Acids Res. *35*, D521–D526. https://doi.org/10.1093/nar/gkl923.
37. Chong, J., Wishart, D.S., and Xia, J. (2019). Using MetaboAnalyst 4.0 for Comprehensive and Integrative Metabolomics Data Analysis. Curr. Protoc. Bioinformatics *68*, e86. https://doi.org/10.1002/cpbi.86.
38. Talley, M.J., Nardini, D., Qin, S., Prada, C.E., Ehrman, L.A., and Waclaw, R.R. (2021). A role for sustained MAPK activity in the mouse ventral telencephalon. Dev. Biol. *476*, 137–147. https://doi.org/10.1016/j.ydbio.2021.03.019.
39. Russell, H.K. (1972). A modification of Movat's pentachrome stain. Arch. Pathol. *94*, 187–191.
40. Cargnello, M., and Roux, P.P. (2011). Activation and Function of the MAPKs and Their Substrates, the MAPK-Activated Protein Kinases. Microbiol. Mol. Biol. Rev. *75*, 50–83. https://doi.org/10.1128/mmbr.00031-10.

41. Hsu, C.-L., Kikuchi, K., and Kondo, M. (2007). Activation of mitogen-activated protein kinase kinase (MEK)/extracellular signal regulated kinase (ERK) signaling pathway is involved in myeloid lineage commitment. Blood *110*, 1420–1428. https://doi.org/10.1182/blood-2007-02-071761.
42. Wang, Y., Kim, E., Wang, X., Novitch, B.G., Yoshikawa, K., Chang, L.-S., and Zhu, Y. (2012). ERK inhibition rescues defects in fate specification of Nf1-deficient neural progenitors and brain abnormalities. Cell *150*, 816–830. https://doi.org/10.1016/j.cell.2012.06.034.
43. Kim, E., Wang, Y., Kim, S.-J., Bornhorst, M., Jecrois, E.S., Anthony, T.E., Wang, C., Li, Y.E., Guan, J.-L., Murphy, G.G., and Zhu, Y. (2014). Transient inhibition of the ERK pathway prevents cerebellar developmental defects and improves long-term motor functions in murine models of neurofibromatosis type 1. eLife *3*, e05151. https://doi.org/10.7554/eLife.05151.
44. Araki, T., Mohi, M.G., Ismat, F.A., Bronson, R.T., Williams, I.R., Kutok, J.L., Yang, W., Pao, L.I., Gilliland, D.G., Epstein, J.A., and Neel, B.G. (2004). Mouse model of Noonan syndrome reveals cell type- and gene dosage-dependent effects of Ptpn11 mutation. Nat. Med. *10*, 849–857. https://doi.org/10.1038/nm1084.
45. Nakamura, T., Gulick, J., Pratt, R., and Robbins, J. (2009). Noonan syndrome is associated with enhanced pERK activity, the repression of which can prevent craniofacial malformations. Proc. Natl. Acad. Sci. USA *106*, 15436–15441. https://doi.org/10.1073/pnas.0903302106.
46. Moriya, M., Inoue, S.-I., Miyagawa-Tomita, S., Nakashima, Y., Oba, D., Niihori, T., Hashi, M., Ohnishi, H., Kure, S., Matsubara, Y., and Aoki, Y. (2015). Adult mice expressing a Braf Q241R mutation on an ICR/CD-1 background exhibit a cardio-facio-cutaneous syndrome phenotype. Hum. Mol. Genet. *24*, 7349–7360. https://doi.org/10.1093/hmg/ddv435.
47. Oba, D., Inoue, S.i., Miyagawa-Tomita, S., Nakashima, Y., Niihori, T., Yamaguchi, S., Matsubara, Y., and Aoki, Y. (2018). Mice with an Oncogenic HRAS Mutation are Resistant to High-Fat Diet-Induced Obesity and Exhibit Impaired Hepatic Energy Homeostasis. EBioMedicine *27*, 138–150. https://doi.org/10.1016/j.ebiom.2017.11.029.
48. Sullivan, K., El-Hoss, J., Quinlan, K.G.R., Deo, N., Garton, F., Seto, J.T.C., Gdalevitch, M., Turner, N., Cooney, G.J., Kolanczyk, M., et al. (2014). NF1 is a critical regulator of muscle development and metabolism. Hum. Mol. Genet. *23*, 1250–1259. https://doi.org/10.1093/hmg/ddt515.
49. Botero, V., Stanhope, B.A., Brown, E.B., Grenci, E.C., Boto, T., Park, S.J., King, L.B., Murphy, K.R., Colodner, K.J., Walker, J.A., et al. (2021). Neurofibromin regulates metabolic rate via neuronal mechanisms in Drosophila. Nat. Commun. *12*, 4285. https://doi.org/10.1038/s41467-021-24505-x.
50. Siqueiros-Sanchez, M., Dai, E., McGhee, C.A., McNab, J.A., Raman, M.M., and Green, T. (2024). Impact of pathogenic variants of the Ras–mitogen-activated protein kinase pathway on major white matter tracts in the human brain. Brain Commun. *6*, fcae274. https://doi.org/10.1093/braincomms/fcae274.
51. Fattah, M., Raman, M.M., Reiss, A.L., and Green, T. (2021). PTPN11 Mutations in the Ras-MAPK Signaling Pathway Affect Human White Matter Microstructure. Cereb. Cortex *31*, 1489–1499. https://doi.org/10.1093/cercor/bhaa299.
52. Koini, M., Rombouts, S.A.R.B., Veer, I.M., Van Buchem, M.A., and Huijbregts, S.C.J. (2017). White matter microstructure of patients with neurofibromatosis type 1 and its relation to inhibitory control. Brain Imaging and Behavior *11*, 1731–1740. https://doi.org/10.1007/s11682-016-9641-3.
53. Cizmeci, M.N., Lequin, M., Lichtenbelt, K.D., Chitayat, D., Kannu, P., James, A.G., Groenendaal, F., Chakkarapani, E., Blaser, S., and de Vries, L.S. (2018). Characteristic MR Imaging Findings of the Neonatal Brain in RASopathies. AJNR. Am. J. Neuroradiol. *39*, 1146–1152. https://doi.org/10.3174/ajnr.A5611.
54. McGhee, C.A., Honari, H., Siqueiros-Sanchez, M., Serur, Y., van Staalduinen, E.K., Stevenson, D., Bruno, J.L., Raman, M.M., and Green, T. (2024). Influences of RASopathies on Neuroanatomical Variation in Children. Biol. Psychiatry Cogn. Neurosci. Neuroimaging *9*, 858–870. https://doi.org/10.1016/j.bpsc.2024.04.003.
55. Milacic, M., Beavers, D., Conley, P., Gong, C., Gillespie, M., Griss, J., Haw, R., Jassal, B., Matthews, L., May, B., et al. (2024). The Reactome Pathway Knowledgebase 2024. Nucleic Acids Res. *52*, D672–D678. https://doi.org/10.1093/nar/gkad1025.
56. Krug, K., Mertins, P., Zhang, B., Hornbeck, P., Raju, R., Ahmad, R., Szucs, M., Mundt, F., Forestier, D., Jane-Valbuena, J., et al. (2019). A Curated Resource for Phosphosite-specific Signature Analysis. Mol. Cell. Proteomics *18*, 576–593. https://doi.org/10.1074/mcp.TIR118.000943.
57. Li, X., Newbern, J.M., Wu, Y., Morgan-Smith, M., Zhong, J., Charron, J., and Snider, W.D. (2012). MEK Is a Key Regulator of Gliogenesis in the Developing Brain. Neuron *75*, 1035–1050. https://doi.org/10.1016/j.neuron.2012.08.031.
58. Li, S., Mattar, P., Dixit, R., Lawn, S.O., Wilkinson, G., Kinch, C., Eisenstat, D., Kurrasch, D.M., Chan, J.A., and Schuurmans, C. (2014). RAS/ERK Signaling Controls Proneural Genetic Programs in Cortical Development and Gliomagenesis. J. Neurosci. *34*, 2169–2190. https://doi.org/10.1523/JNEUROSCI.4077-13.2014.
59. Ishii, A., Fyffe-Maricich, S.L., Furusho, M., Miller, R.H., and Bansal, R. (2012). ERK1/ERK2 MAPK Signaling is Required to Increase Myelin Thickness Independent of Oligodendrocyte Differentiation and Initiation of Myelination. J. Neurosci. *32*, 8855–8864. https://doi.org/10.1523/JNEUROSCI.0137-12.2012.
60. Galabova-Kovacs, G., Catalanotti, F., Matzen, D., Reyes, G.X., Zezula, J., Herbst, R., Silva, A., Walter, I., and Baccarini, M. (2008). Essential role of B-Raf in oligodendrocyte maturation and myelination during postnatal central nervous system development. J. Cell Biol. *180*, 947–955. https://doi.org/10.1083/jcb.200709069.
61. Kang, M., and Lee, Y.-S. (2019). The impact of RASopathy-associated mutations on CNS development in mice and humans. Mol. Brain *12*, 96. https://molecularbrain.biomedcentral.com/articles/10.1186/s13041-019-0517-5.
62. Rizvi, T.A., Akunuru, S., de Courten-Myers, G., Switzer, R.C., Nordlund, M.L., and Ratner, N. (1999). Region-specific astrogliosis in brains of mice heterozygous for mutations in the neurofibromatosis type 1 (Nf1) tumor suppressor. Brain Res. *816*, 111–123. https://doi.org/10.1016/s0006-8993(98)01133-0.
63. Bennett, M.R., Rizvi, T.A., Karyala, S., McKinnon, R.D., and Ratner, N. (2003). Aberrant Growth and Differentiation of Oligodendrocyte Progenitors in Neurofibromatosis Type 1

Mutants. J. Neurosci. *23*, 7207–7217. https://doi.org/10.1523/JNEUROSCI.23-18-07207.2003.

64. López-Juárez, A., Titus, H.E., Silbak, S.H., Pressler, J.W., Rizvi, T.A., Bogard, M., Bennett, M.R., Ciraolo, G., Williams, M.T., Vorhees, C.V., and Ratner, N. (2017). Oligodendrocyte Nf1 Controls Aberrant Notch Activation and Regulates Myelin Structure and Behavior. Cell Rep. *19*, 545–557. https://doi.org/10.1016/j.celrep.2017.03.073.
65. Ehrman, L.A., Nardini, D., Ehrman, S., Rizvi, T.A., Gulick, J., Krenz, M., Dasgupta, B., Robbins, J., Ratner, N., Nakafuku, M., and Waclaw, R.R. (2014). The protein tyrosine phosphatase Shp2 is required for the generation of oligodendrocyte progenitor cells and myelination in the mouse telencephalon. J. Neurosci. *34*, 3767–3778. https://doi.org/10.1523/JNEUROSCI.3515-13.2014.
66. Pan, Y., Hysinger, J.D., Yalçın, B., Lennon, J.J., Byun, Y.G., Raghavan, P., Schindler, N.F., Anastasaki, C., Chatterjee, J., Ni, L., et al. (2024). Nf1 mutation disrupts activity-dependent oligodendroglial plasticity and motor learning in mice. Nat. Neurosci. *27*, 1555–1564. https://doi.org/10.1038/s41593-024-01654-y.
67. Angara, K., Pai, E.L.-L., Bilinovich, S.M., Stafford, A.M., Nguyen, J.T., Li, K.X., Paul, A., Rubenstein, J.L., and Vogt, D. (2020). Nf1 deletion results in depletion of the Lhx6 transcription factor and a specific loss of parvalbumin+ cortical interneurons. Proc. Natl. Acad. Sci. USA *117*, 6189–6195. https://doi.org/10.1073/pnas.1915458117.
68. Mayes, D.A., Rizvi, T.A., Titus-Mitchell, H., Oberst, R., Ciraolo, G.M., Vorhees, C.V., Robinson, A.P., Miller, S.D., Cancelas, J.A., Stemmer-Rachamimov, A.O., and Ratner, N. (2013). Nf1 loss and Ras hyperactivation in oligodendrocytes induce NOS-driven defects in myelin and vasculature. Cell Rep. *4*, 1197–1212. https://doi.org/10.1016/j.celrep.2013.08.011.
69. Titus, H.E., López-Juárez, A., Silbak, S.H., Rizvi, T.A., Bogard, M., and Ratner, N. (2017). Oligodendrocyte RasG12V expressed in its endogenous locus disrupts myelin structure through increased MAPK, nitric oxide, and notch signaling. Glia *65*, 1990–2002. https://doi.org/10.1002/glia.23209.
70. Costa, R.M., Federov, N.B., Kogan, J.H., Murphy, G.G., Stern, J., Ohno, M., Kucherlapati, R., Jacks, T., and Silva, A.J. (2002). Mechanism for the learning deficits in a mouse model of neurofibromatosis type 1. Nature *415*, 526–530. https://doi.org/10.1038/nature711.
71. Cui, Y., Costa, R.M., Murphy, G.G., Elgersma, Y., Zhu, Y., Gutmann, D.H., Parada, L.F., Mody, I., and Silva, A.J. (2008). Neurofibromin Regulation of ERK Signaling Modulates GABA Release and Learning. Cell *135*, 549–560. https://doi.org/10.1016/j.cell.2008.09.060.
72. Lee, Y.-S., Ehninger, D., Zhou, M., Oh, J.-Y., Kang, M., Kwak, C., Ryu, H.-H., Butz, D., Araki, T., Cai, Y., et al. (2014). Mechanism and treatment for learning and memory deficits in mouse models of Noonan syndrome. Nat. Neurosci. *17*, 1736–1743. https://doi.org/10.1038/nn.3863.
73. Li, W., Cui, Y., Kushner, S.A., Brown, R.A.M., Jentsch, J.D., Frankland, P.W., Cannon, T.D., and Silva, A.J. (2005). The HMG-CoA Reductase Inhibitor Lovastatin Reverses the Learning and Attention Deficits in a Mouse Model of Neurofibromatosis Type 1. Curr. Biol. *15*, 1961–1967. https://doi.org/10.1016/j.cub.2005.09.043.
74. Omrani, A., van der Vaart, T., Mientjes, E., van Woerden, G.M., Hojjati, M.R., Li, K.W., Gutmann, D.H., Levelt, C.N., Smit, A.B., Silva, A.J., et al. (2015). HCN channels are a novel therapeutic target for cognitive dysfunction in Neurofibromatosis type 1. Mol. Psychiatry *20*, 1311–1321. https://doi.org/10.1038/mp.2015.48.
75. Viosca, J., Schuhmacher, A.J., Guerra, C., and Barco, A. (2009). Germline expression of H-RasG12V causes neurological deficits associated to Costello syndrome. Gene Brain Behav. *8*, 60–71. https://doi.org/10.1111/j.1601-183X.2008.00443.x.
76. Vorhees, C.V., and Williams, M.T. (2006). Morris water maze: procedures for assessing spatial and related forms of learning and memory. Nat. Protoc. *1*, 848–858. https://doi.org/10.1038/nprot.2006.116.
77. Pride, N.A., Haebich, K.M., Walsh, K.S., Lami, F., Rouel, M., Maier, A., Chisholm, A.K., Lorenzo, J., Hearps, S.J.C., North, K.N., and Payne, J.M. (2023). Sensory Processing in Children and Adolescents with Neurofibromatosis Type 1. Cancers (Basel) *15*, 3612. https://doi.org/10.3390/cancers15143612.
78. Robinson, J.E., Coughlin, G.M., Hori, A.M., Cho, J.R., Mackey, E.D., Turan, Z., Patriarchi, T., Tian, L., and Gradinaru, V. (2019). Optical dopamine monitoring with dLight1 reveals mesolimbic phenotypes in a mouse model of neurofibromatosis type 1. eLife *8*, e48983. https://doi.org/10.7554/eLife.48983.
79. Yilmaz, M., and Meister, M. (2013). Rapid Innate Defensive Responses of Mice to Looming Visual Stimuli. Curr. Biol. *23*, 2011–2015. https://doi.org/10.1016/j.cub.2013.08.015.
80. Iroegbu, J.D., Ijomone, O.K., Femi-Akinlosotu, O.M., and Ijomone, O.M. (2021). ERK/MAPK signalling in the developing brain: Perturbations and consequences. Neurosci. Biobehav. Rev. *131*, 792–805. https://doi.org/10.1016/j.neubiorev.2021.10.009.
81. Pucilowska, J., Puzerey, P.A., Karlo, J.C., Galán, R.F., and Landreth, G.E. (2012). Disrupted ERK Signaling during Cortical Development Leads to Abnormal Progenitor Proliferation, Neuronal and Network Excitability and Behavior, Modeling Human Neuro-Cardio-Facial-Cutaneous and Related Syndromes. J. Neurosci. *32*, 8663–8677. https://doi.org/10.1523/JNEUROSCI.1107-12.2012.
82. Imamura, O., Satoh, Y., Endo, S., and Takishima, K. (2008). Analysis of Extracellular Signal-Regulated Kinase 2 Function in Neural Stem/Progenitor Cells via Nervous System-Specific Gene Disruption. Stem Cell. *26*, 3247–3256. https://doi.org/10.1634/stemcells.2008-0578.
83. Imamura, O., Pagès, G., Pouysségur, J., Endo, S., and Takishima, K. (2010). ERK1 and ERK2 are required for radial glial maintenance and cortical lamination. Genes Cells *15*, 1072–1088. https://doi.org/10.1111/j.1365-2443.2010.01444.x.
84. Samuels, I.S., Karlo, J.C., Faruzzi, A.N., Pickering, K., Herrup, K., Sweatt, J.D., Saitta, S.C., and Landreth, G.E. (2008). Deletion of ERK2 Mitogen-Activated Protein Kinase Identifies Its Key Roles in Cortical Neurogenesis and Cognitive Function. J. Neurosci. *28*, 6983–6995. https://doi.org/10.1523/JNEUROSCI.0679-08.2008.
85. Bajenaru, M.L., Zhu, Y., Hedrick, N.M., Donahoe, J., Parada, L.F., and Gutmann, D.H. (2002). Astrocyte-specific inactivation of the neurofibromatosis 1 gene (NF1) is insufficient for astrocytoma formation. Mol. Cell Biol. *22*, 5100–5113. https://doi.org/10.1128/MCB.22.14.5100-5113.2002.

86. Wagle, M.-C., Kirouac, D., Klijn, C., Liu, B., Mahajan, S., Junttila, M., Moffat, J., Merchant, M., Huw, L., Wongchenko, M., et al. (2018). A transcriptional MAPK Pathway Activity Score (MPAS) is a clinically relevant biomarker in multiple cancer types. npj Precis. Oncol. *2*, 7. https://doi.org/10.1038/s41698-018-0051-4.
87. Breunig, J.J., Levy, R., Antonuk, C.D., Molina, J., Dutra-Clarke, M., Park, H., Akhtar, A.A., Kim, G.B., Town, T., Hu, X., et al. (2015). Ets Factors Regulate Neural Stem Cell Depletion and Gliogenesis in Ras Pathway Glioma. Cell Rep. *12*, 258–271. https://doi.org/10.1016/j.celrep.2015.06.012.
88. Fontanet, P.A., Ríos, A.S., Alsina, F.C., Paratcha, G., and Ledda, F. (2018). Pea3 Transcription Factors, Etv4 and Etv5, Are Required for Proper Hippocampal Dendrite Development and Plasticity. Cereb. Cortex *28*, 236–249. https://doi.org/10.1093/cercor/bhw372.
89. Newton, K., Dugger, D.L., Sengupta-Ghosh, A., Ferrando, R.E., Chu, F., Tao, J., Lam, W., Haller, S., Chan, S., Sa, S., et al. (2018). Ubiquitin ligase COP1 coordinates transcriptional programs that control cell type specification in the developing mouse brain. Proc. Natl. Acad. Sci. USA *115*, 11244–11249. https://doi.org/10.1073/pnas.1805033115.
90. Liu, Y., and Zhang, Y. (2019). ETV5 is Essential for Neuronal Differentiation of Human Neural Progenitor Cells by Repressing NEUROG2 Expression. Stem Cell Rev. Rep. *15*, 703–716. https://doi.org/10.1007/s12015-019-09904-4.
91. Shih, H.-Y., Chen, H.-Y., Huang, Y.-C., Yeh, T.-H., Chen, Y.-C., and Cheng, Y.-C. (2023). Etv5a Suppresses Neural Progenitor Cell Proliferation by Inhibiting sox2 Transcription. Stem Cells Dev. *32*, 524–538. https://doi.org/10.1089/scd.2023.0005.
92. Asleh, J., Shofty, B., Cohen, N., Kavushansky, A., López-Juárez, A., Constantini, S., Ratner, N., and Kahn, I. (2020). Brain-wide structural and functional disruption in mice with oligodendrocyte-specific Nf1 deletion is rescued by inhibition of nitric oxide synthase. Proc. Natl. Acad. Sci. USA *117*, 22506–22513. https://doi.org/10.1073/pnas.2008391117.
93. de Blank, P.M.K., Gross, A.M., Akshintala, S., Blakeley, J.O., Bollag, G., Cannon, A., Dombi, E., Fangusaro, J., Gelb, B.D., Hargrave, D., et al. (2022). MEK inhibitors for neurofibromatosis type 1 manifestations: Clinical evidence and consensus. Neuro Oncol. *24*, 1845–1856. https://doi.org/10.1093/neuonc/noac165.
94. Schreiber, J., Grimbergen, L.-A., Overwater, I., Vaart, T.v.d., Stedehouder, J., Schuhmacher, A.J., Guerra, C., Kushner, S.A., Jaarsma, D., and Elgersma, Y. (2017). Mechanisms underlying cognitive deficits in a mouse model for Costello Syndrome are distinct from other RASopathy mouse models. Sci. Rep. *7*, 1256. https://doi.org/10.1038/s41598-017-01218-0.
95. Silva, A.J., Frankland, P.W., Marowitz, Z., Friedman, E., Lazlo, G., Cioffi, D., Jacks, T., and Bourtchuladze, R. (1997). A mouse model for the learning and memory deficits associated with neurofibromatosis type I. Nat. Genet. *15*, 281–284. https://doi.org/10.1038/ng0397-281.
96. D'Archangel, M.A., Del Tufo, S.N., Cutting, L.E., Harrison, F.E., Ess, K.C., and Barquero, L.A. (2022). Virtual Reality Water Maze Navigation in Children with Neurofibromatosis Type 1 and Reading Disability: an Exploratory Study. J. Pediatr. Neuropsychol. *8*, 155–167. https://doi.org/10.1007/s40817-022-00132-2.
97. Pieribone, V.A., Shupliakov, O., Brodin, L., Hilfiker-Rothenfluh, S., Czernik, A.J., and Greengard, P. (1995). Distinct pools of synaptic vesicles in neurotransmitter release. Nature *375*, 493–497. https://doi.org/10.1038/375493a0.
98. Gómez-Pinilla, F., So, V., and Kesslak, J.P. (2001). Spatial learning induces neurotrophin receptor and synapsin I in the hippocampus. Brain Res. *904*, 13–19. https://doi.org/10.1016/S0006-8993(01)02394-0.
99. English, J.D., and Sweatt, J.D. (1997). A Requirement for the Mitogen-activated Protein Kinase Cascade in Hippocampal Long Term Potentiation. J. Biol. Chem. *272*, 19103–19106. https://doi.org/10.1074/jbc.272.31.19103.
100. Atkins, C.M., Selcher, J.C., Petraitis, J.J., Trzaskos, J.M., and Sweatt, J.D. (1998). The MAPK cascade is required for mammalian associative learning. Nat. Neurosci. *1*, 602–609. https://doi.org/10.1038/2836.
101. Winder, D.G., Martin, K.C., Muzzio, I.A., Rohrer, D., Chruscinski, A., Kobilka, B., and Kandel, E.R. (1999). ERK Plays a Regulatory Role in Induction of LTP by Theta Frequency Stimulation and Its Modulation by β-Adrenergic Receptors. Neuron *24*, 715–726. https://doi.org/10.1016/S0896-6273(00)81124-1.
102. Giovannini, M.G., Blitzer, R.D., Wong, T., Asoma, K., Tsokas, P., Morrison, J.H., Iyengar, R., and Landau, E.M. (2001). Mitogen-Activated Protein Kinase Regulates Early Phosphorylation and Delayed Expression of Ca^{2+}/Calmodulin-Dependent Protein Kinase II in Long-Term Potentiation. J. Neurosci. *21*, 7053–7062. https://doi.org/10.1523/JNEUROSCI.21-18-07053.2001.
103. Patterson, M.A., Szatmari, E.M., and Yasuda, R. (2010). AMPA receptors are exocytosed in stimulated spines and adjacent dendrites in a Ras-ERK–dependent manner during long-term potentiation. Proc. Natl. Acad. Sci. USA *107*, 15951–15956. https://doi.org/10.1073/pnas.0913875107.
104. Zhu, J.J., Qin, Y., Zhao, M., Van Aelst, L., and Malinow, R. (2002). Ras and Rap Control AMPA Receptor Trafficking during Synaptic Plasticity. Cell *110*, 443–455. https://doi.org/10.1016/S0092-8674(02)00897-8.
105. Oliveira, A.F., and Yasuda, R. (2014). Neurofibromin Is the Major Ras Inactivator in Dendritic Spines. J. Neurosci. *34*, 776–783. https://doi.org/10.1523/JNEUROSCI.3096-13.2014.
106. Branco, T., and Redgrave, P. (2020). The Neural Basis of Escape Behavior in Vertebrates. Annu. Rev. Neurosci. *43*, 417–439. https://doi.org/10.1146/annurev-neuro-100219-122527.
107. Basso, M.A., Bickford, M.E., and Cang, J. (2021). Unraveling circuits of visual perception and cognition through the superior colliculus. Neuron *109*, 918–937. https://doi.org/10.1016/j.neuron.2021.01.013.
108. Evans, D.A., Stempel, A.V., Vale, R., Ruehle, S., Lefler, Y., and Branco, T. (2018). A synaptic threshold mechanism for computing escape decisions. Nature *558*, 590–594. https://doi.org/10.1038/s41586-018-0244-6.
109. Zhou, Z., Liu, X., Chen, S., Zhang, Z., Liu, Y., Montardy, Q., Tang, Y., Wei, P., Liu, N., Li, L., et al. (2019). A VTA GABAergic Neural Circuit Mediates Visually Evoked Innate Defensive Responses. Neuron *103*, 473–488.e6. https://doi.org/10.1016/j.neuron.2019.05.027.
110. Campagner, D., Vale, R., Tan, Y.L., Iordanidou, P., Pavón Arocas, O., Claudi, F., Stempel, A.V., Keshavarzi, S., Petersen, R.S., Margrie, T.W., and Branco, T. (2023). A

cortico-collicular circuit for orienting to shelter during escape. Nature *613*, 111–119. https://doi.org/10.1038/s41586-022-05553-9.

111. Zhao, X., Liu, M., and Cang, J. (2014). Visual cortex modulates the magnitude but not the selectivity of looming-evoked responses in the superior colliculus of awake mice. Neuron *84*, 202–213. https://doi.org/10.1016/j.neuron.2014.08.037.

112. Ritter, A., Habusha, S., Givon, L., Edut, S., and Klavir, O. (2024). Prefrontal control of superior colliculus modulates innate escape behavior following adversity. Nat. Commun. *15*, 2158. https://doi.org/10.1038/s41467-024-46460-z.

HGGA, Volume 7

Supplemental information

Genetic activation of ERK2 recapitulates core neurodevelopmental features of Rasopathy syndromes in mice

Kassidy E. Grover, Zoe R. Cappel, Avery M. Volz, Evelin M. Cotella, Kelly Smallwood, Christine A. Berryhill, Kimaya R. Raje, Austen A. Fisher, Mary Claire T. Casper, Diana Nardini, Tilat A. Rizvi, Rosa M. Salazar, Ashley Wooten, Michael T. Williams, Charles V. Vorhees, Lindsey E. Romick, Kenneth D. Greis, Yueh-Chiang Hu, Linde A. Miles, Steven P. Angus, Nancy Ratner, Carlos E. Prada, K. Nicole Weaver, Ronald R. Waclaw, and J. Elliott Robinson

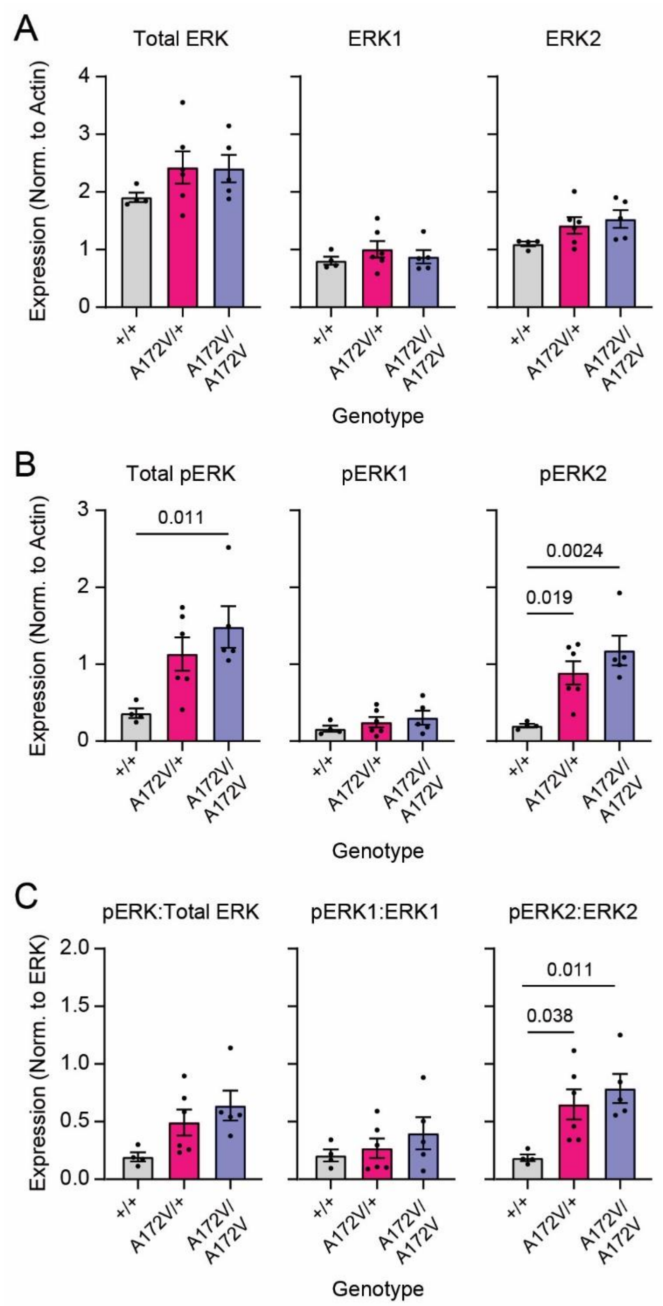

Figure S1. Analysis of ERK phosphorylation in PD7 livers in *Mapk1* mutant and control mice. (A) There were no significant genotypic differences in total ERK (*left*; $n_{+/+}$ = 4; $n_{A172V/+}$ = 6; $n_{A172V/A172V}$ = 5; one-way ANOVA; $F_{2,12}$ = 1.28, p = 0.31), ERK1 (*middle*; $n_{+/+}$ = 4; $n_{A172V/+}$ = 6; $n_{A172V/A172V}$ = 5; one-way ANOVA; $F_{2,12}$ = 0.65, p = 0.54), or ERK2 (right; $n_{+/+}$ = 4; $n_{A172V/+}$ = 6; $n_{A172V/A172V}$ = 5; one-way ANOVA; $F_{2,12}$ = 2.37, p = 0.14) in PD7 liver samples, as measured by

Western blot. **(B)** There was a significant increase in total pERK expression in PD7 *Mapk1* mutant livers (*left*; $n_{+/+}$ = 4; $n_{A172V/+}$ = 6; $n_{A172V/A172V}$ = 5; one-way ANOVA; $F_{2,12}$ = 5.95, p = 0.016). Bonferroni *post hoc* analysis showed that pERK was significantly lower in *Mapk1*$^{+/+}$ samples relative to *Mapk1*$^{A172V/A172}$ samples (p = 0.011); this difference did not reach statistical significance in *Mapk1*$^{A172V/+}$ livers relative to control samples (p = 0.064). There were no genotypic differences in pERK1 (*middle*; $n_{+/+}$ = 4; $n_{A172V/+}$ = 6; $n_{A172V/A172V}$ = 5; one-way ANOVA; $F_{2,12}$ = 0.85, p = 0.45). pERK2 levels were higher in *Mapk1* mutant livers relative to control samples (*right*; $n_{+/+}$ = 4; $n_{A172V/+}$ = 6; $n_{A172V/A172V}$ = 5; one-way ANOVA; $F_{2,12}$ = 9.18, p = 0.0038). Bonferroni *post hoc* tests showed a significant increase in heterozygous mutants (p = 0.019) and homozygous mutants (p = 0.0024). **(C)** There were no genotypic differences in the pERK:total ERK ratio (*left*; $n_{+/+}$ = 4; $n_{A172V/+}$ = 6; $n_{A172V/A172V}$ = 5; one-way ANOVA; $F_{2,12}$ = 3.65, p = 0.058) or pERK1:ERK1 ratio (*middle*; $n_{+/+}$ = 4; $n_{A172V/+}$ = 6; $n_{A172V/A172V}$ = 5; one-way ANOVA; $F_{2,12}$ = 0.84, p = 0.46). The pERK2:ERK2 ratio was significantly increased in *Mapk1* mutants relative to wildtype controls (*right*; $n_{+/+}$ = 4; $n_{A172V/+}$ = 6; $n_{A172V/A172V}$ = 5; one-way ANOVA; $F_{2,12}$ = 6.17, p = 0.014). pERK2:ERK2 was higher in *Mapk1*$^{A172V/+}$ (Bonferroni *post hoc* test; p = 0.038) and *Mapk1*$^{A172V/A172V}$ livers (Bonferroni *post hoc* test; p = 0.011) relative to *Mapk1*$^{+/+}$ samples. Data is presented as mean ± SEM.

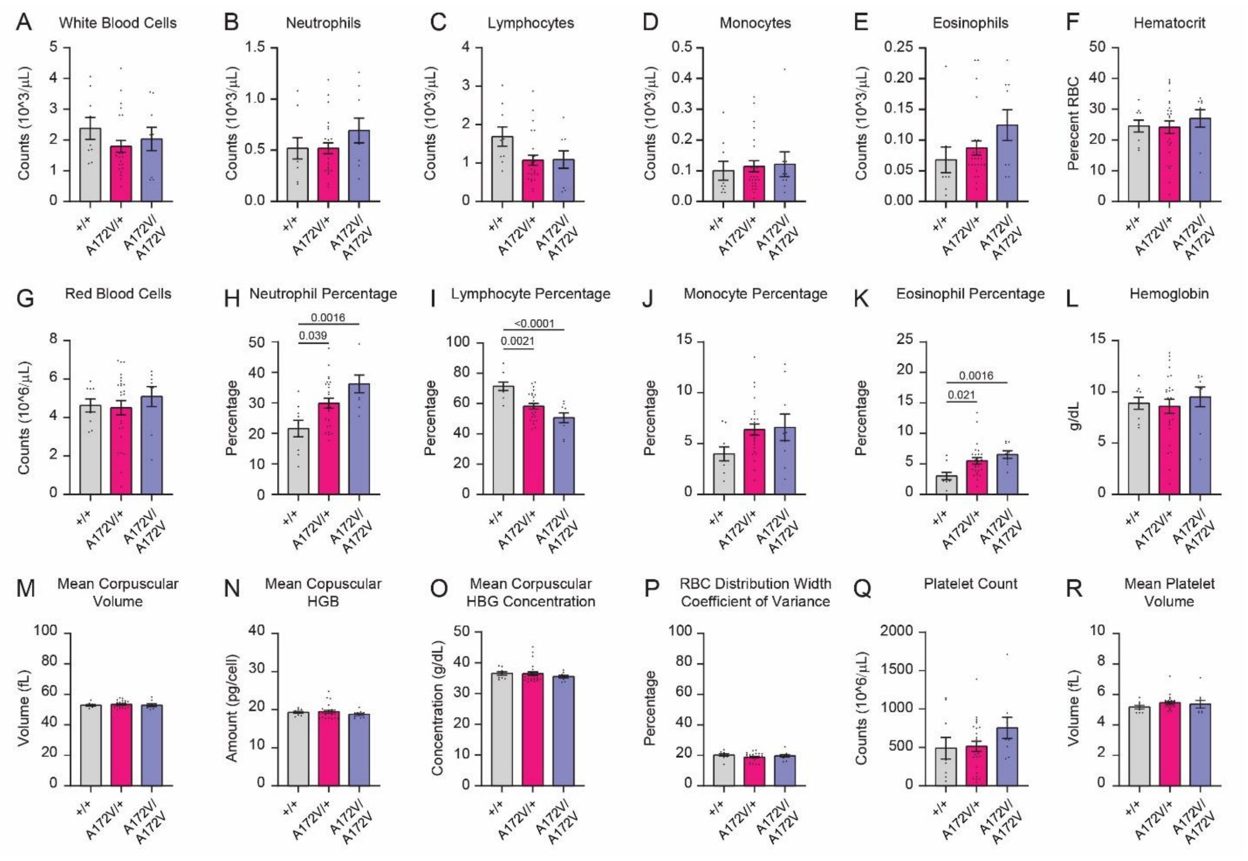

Figure S2. Hematological testing in *Mapk1* mutant and control mice. **(A)** At two weeks of age, there were no significant differences in white blood cell (WBC) counts between in *Mapk1* mutant and control mice ($n_{+/+}$ = 9; $n_{A172V/+}$ = 25; $n_{A172V/A172V}$ = 9; one-way ANOVA; $F_{2,40}$ = 0.45, p = 0.35). **(B-E)** There were no genotypic differences in absolute neutrophil ($n_{+/+}$ = 9; $n_{A172V/+}$ = 25; $n_{A172V/A172V}$ = 9; one-way ANOVA; $F_{2,40}$ = 0.61, p = 0.31), lymphocyte ($n_{+/+}$ = 9; $n_{A172V/+}$ = 25; $n_{A172V/A172V}$ = 9; one-way ANOVA; $F_{2,40}$ = 0.27, p = 0.069), monocyte ($n_{+/+}$ = 9; $n_{A172V/+}$ = 25; $n_{A172V/A172V}$ = 9; Kruskal-Wallis test; H_3 = 1.26, p = 0.53), or eosinophil counts ($n_{+/+}$ = 9; $n_{A172V/+}$ = 25; $n_{A172V/A172V}$ = 9; Kruskal-Wallis test; H_3 = 2.82, p = 0.24). **(F-G)** There were no genotypic differences in hematocrit ($n_{+/+}$ = 9; $n_{A172V/+}$ = 25; $n_{A172V/A172V}$ = 9; one-way ANOVA; $F_{2,40}$ = 0.33, p = 0.72) or red blood cell (RBC) count ($n_{+/+}$ = 9; $n_{A172V/+}$ = 25; $n_{A172V/A172V}$ = 9; one-way ANOVA; $F_{2,40}$ = 0.41, p = 0.67). **(H)** There was a significant increase in the percentage of neutrophils in *Mapk1* mutants ($n_{+/+}$ = 9; $n_{A172V/+}$ = 25; $n_{A172V/A172V}$ = 9; one-way ANOVA; $F_{2,40}$ = 7.21, p = 0.0021).

Bonferroni *post hoc* analysis revealed a significant increase in both *Mapk1*$^{\mathrm{A172V/+}}$ (p = 0.039) and *Mapk1*$^{\mathrm{A172V/A172}}$ (p = 0.0016) neutrophil percentage compared to wild-type littermates. **(I)** There was a significant decrease in the percentage of lymphocytes ($n_{+/+}$ = 9; $n_{\mathrm{A172V/+}}$ = 25; $n_{\mathrm{A172V/A172V}}$ = 9; one-way ANOVA; $F_{2,40}$ = 11.87, p < 0.0001) in *Mapk1*$^{\mathrm{A172V/+}}$ (Bonferroni *post hoc* test; p = 0.0021) and *Mapk1*$^{\mathrm{A172V/A172V}}$ (Bonferroni *post hoc* test; p < 0.0001) mice. **(J)** There was no genotypic difference in monocyte percentage ($n_{+/+}$ = 9; $n_{\mathrm{A172V/+}}$ = 25; $n_{\mathrm{A172V/A172V}}$ = 9; one-way ANOVA; $F_{2,40}$ = 2.52, p = 0.093). **(K)** There was a significant increase in the percentage of eosinophils ($n_{+/+}$ = 9; $n_{\mathrm{A172V/+}}$ = 25; $n_{\mathrm{A172V/A172V}}$ = 9; Kruskal-Wallis test; H_3 = 12.57, p = 0.0019) in both *Mapk1*$^{\mathrm{A172V/+}}$ mice (Dunn's *post hoc* test; p = 0.021) and *Mapk1*$^{A172V/A172V}$ mice (Dunn's *post hoc* test; p = 0.0016). **(L-O)** There were no genotypic differences in hemoglobin (HGB) concentration ($n_{+/+}$ = 9; $n_{\mathrm{A172V/+}}$ = 25; $n_{\mathrm{A172V/A172V}}$ = 9; one-way ANOVA; $F_{2,40}$ = 0.30, p = 0.74), mean corpuscular volume ($n_{+/+}$ = 9; $n_{\mathrm{A172V/+}}$ = 25; $n_{\mathrm{A172V/A172V}}$ = 9; one-way ANOVA; $F_{2,40}$ = 0.38, p = 0.68), mean corpuscular HGB ($n_{+/+}$ = 9; $n_{\mathrm{A172V/+}}$ = 25; $n_{\mathrm{A172V/A172V}}$ = 9; Kruskal-Wallis test; H_3 = 1.24, p = 0.54) or corpuscular HBG concentration ($n_{+/+}$ = 9; $n_{\mathrm{A172V/+}}$ = 25; $n_{\mathrm{A172V/A172V}}$ = 9; Kruskal-Wallis test; H_3 = 1.58, p = 0.45). **(P)** There were no genotypic differences in the coefficient of variation of RBC distribution width ($n_{+/+}$ = 9; $n_{\mathrm{A172V/+}}$ = 25; $n_{\mathrm{A172V/A172V}}$ = 9; one-way ANOVA; $F_{2,40}$ = 0.89, p = 0.42). **(Q-R)** There were no genotypic differences in platelet count ($n_{+/+}$ = 9; $n_{\mathrm{A172V/+}}$ = 25; $n_{\mathrm{A172V/A172V}}$ = 9; one-way ANOVA; $F_{2,40}$ = 1.60, p = 0.22) or average volume ($n_{+/+}$ = 9; $n_{\mathrm{A172V/+}}$ = 25; $n_{\mathrm{A172V/A172V}}$ = 9; Kruskal-Wallis test; H_3 = 3.82, p = 0.15). Data is presented as mean ± SEM.

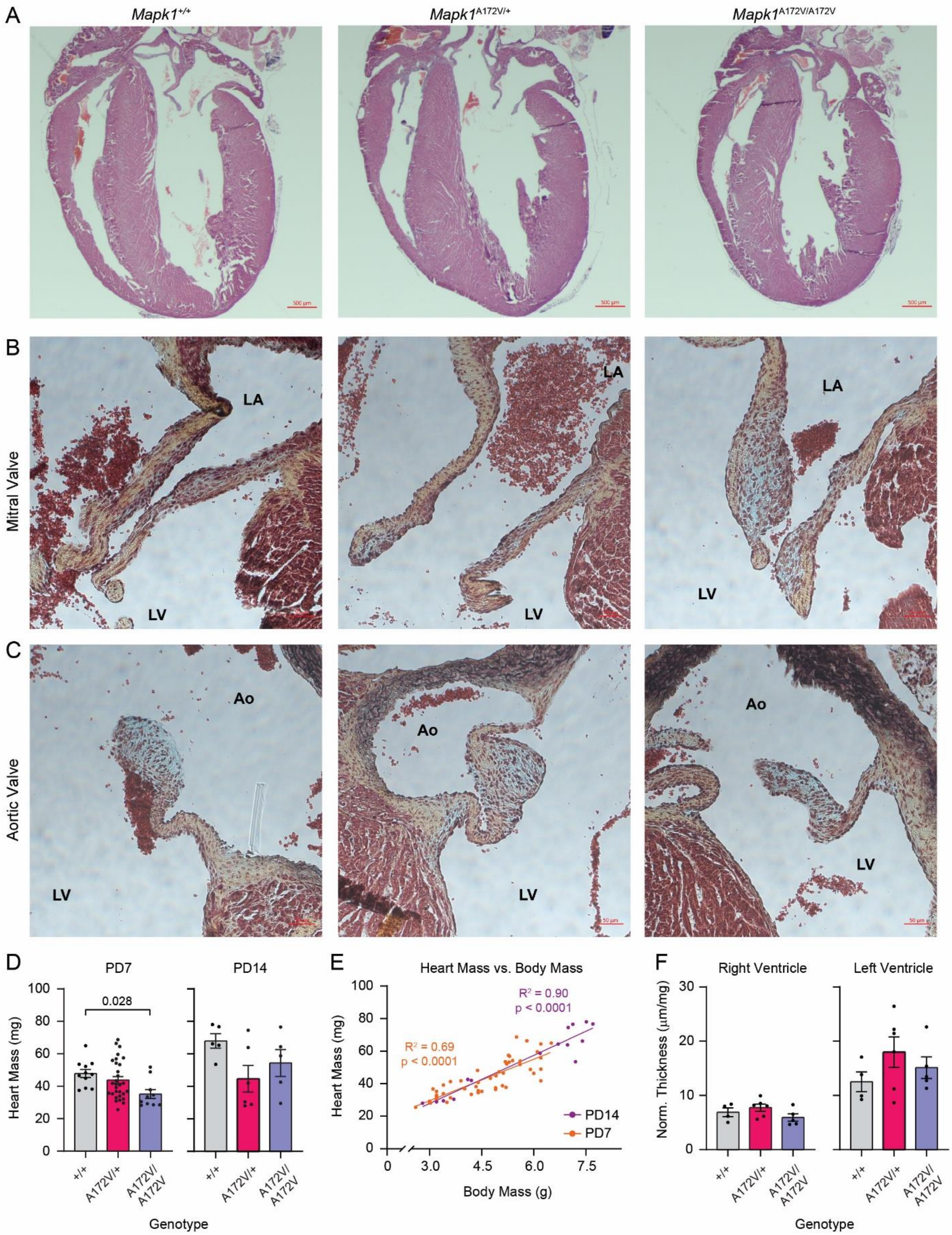

A
Mapk1+/+
Mapk1A172V/+
Mapk1A172V/A172V
B
Mitral Valve
LA
LV
C
Aortic Valve
Ao
LV
D
PD7
PD14
Heart Mass (mg)
0.028
+/+
A172V/+
A172V/ A172V
Genotype
E
Heart Mass vs. Body Mass
R2 = 0.90
p < 0.0001
R2 = 0.69
p < 0.0001
PD14
PD7
Heart Mass (mg)
Body Mass (g)
F
Right Ventricle
Left Ventricle
Norm. Thickness (μm/mg)
Genotype

55

Figure S3. Myocardial and valvular analysis of *Mapk1* mutant and control hearts. (A-C) Representative cross-sectional images of the myocardium (**A**), mitral valve (**B**), and aortic valve **(C)** from 2-week-old *Mapk1*$^{+/+}$ (*left*), *Mapk1*$^{A172V/+}$ (*middle*), and *Mapk1*$^{A172V/A172V}$ mice (*right*) hearts. **(D)** There was a significant difference in the heart weight of *Mapk1* mice at PD7 (*left*; $n_{+/+}$ = 11, $n_{A172V/+}$ = 30, $n_{A172V/A172V}$ = 10; one-way ANOVA; $F_{2,48}$ = 3.89, p = 0.027) with a significant reduction in *Mapk1*$^{A172V/A172V}$ heart mass compared to wild-type littermates (Bonferroni *post hoc* test; p = 0.028). Genotypic differences in heart mass at PD14 did not reach statistical significance (*right*; $n_{+/+}$ = 5, $n_{A172V/+}$ = 6, $n_{A172V/A172V}$ = 5; one-way ANOVA; $F_{2,13}$ = 2.57, p = 0.11). **(E)** There was a significant correlation between heart mass and body mass at PD7 ($R^2(49)$ = 0.69, p < 0.0001) and PD14 ($R^2(13)$ = 0.90, p < 0.0001). **(F)** There was no significant change in normalized right ventricular (one-way ANOVA; $F_{2,12}$ = 1.82, p = 0.20) or left ventricular thickness (one-way ANOVA; $F_{2,12}$ = 1.25, p = 0.32) between genotypes. Scale bar for **A** is 500 μm, and the scale bars for **B-C** are 50 μm. Data is presented as mean ± SEM.

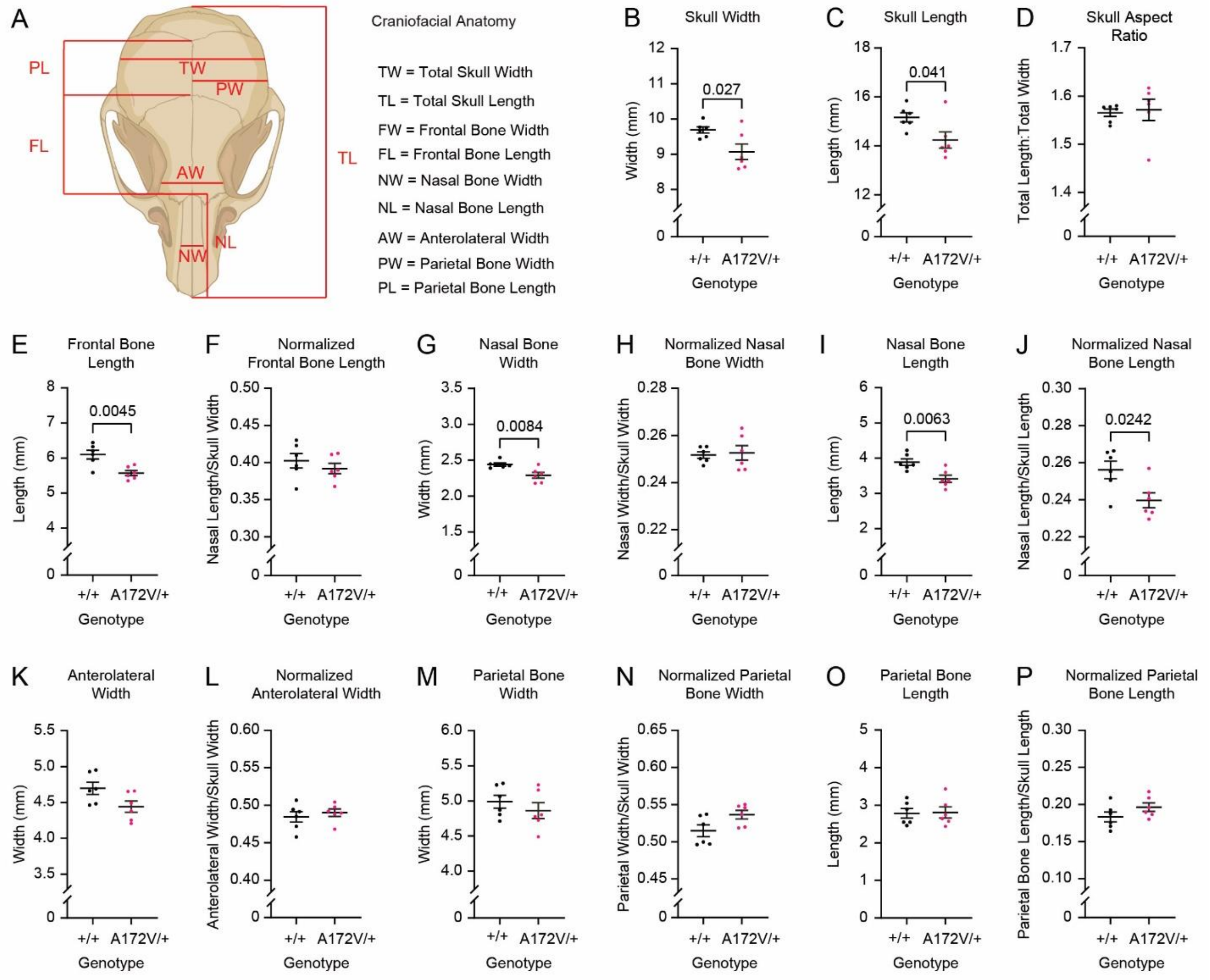

Figure S4. Craniofacial analysis in *Mapk1* mutant and control mice. (A) Schematic showing skull features measures in PD7 *Mapk1*$^{+/+}$ and *Mapk1*$^{A172V/+}$ mice. **(B-C)** There was a significant reduction in the overall width ($n_{+/+} = 6$; $n_{A172V/+} = 6$; unpaired t-test; $t_{10} = 2.59$, $p = 0.027$) and length of skulls in heterozygous mutant mice (Mann-Whitney U-test; $U(n_{+/+} = 6, n_{A172V/+} = 6) = 5$, $p = 0.041$). **(D)** There was no difference in the skull aspect ratio (length:width) between genotypes (Mann-Whitney U-test; $U(n_{+/+} = 6, n_{A172V/+} = 6) = 10$, $p = 0.24$). **(E-F)** There was a significant reduction in frontal bone length in *Mapk1*$^{A172V/+}$ mice ($n_{+/+} = 6$; $n_{A172V/+} = 6$; unpaired t-test; $t_{10} = 3.65$, $p = 0.0045$) that was not significant after normalizing to the total skull length ($n_{+/+} = 6$; $n_{A172V/+} = 6$; unpaired t-test; $t_{10} = 0.88$, $p = 0.40$). **(G-H)** There was a significant reduction in nasal bone

width ($n_{+/+}$ = 6; $n_{A172V/+}$ = 6; unpaired t-test; t_{10}= 3.27, p = 0.0084) in *Mapk1*$^{A172V/+}$ mice that was not significant after normalizing to the overall skull width ($n_{+/+}$ = 6; $n_{A172V/+}$ = 6; unpaired t-test; t_{10}= 0.27, p = 0.80). **(I-J)** There was a significant decrease in the nasal bone length in *Mapk1*$^{A172V/+}$ mice compared to wildtype littermates ($n_{+/+}$ = 6; $n_{A172V/+}$ = 6; unpaired t-test; t_{10}= 3.44, p = 0.0063) that persisted after it was normalized to the total skull length ($n_{+/+}$ = 6; $n_{A172V/+}$ = 6; unpaired t-test; t_{10}= 2.65, p = 0.024). **(K-P)** There were no significant differences in the skull anterolateral width ($n_{+/+}$ = 6; $n_{A172V/+}$ = 6; unpaired t-test; t_{10}= 2.19, p = 0.054), normalized anterolateral width ($n_{+/+}$ = 6; $n_{A172V/+}$ = 6; unpaired t-test; t_{10}= 0.63, p = 0.54), parietal bone width ($n_{+/+}$ = 6; $n_{A172V/+}$ = 6; unpaired t-test; t_{10}= 0.86, p = 0.41), normalized parietal bone width (Mann-Whitney U-test; U($n_{+/+}$ = 6, $n_{A172V/+}$ = 6) = 7, p = 0.093), parietal bone length (unpaired t-test; t_{10}= 0.11, p = 0.92), and normalized parietal bone length ($n_{+/+}$ = 6; $n_{A172V/+}$ = 6; unpaired t-test; t_{10}= 1.48, p = 0.17) between *Mapk1*$^{A172V/+}$ and *Mapk1*$^{+/+}$ mice. Data is presented as mean ± SEM.

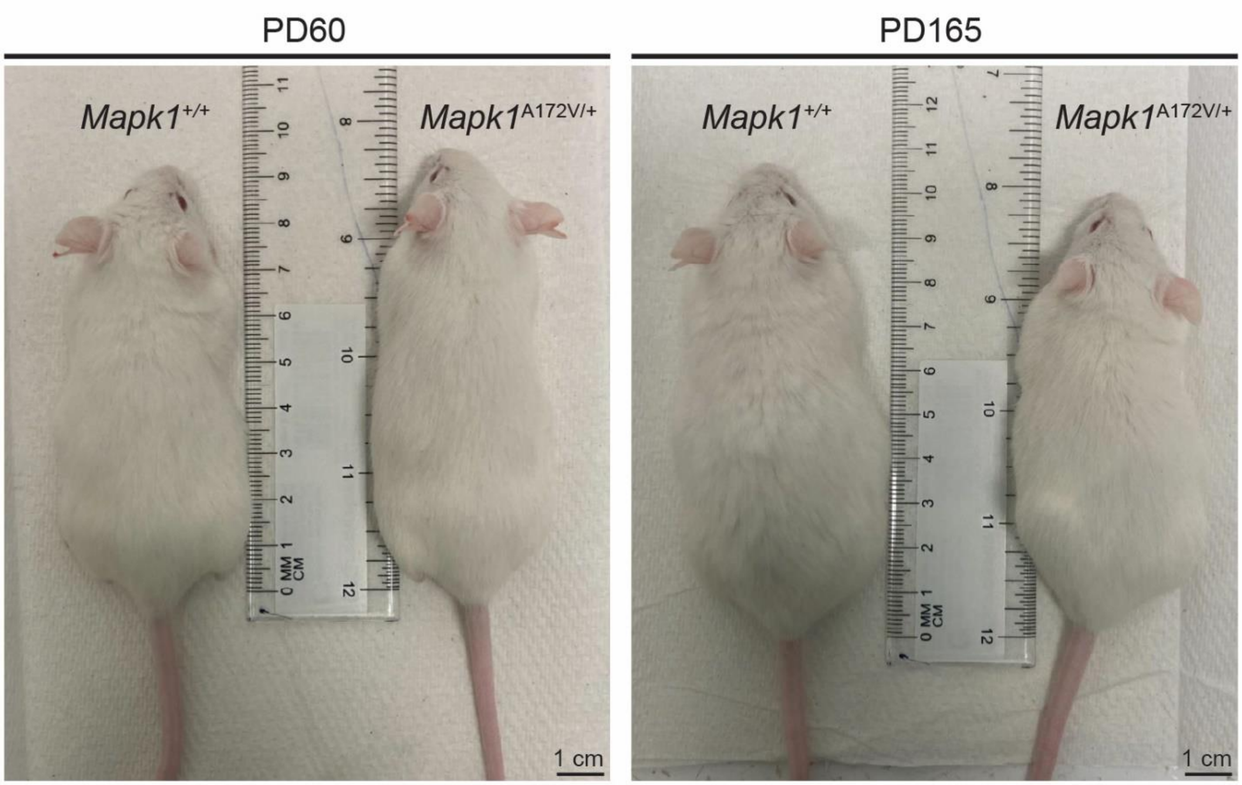

Figure S5. Representative images showing male *Mapk1*$^{+/+}$ and *Mapk1*$^{A172V/+}$ mice at PD60 (*left*) and PD165 (*right*). Scale bar is 1cm.

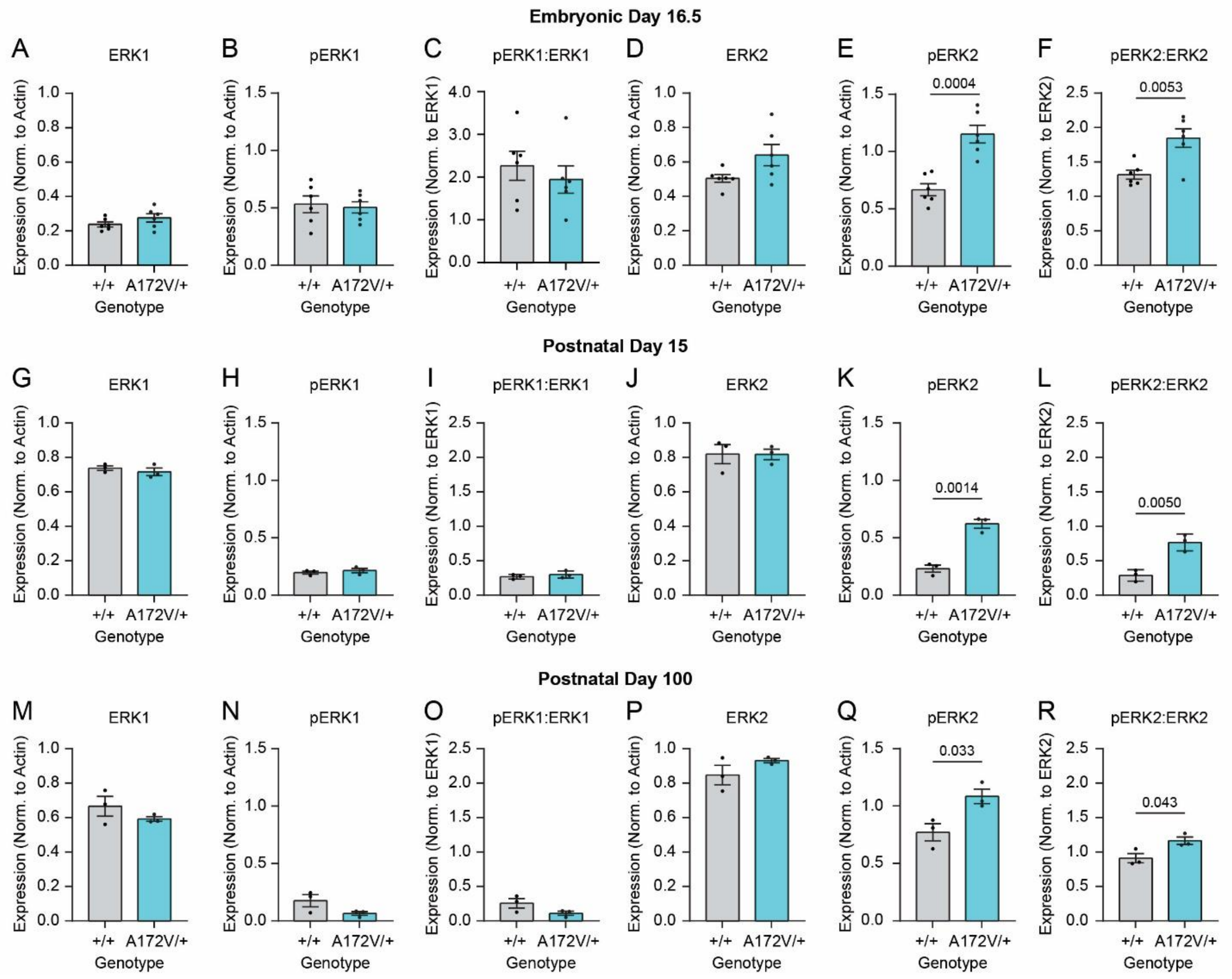

Figure S6. Developmental time course of ERK phosphorylation in the brains of *Mapk1* mutant and control mice. (A-D) At ED16.5, there were no genotypic differences in the level of ERK1 ($n_{+/+}$ = 6; $n_{A172V/+}$ = 6; unpaired t-test; t_{10} = 1.33, p = 0.21), pERK1 ($n_{+/+}$ = 6; $n_{A172V/+}$ = 6; unpaired t-test; t_{10} = 0.305, p = 0.77), the pERK1:ERK1 ratio ($n_{+/+}$ = 6; $n_{A172V/+}$ = 6; unpaired t-test; t_{10} = 0.69, p = 0.51), or total ERK2 ($n_{+/+}$ = 6; $n_{A172V/+}$ = 6; unpaired t-test with Welch's correction; t_{10} = 2.073, p = 0.082) in forebrain samples, as measured by Western blot. **(E-F)** There were significant increases in pERK2 ($n_{+/+}$ = 6; $n_{A172V/+}$ = 6; unpaired t-test; t_{10} = 5.18, p = 0.0004) and the pERK2:ERK2 ratio ($n_{+/+}$ = 6; $n_{A172V/+}$ = 6; unpaired t-test; t_{10} = 3.55, p = 0.0053) in *Mapk1*$^{A172V/+}$ forebrain samples relative to controls at ED16.5. **(G-J)** At PD15, there were no significant genotypic differences in the abundance of ERK1 ($n_{+/+}$ = 3; $n_{A172V/+}$ = 3; unpaired t-test; t_4 = 0.83, p

= 0.45), pERK1 ($n_{+/+}$ = 3; $n_{A172V/+}$ = 3; unpaired t-test; t_4 = 0.72, p = 0.51), pERK1:ERK1 ($n_{+/+}$ = 3; $n_{A172V/+}$ = 3; unpaired t-test; t_4 = 0.89, p = 0.42), and ERK2 ($n_{+/+}$ = 3; $n_{A172V/+}$ = 3; unpaired t-test; t_4 = 0.025, p = 0.98) in forebrain samples. **(K-L)** Significant increases in pERK2 ($n_{+/+}$ = 3; $n_{A172V/+}$ = 3; unpaired t-test; t_4 = 7.88, p = 0.0014) and pERK2:ERK2 ($n_{+/+}$ = 3; $n_{A172V/+}$ = 3; unpaired t-test; t_4 = 5.58, p = 0.005) were observed in *Mapk1*$^{A172V/+}$ forebrain samples at PD15. **(M-P)** At PD100, there were no genotypic differences in ERK1 ($n_{+/+}$ = 3; $n_{A172V/+}$ = 3; unpaired t-test; t_4 = 1.26, p = 0.28), pERK1 ($n_{+/+}$ = 3; $n_{A172V/+}$ = 3; unpaired t-test; t_4 = 1.99, p = 0.12), pERK1:ERK1 ($n_{+/+}$ = 3; $n_{A172V/+}$ = 3; unpaired t-test; t_4 = 1.93, p = 0.13), or ERK2 ($n_{+/+}$ = 3; $n_{A172V/+}$ = 3; unpaired t-test; t_4 = 1.47, p = 0.22) in forebrain samples. Significant increases in pERK2 ($n_{+/+}$ = 3; $n_{A172V/+}$ = 3; unpaired t-test; t_4 = 3.204, p = 0.033) and pERK2:ERK2 ($n_{+/+}$ = 3; $n_{A172V/+}$ = 3; unpaired t-test; t_4 = 2.93, p = 0.043) were observed in *Mapk1*$^{A172V/+}$ forebrain samples at PD100. Data is presented as mean ± SEM.

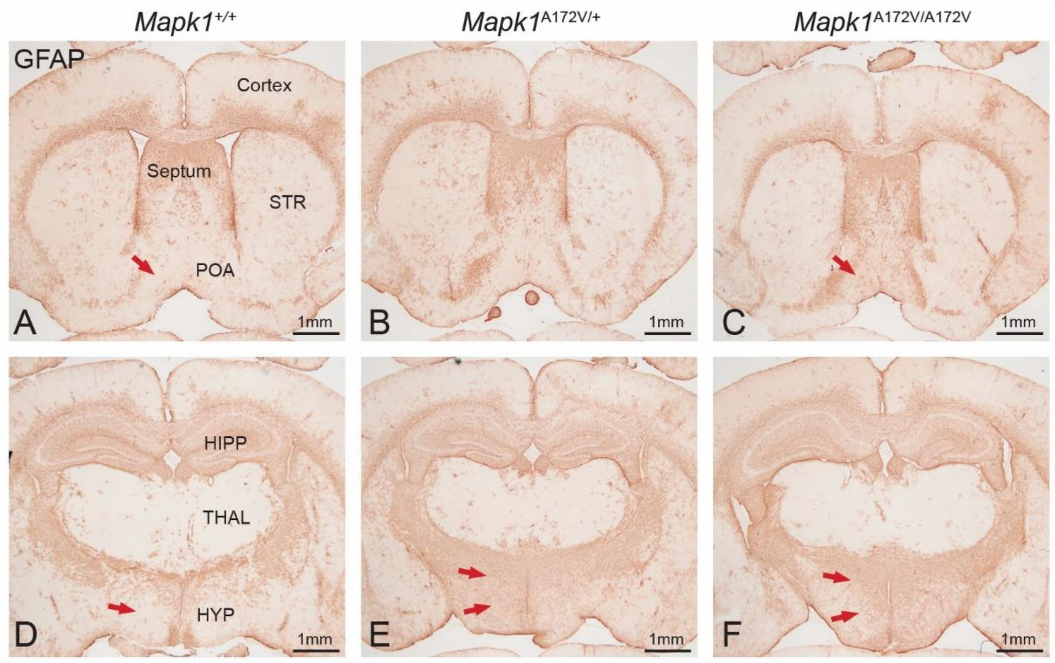

Figure S7. GFAP expression in the brains of *Mapk1* mutant and control mice. (A-C) Images showing GFAP expression (a marker for reactive astrocytes) in different brain structures in 10-week-old **(A)** *Mapk1*$^{+/+}$, **(B)** *Mapk1*$^{A172V/+}$, and **(C)** *Mapk1*$^{A172V/A172V}$ mice, including the cerebral cortex, septum, striatum (STR), and pre-optic area (POA). Red arrows indicate differences in GFAP immunoreactivity in the pre-optic area. **(D-F)** Images showing GFAP expression in **(D)** *Mapk1*$^{+/+}$, **(E)** *Mapk1*$^{A172V/+}$, and **(F)** *Mapk1*$^{A172V/A172V}$ mice in the hippocampus (HIPP), thalamus (THAL), and hypothalamus (HYP). Red arrows indicate differences in GFAP immunoreactivity in the hypothalamus. Scale bars are 1mm.

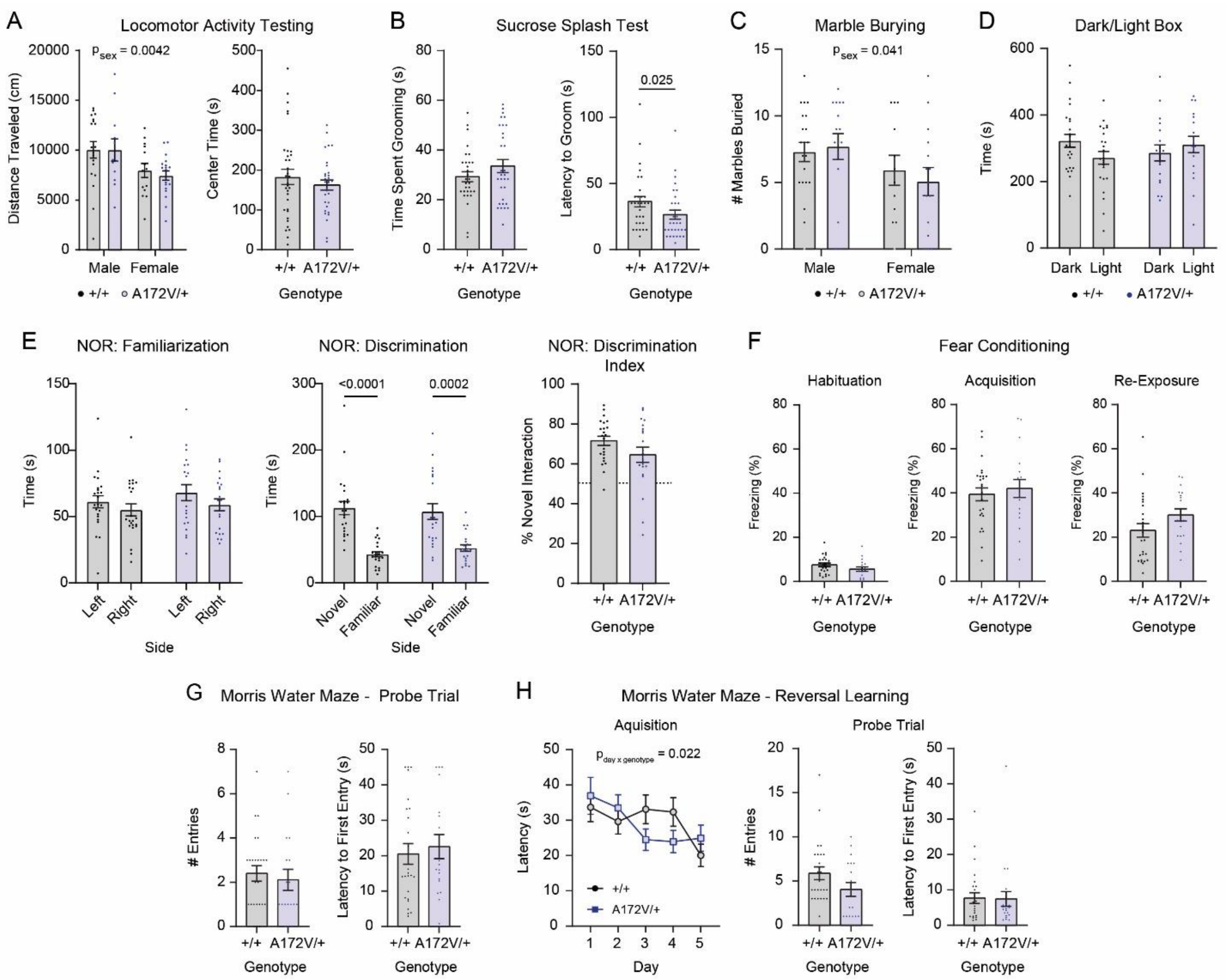

Figure S8. Behavioral testing of *Mapk1* mutant and control mice. (A) In a test of locomotor activity, there was a main effect of sex but not genotype (*left*; $n_{+/+}$ = 33, $n_{A172V/+}$ = 31; two-way ANOVA; $F_{1,60}$ = 0.098, $p_{sex\ x\ genotype}$ = 0.76; $F_{1,60}$ = 8.87, p_{sex} = 0.0042; $F_{1,60}$ = 0.12, $p_{genotype}$ = 0.73), and there were no changes in the time spent in the center of the arena (*right*; $n_{+/+}$ = 33, $n_{A172V/+}$ = 31; unpaired t-test with Welch's correction; $t_{55.23}$= 0.89, p = 0.38). **(B)** *Mapk1*$^{A172V/+}$ mice showed no significant changes in the amount of time spent grooming in the sucrose splash test (*left*; Mann-Whitney U-test; $U(n_{+/+}$ = 30, $n_{A172V/+}$ = 31) = 408, p = 0.42) but had a shorter latency to initiate grooming behavior (*right*; Mann-Whitney U-test; $U(n_{+/+}$ = 30, $n_{A172V/+}$ = 31) = 311.5, p = 0.025). **(C)** There was a significant main effect of sex but not genotype in the marble burying assay ($n_{+/+}$ = 33; $n_{A172V/+}$ = 31; two-way ANOVA; $F_{1,60}$ = 0.43, $p_{sex\ x\ genotype}$ = 0.52; $F_{1,60}$ = 4.35, p_{sex} = 0.041; $F_{1,60}$

= 0.055, $p_{genotype}$ = 0.82). **(D)** There were no effects of genotype or side in the dark/light box assay ($n_{+/+}$ = 24; $n_{A172V/+}$ = 18; two-way ANOVA; $F_{1,80}$ = 3.075, $p_{side\ x\ genotype}$ = 0.083; $F_{1,80}$ = 0.35, p_{side} = 0.56; $F_{1,80}$ = 0.0072, $p_{genotype}$ = 0.93). **(E)** No genotypic differences in object preference were observed during the familiarization phase of the novel object recognition (NOR) test (*left*; $n_{+/+}$ = 23; $n_{A172V/+}$ = 21; two-way repeated measures ANOVA; $F_{1,42}$ = 0.12, $p_{side\ x\ genotype}$ = 0.74; $F_{1,42}$ = 2.94, p_{side} = 0.094; $F_{1,42}$ = 1.021, $p_{genotype}$ = 0.32). During the discrimination phase, both genotypes interacted more with the novel object (*middle*; $n_{+/+}$ = 23; $n_{A172V/+}$ = 21; two-way ANOVA; $F_{1,42}$ = 0.69, $p_{object\ x\ genotype}$ = 0.41; $F_{1,42}$ = 49.37, p_{object} < 0.0001; $F_{1,42}$ = 0.065, $p_{genotype}$ = 0.80). There were no differences in the object discrimination index ((time spent with novel object – time spent with familiar objects) / total time interacting with both objects) between genotypes (*right*; $n_{+/+}$ = 23; $n_{A172V/+}$ = 21; unpaired t-test with Welch's correction; $t_{33.03}$= 1.58, p = 0.12). **(F)** During contextual fear conditioning, there were no significant differences between genotypes during the habituation phase before shocks were delivered (*left*; $n_{+/+}$ = 25, $n_{A172V/+}$ = 18; unpaired t-test; t_{41}= 1.55, p = 0.13), acquisition phase when shocks were presented (*middle*; $n_{+/+}$ = 25, $n_{A172V/+}$ = 18; unpaired t-test; t_{41}= 0.54, p = 0.59), or during context re-exposure 24-hours later (r*ight*; $n_{+/+}$ = 25, $n_{A172V/+}$ = 18; unpaired t-test; t_{41}= 1.62, p = 0.11). **(G)** During the probe trial of the Morris water maze (MWM), there were no significant differences in the number of entries into the target quadrant (*left*; Mann-Whitney U-test; $U(n_{+/+}$ = 25, $n_{A172V/+}$ = 18) = 194.5, p = 0.42) or latency to the first entry into the target quadrant (*right*; Mann-Whitney U-test; $U(n_{+/+}$ = 25, $n_{A172V/+}$ = 18) = 199, p = 0.53). **(H)** During the reversal learning acquisition phase of the MWM, there was a significant interaction between day and genotype (left; $n_{+/+}$ = 25, $n_{A172V/+}$ = 17; two-way ANOVA; $F_{4,160}$ = 2.95, $p_{day\ x\ genotype}$ = 0.022; $F_{3.73,149.1}$ = 5.26, p_{day} = 0.0007; $F_{1,40}$ = 0.024, $p_{genotype}$ = 0.88). *Post hoc* analysis determined that in wild-type mice, there were significant differences in latency on day 5 compared to their latency on days 1, 3, and 4 (Bonferroni *post hoc* test; p < 0.05), while no differences were observed in

Mapk1$^{A172V/+}$ mice (Bonferroni *post hoc* test, $p > 0.05$). During the reversal learning probe trial, there were no differences in entries into the target quadrant (*middle*; Mann-Whitney U-test; $U(n_{+/+} = 25, n_{A172V/+} = 15) = 120.5$, $p = 0.060$) and no difference in the latency to enter the target quadrant (right; Mann-Whitney U-test; $U(n_{+/+} = 25, n_{A172V/+} = 15) = 184$, $p = 0.93$). Data is presented as mean ± SEM.
